# Side chain effect in the modulation of α_v_β_3_/α_5_β_1_ integrin activity via clickable isoxazoline-RGD-mimetics: development of molecular delivery systems

**DOI:** 10.1038/s41598-020-64396-4

**Published:** 2020-05-04

**Authors:** Lucia Ferrazzano, Dario Corbisiero, Eleonora Potenza, Monica Baiula, Samantha Deianira Dattoli, Santi Spampinato, Laura Belvisi, Monica Civera, Alessandra Tolomelli

**Affiliations:** 1 Department of Chemistry “G.Ciamician”, University of Bologna, Via Selmi 2, 40126 Bologna, Italy; 2 Department of Pharmacy and Biothecnology, FABIT, University of Bologna, Via Irnerio 48, 40126 Bologna, Italy; 3 Department of Chemistry, University of Milano, Via Golgi 19, 20133 Milano, Italy

**Keywords:** Chemical biology, Medicinal chemistry, Organic chemistry, Chemical synthesis

## Abstract

Construction of small molecule ligand (SML) based delivery systems has been performed starting from a polyfunctionalized isoxazoline scaffold, whose α_v_β_3_ and α_5_β_1_ integrins’ potency has been already established. The synthesis of this novel class of ligands was obtained by conjugation of linkers to the heterocyclic core via Huisgen-click reaction, with the aim to use them as “shuttles” for selective delivery of diagnostic agents to cancer cells, exploring the effects of the side chains in the interaction with the target. Compounds **17b** and **24** showed excellent potency towards α_5_β_1_ integrin acting as selective antagonist and agonist respectively. Further investigations confirmed their effects on target receptor through the analysis of fibronectin-induced ERK1/2 phosphorylation. In addition, confocal microscopy analysis allowed us to follow the fate of EGFP conjugated α_5_β_1_ integrin and **17b** FITC-conjugated (compound **31**) inside the cells. Moreover, the stability in water solution at different values of pH and in bovine serum confirmed the possible exploitation of these peptidomimetic molecules for pharmaceutical application.

## Introduction

In the last decades, a major challenge in cancer chemotherapy has been the discovery of a “magic bullet” to kill tumors without affecting the healthy tissues, through highly selective treatments^1,2^. It is well known that standard chemotherapies are strongly limited by high toxicities against normal tissues, leading to severe side-effects for the patients. Unfortunately, reduction of the administered doses often results in failure of the therapy. Identification of molecular and physiological differences between healthy and cancerous tissues has recently allowed the design of agents capable to selectively deliver cytotoxic molecules to cancer cells, thereby reducing the off-target effects of traditional chemotherapy^3^.

These composite systems can be divided into three components: a targeting unit, a linker bond and a toxic agent^4^. The targeting ligand should be capable of selective binding to tumor-overexpressed antigens in order to recognize cancer cells. Among the number of different receptors and proteins involved in tumor physiology, α_v_β_3_ and α_5_β_1_ integrins are highly expressed on activated endothelial cells in several tumors, playing predominant roles in tumor-induced angiogenesis and growth^5^. For this reason, peptides and peptidomimetics designed to mimic the recognition sequence RGD (Arg-Gly-Asp)^6^, present in the extracellular endogenous ligands of these receptors, have received great attention as targeting motives^7^. Their exploitation as therapeutic tools, per se, is still an option but some recent unsatisfactory results^8^ prompted the researchers to turn their attention towards the possible use of these ligands as shuttles for selective delivery of therapeutic payloads and diagnostics^9^. To this purpose, the molecules should possess the proper pharmacophore for effective ligand-receptor interaction but also a peripheral and chemically functionalizable anchorage for covalent ligation of diverse spacers and bioactive units. The final backbone and conformation, anyway, should mirror the one of the lead candidates to guarantee the bioactivity. To date, drug-targeting has been successfully realized by the conjugation of potent anticancer drugs with monoclonal antibodies (mAb)^10,11^, furnishing compounds that are currently on the market. On the other hand, small molecules–drug conjugates (SMDCs) have been proposed as a possible alternative since they may be cheaper than biologics and display a better pharmacokinetic profile^12^.

The design of the second component, i.e. the linker, should take into account the fundamental issues of payload release. While for diagnostics, the imaging agent has to be strongly bound to the targeting unit for a specific detection, when a drug delivery system is designed, the *in vivo* rate of drug release from the conjugate and the tissue distribution pattern prior to that release have to be considered^13^. The ideal and smartest linker should be stable in blood circulation and extracellular sites, to avoid premature and unselective release of the drug, but it has to be easily cleavable when the tumor cells have been reached, affording *in situ* the native cytotoxic agent with restored efficacy. Among the variety of chemical structures applied as linkers, many are acid labile bonds (esters and hydrazones) that may be hydrolyzed in endosomal compartments, or specific peptide bonds recognized by lysosomal enzymes^14^. Following this approach, RGD-containing peptides have been successfully conjugated with diverse anticancer drugs, as doxorubicin, doxsaliform, monomethylauristatin E, cisplatin, camptothecin and paclitaxel^15,16^.

**Figure 1:**
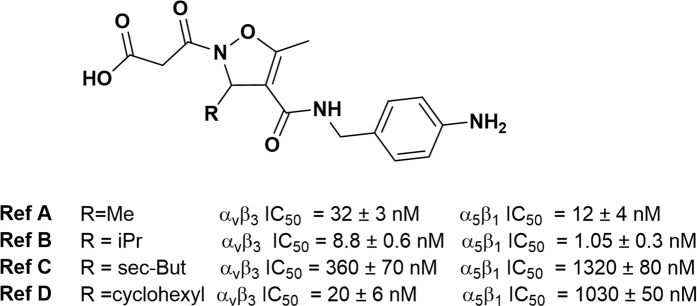
Potency of already reported isoxazoline-containing ligands measured in cell adhesion assay mediated by α_v_β_3_ and α_5_β_1_ integrins. (ref. Cell adhesion assay in ChemMedChem 2011, 6, 2264–2272).

An important issue is also the increase in water solubility of the drug due to peptide or peptidomimetic conjugation that often leads to favorable availability of the drug^17^. Recently we have reported the synthesis and biological evaluation of a small library of isoxazoline-based RGD mimetics where the pharmacophores were mimicked by malonic acid and aniline moieties^18^. Despite the known tox-risk associated to the presence of aniline, the molecules we already reported showed very good efficacy in binding the target receptors, probably as consequence of the basicity and length of this arginine-mimicking group. All the members of the library, differing only for the substituent in position 3 of the heterocycle, displayed excellent potency to modulate cell adhesion mediated by α_v_β_3_ integrins (Fig. 1).

On the basis of this consideration, we selected the functionalization in position 3 as a useful anchorage of the heterocyclic scaffold for covalent ligation of diverse linkers, with the aim to not compromise the integrin binding capabilities. The purpose of this investigation is the synthesis of a variety of discretely sized ligand-linker systems to demonstrate the broad utility of the isoxazoline ligands for diverse and efficient bioconjugation strategies in drug and diagnostic tumor homing.

## Results and discussion

### Chemistry

**Scheme 1:**
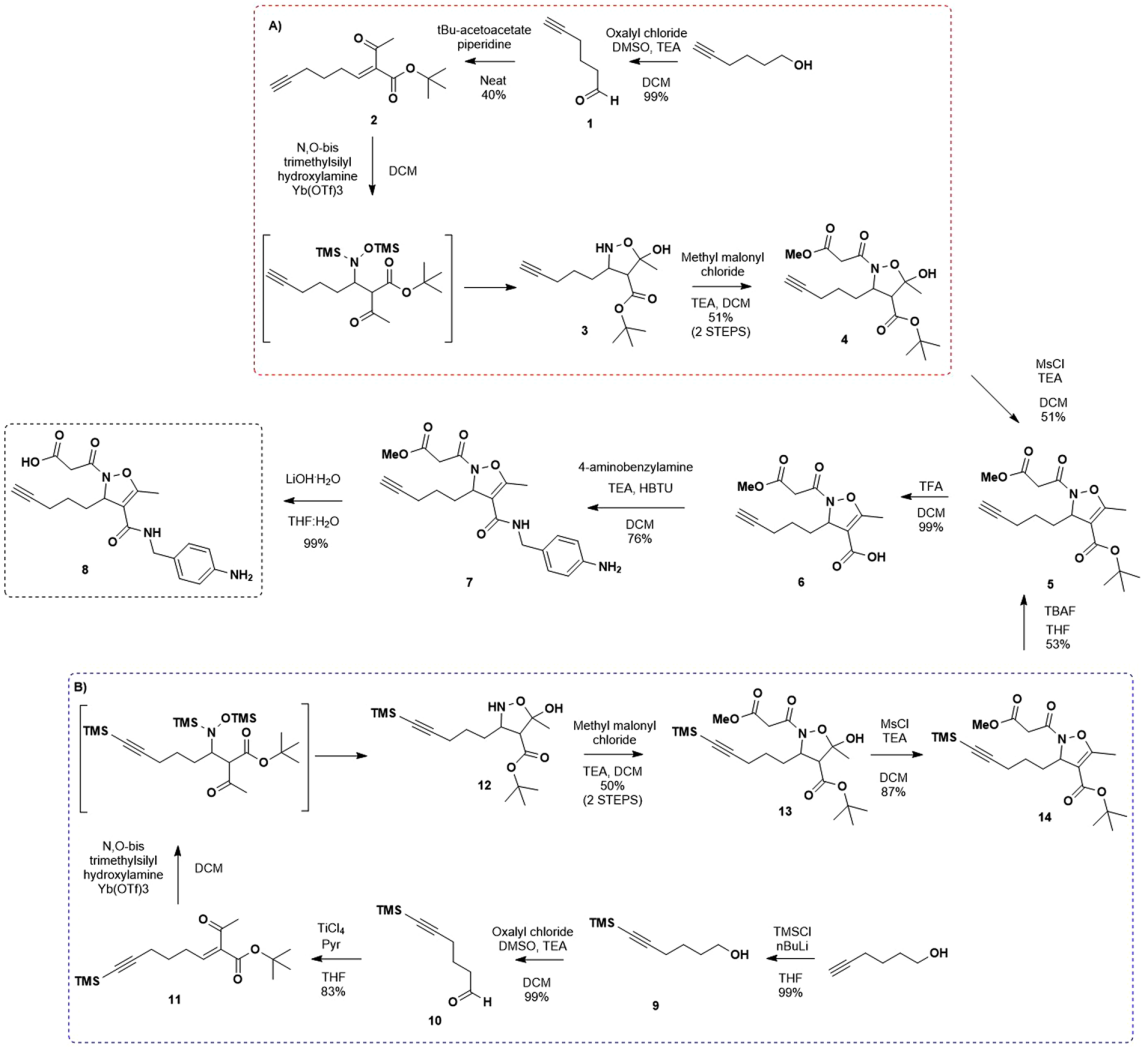
Synthetic pathways for the synthesis of methyl ester **7** and the corresponding acid **8**, using the route (**A**) or (**B**) to evaluate the effects of alkyne-side chain on the yield of some critical steps of reaction.

To introduce a functionalizable chain in position 3 of the isoxazoline scaffold, we thought that a terminal alkyne could be a versatile moiety to be exploited in 1,3-dipolar Huisgen cycloaddition with different azide-linkers. To follow our previously reported synthetic protocol, 5-hexynal **1** had to be synthesized from the corresponding commercially available alcohol, via Swern oxidation with oxalyl chloride and TEA in DMSO (99% yield, Scheme 1). The Knövenagel reaction between **1** and t-butyl-acetoacetate afforded in 40% yield the alkylidene acetoacetate **2** as a 1/4 mixture of *Z/E* isomers (Scheme 1, path A). The unsatisfactory yield, never observed previously for simpler aldehydes, even using a microwave assisted protocol^19^, was attributed to alkyne side reactions. For this reason, we protected the alkyne moiety with TMS group as reported by Cruciani and co-workers^20^. As a consequence, the Knövenagel reaction was performed using TiCl_4_/pyridine affording the alkyne-protected alkylidene acetoacetate **11** in 83% yield as 1/4 mixture of *Z/E* isomers as well and confirming the previous hypothesis of alkyne side reactivity (Scheme 1, path B). Reaction conditions for the addition of bis-(*N*,*O*)-trimethylsilylhydroxylamine to **2** and **11** were optimized on the basis of our previous experience^21,22^, in order to avoid the formation of oxime by-product as a result of the undesired 1,2-addition process^23^. According to these considerations, the reaction was performed in DCM in the presence of a catalytic amount (5%) of ytterbium triflate as Lewis acid. It should be noticed that the TMS protecting group of the hydroxylamine was removed during the usual work up procedure, inducing the fast conversion of the intermediate adduct to trans 5-hydroxyisoxazolidine-4-carboxylate **3** or **12**, as a single trans epimer, via intramolecular hemiketalisation. Introduction of the malonic pharmacophore was performed at this stage by acylation at the nitrogen with methyl malonyl chloride, in the presence of TEA. The isoxazolidines **4** or **13** were isolated in 51% and 50% respectively overall yield after two steps. Dehydration to the unsaturated racemic isoxazolines **5** or **14** was accomplished by mesylation of the hydroxyl group followed by base induced elimination. The removal of TMS protecting group on alkyne moiety of **14** by tetrabutylammonium fluoride afforded the product **5** in 53% yield. Selective removal of the t-butyl ester was then accomplished by treatment of **5** with an excess of trifluoroacetic acid in dichloromethane. The arginine mimetic chain was introduced by reaction of the acid **6** with 4-aminobenzylamine, following standard coupling conditions (HBTU/TEA in DCM) to give **7** in 76% yield. Hydrolysis of the methyl ester required a particular effort, since the undesired removal of the malonic side chain easily occurred, favored by the following spontaneous aromatization of the heterocycle as confirmed by LC-MS analysis. After several trials under different conditions, excellent results were obtained with a 7·10^−3^ M solution of LiOH·H_2_O in a 2:1 mixture of THF/water, following the reaction evolution by TLC, and stopping it at the disappearance of the starting ester. The acid **8** was obtained in quantitative yield.

**Scheme 2:**
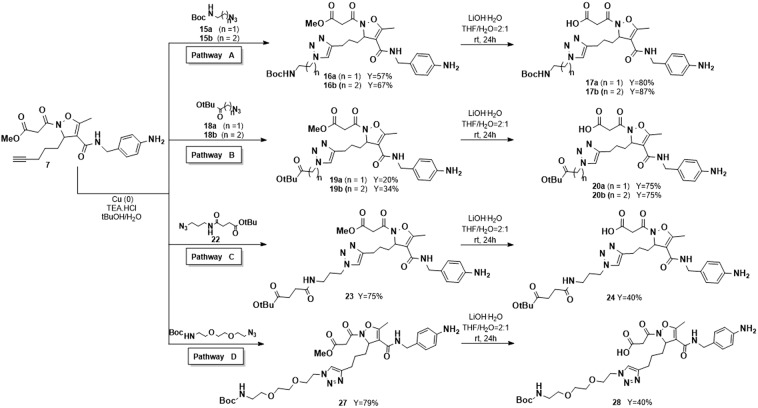
Synthesis of ligands **17a,b**, **20a,b**, **24** and **28** functionalized by Huisgen 1,3-dipolar cycloaddition with copper (0) powder, TEA·HCl in tBuOH/H_2_O, starting from intermediate 7.

In the design of the ligand-linker library, molecules terminating with an amine were prepared in order to obtain carriers for molecules with carboxylates or thiocarboxylates as active functionalization, as for instance the cytotoxic agent fumagillin or the fluorescein isothiocyanate diagnostic derivatives. To this purpose *N*-Boc-2-azido-ethylamine **15a** and *N*-Boc-3-azido-propylamine **15b** were prepared from the corresponding 2-bromo-ethylamine and 3-bromo-propylamine by protection of the amino moiety followed by substitution of the bromide with NaN_3_. The two azides were then reacted with methyl ester **7** in the presence of 10% copper (0) powder and TEA·HCl salt at room temperature in a 1:1 mixture of t-BuOH and water^24^. The click reaction led to the exclusive formation of the desired 1,4-disubstituted [1,2,3]-triazoles **16a** and **16b**^25^ in 57% and 67% yield respectively. Removal of the methyl ester under the above reported conditions afforded compounds **17a** (80%) and **17b** (87%) (Scheme 2, pathway A). Initially, the *N*-Boc protection at the linker amine moiety was retained in order to avoid the possible interference of a second amine group in the ligand-receptor binding. Moreover, it may mimic a carbamate linked payload.

For the preparation of molecules terminating with a carboxylate, able to conjugate drugs or diagnostics possessing functionalizable amines or hydroxyl groups, as for instance paclitaxel, compound **7** was submitted to Huisgen reaction in the presence of t-butyl 2-azido-acetate **18a** and t-butyl 3-azido-propionate **18b**, easily prepared from the corresponding bromides. The cycloaddition afforded compounds **19a** and **19b** in low yield (20% and 34% respectively).

**Scheme 3:**
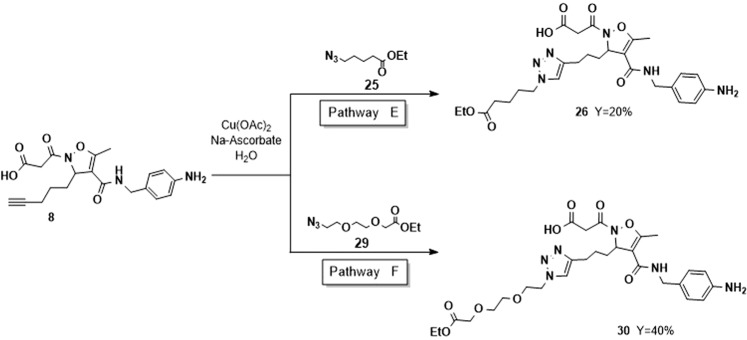
Synthesis of ligands **26** and **30** functionalized by Huisgen 1,3-dipolar cycloaddition with Cu(OAc)_2_, Na-Ascorbate in H_2_O, starting from intermediate **8**.

Complete regiocontrol was observed also in this case, affording exclusively the 1,4-disubstituted triazoles. Selective removal of the methyl ester was accomplished as reported above to afford acids **20a** and **20b** (Scheme 2, pathway B). Even in this case, the t-butyl group was retained to avoid interferences in the binding and to mimic the ester connection with a payload. In order to verify if elongation of the linker could turn into a lower interference in integrin-ligand binding, we planned to synthesize more complex systems. The assembly of these composite molecules could be faced through several protocols, differing for the order of formation of the strategic bonds. Therefore, the synthetic protocol was optimized for each specific substrate. To obtain compound **24**, having the succinic moiety as a typical and stable spacer between the carrier and the payload^26^, 3-bromo-propylamine was coupled with mono t-butyl pentandioic acid and the bromide was substituted with NaN_3_, to afford compound **22** in good yield (90%). Then, the click reaction with methyl ester **7** was performed under the usual conditions (75% yield). Removal of the methyl ester moiety, afforded the acid **24** in 40% yield (Scheme 2, pathway C). The synthesis of the simpler compound **26** was accomplished in one step by performing the Huisgen reaction on the acid **8** with ethyl 5-azido-pentanoate. Due to the nature of substrate **8**, the conditions of the reaction were modified and Cu(OAc)_2_ was used in the presence of sodium ascorbate^27^. The click reaction afforded **26** in 20% yield, as consequence of the difficulties in the purification of the product from the copper salts (Scheme 3, pathway E).

Many examples in the literature report the use of PEG as linker for drug-ligand connection for its ability to increase the solubility and to decrease the immunogenicity of the products^28^. Moreover, PEG-drug conjugates often exhibit a favourable *in vivo* behaviour and are less prone to enzymatic digestion. These data prompted us to design further carriers, containing the PEG fragment. To this purpose, compound **7** was reacted with commercially available *N*-Boc-1-amino-3,6-dioxa-8-octaneazide (Boc-NH-PEG(2)-N_3_) as reported in Scheme 2 (pathway D). Compound **27**, isolated in 79% yield was then transformed into the corresponding acid **28** under the usual hydrolytic conditions (Scheme 2).

**Scheme 4:**
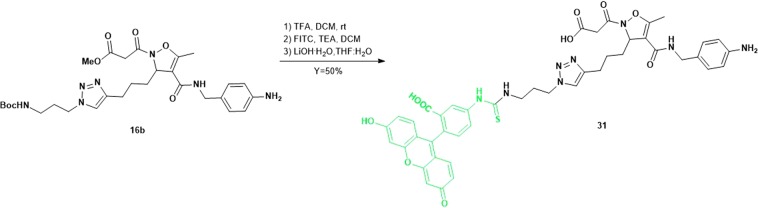
Synthesis of targeting delivery system **31** by conjugation of intermediate **16b** with FITC.

Finally, the acid **8** was treated with the PEG-azide **29**, easily prepared from the corresponding commercially available acid. As previously observed, the nature of the starting isoxazoline suggested the use of Cu(OAc)_2_ and sodium ascorbate as catalysts (Scheme 3, pathway F). Under these conditions compound **30** was isolated in 40% yield given the difficult purification step. Fluorescent labelled molecules are useful for localization of proteins, visualization of intracellular processes and study of interactions between ligand and receptors. In particular, imaging by fluorescence provides many advantages in terms of selectivity and sensitivity of the detection. For this purpose, compound **17b**, showing good selectivity and potency toward α_5_β_1_ integrins, was subjected to further functionalization with a diagnostic dye. To this purpose, its precursor (**16b**) was deprotected at the *N*-terminal position of the side chain by using TFA in DCM, and conjugated with FITC (fluorescein isothiocyanate) in presence of an excess of TEA in DCM. The FITC-labelled intermediate was subjected without further purification to the basic hydrolysis of the methyl ester, following the procedure previously reported, to afford the final compound **31** with 50% yield over three steps (Scheme 4).

### Pharmacological studies

#### Cell adhesion assay

In our previous experience, isoxazoline-based integrin ligands showed a strong potency towards α_v_β_3_ and α_5_β_1_ integrins, not affected by changing the alkyl group linked to position 3^18^. In designing this novel small library of peptidomimetics, alkyl chains were substituted by polyfunctionalized linkers including the triazole ring and amide, carbamate or ester moieties, that may create further interactions with the binding pocket. The ability of the synthesized racemic ligands to inhibit the adhesion of K562 cells (human erythroleukemic cells, expressing α_5_β_1_ integrin) or SK-MEL-24 cells (human malignant melanoma cells, expressing α_v_β_3_ integrin) to immobilized fibronectin was evaluated^29^. These cell models are widely used to investigate potential antagonists/agonists of α_5_β_1_ or α_v_β_3_ integrin-mediated cell adhesion^30–35^. In these experiments, the cells were seeded onto plates coated with fibronectin and allowed to adhere before quantitation of the number of adherent cells, in presence of increasing concentrations of the compounds. As a negative control, under these conditions, no significant cell adhesion was observed for BSA-coated plates or nonspecific substrate-coated plates (i.e., collagen I for SK-MEL-24 expressing α_v_β_3_ and poly-L-lysine for K562 expressing α_5_β_1_ integrin, data not shown). The ability of the new compounds to inhibit the adhesion of SK-MEL-24 and K562 cells to fibronectin was compared with that of the standard antagonist compounds Ac-Asp-Arg-Leu-Asp-Ser-OH (Ac-DRLDS) and H-Gly-Arg-Gly-Asp-Thr-Pro-OH (GRGDTP), known to be potent inhibitors of cell adhesion mediated by α_v_β_3_ and α_5_β_1_ integrins respectively^36^, and standard agonist Ref E (entry 15, Table 1), a potent and selective α_5_β_1_ ligand^37^. The obtained results are summarized in Table 1. From the results, it may be observed that the potency towards integrin α_v_β_3_ is generally maintained, while few compounds show high potency towards α_5_β_1_. Compounds terminating with a carbamate moiety (**17a**, **17b** and **28**) behave as antagonists to integrin receptors, displaying anyway different selectivity. In particular, **17a** displayed similar potency towards both receptors (entry 2), while the longer **17b** is an excellent selective ligand for α_5_β_1_ (entry 3), displaying IC_50_ in the low nanomolar range. The opposite preference could be observed for compound **28** that showed a good potency for α_v_β_3_ receptor (entry 8). On the other hand, compounds having a terminal ester group (**20a**, **20b**, **24**, **26**) in the side chain, behaved generally as agonists, inducing an increase in cell adhesion. All the molecules having a linear chain linked to the triazole ring (**20a**, **20b**, **26**) displayed high affinity for α_v_β_3_ integrin (entries 4, 5 and 7) in the sub-micromolar range. By introducing an amide moiety in the central part of an elongated linker, as in compound **24**, the opposite selectivity was observed, still maintaining an agonist effect (entry 6). The introduction of a short PEG fragment induced complete loss of the activity (compound **30**, entry 9). To complete the study, we also performed cell adhesion assay with the alkyne intermediate **8**, which showed a good potency and selectivity for α_v_β_3_. These data seem to suggest a possible influence of the terminal moiety on the agonist/antagonist role. A structure-activity relationship rationalization is still elusive, because agonist/antagonist behaviour^38^ for integrin ligands is known to depend also on the concentration^39,40^. Anyway, the good to excellent potency of the members of this class of peptidomimetics confirms their possible use as shuttle for drugs or diagnostic selective delivery to cells overexpressing these two classes of integrins.

**Table 1:** Effects of isoxazoline ligands on α_v_β_3_ and α_5_β_1_ integrin-mediated cell adhesion to Fibronectin (FN).

Entry	Compound	α_v_β_3_ IC_50_/EC_50_ (µM)^a^	α_5_β_1_ IC_50_/EC_50_ (µM)^a^
1	**8**	0.83 ± 0.05 *agonist*	>1000
2	**17a**	0.88 ± 0.07 *antagonist*	0.28 ± 0.03 *antagonist*
3	**17b**	>1000	0.0027 ± 0.0004 *antagonist*
4	**20b**	0.068 ± 0.011 *agonist*	>1000
5	**20a**	0.23 ± 0.08 *agonist*	>1000
6	**24**	>1000	6.59 ± 0.16 *agonist*
7	**26**	0.61 ± 0.08 *agonist*	>1000
8	**28**	8.84 ± 0.14 *antagonist*	>1000
9	**30**	>1000	>1000
10	**31**	n.d.	0.13 ± 0.01 *antagonist*
11^b^	Ref A	0.032 ± 0.003 *antagonist*	0.012 ± 0.004 *antagonist*
12^b^	Ref B	0.0088 ± 0.0006 *antagonist*	0.000105 ± 0.0003 *antagonist*
13^b^	Ref C	0.360 ± 0.070 *antagonist*	1.320 ± 0.080 *antagonist*
14^b^	Ref D	0.020 ± 0.006 *antagonist*	1.030 ± 0.050 *antagonist*
15^c^	Ref E	>1000	0.0129 ± 0.0006 *agonist*
16^d,e,f^	Ac-DRLDS	0.025 ± 0.003 *antagonist*	>1000
17^d,f^	GRGDTP	0.926 ± 0.006 *antagonist*	0.00062 ± 0.00009 *antagonist*
18 ^g^	c(RGDfV)	0.146 ± 0.043 *antagonist*	n.d.
19 ^h^	Cilengitide c(RGDf(NMe)V)	0.00058 ± 0.00001 *antagonist*	n.d.

[a] Data are presented as EC_50_ for agonists and as IC_50_ for antagonists (µM). Values are the mean ± SD of three independent experiments carried out in quadruplicate.

[b] Compounds reported in Fig. 1 and extracted from reference^18^.

[c] Ref E = 2-(2-(4-Oxo-(*o*-tolylcarbamoyl)azetidin-2-yl)acetamido)acetic acid. Extracted from reference^37^.

[d] Reference compounds.

[e] See reference^41^.

[f] See reference^38^.

[g] See reference^32^ and Mas-Moruno *et al. Anticancer Agents Med Chem*. 2010, 10(10), 753–768.

[h] See reference Dechantsreiter *et al. J. Med. Chem*. 1999, 42, 3033–3040.

n.d.= not determined.

#### Analysis of α_5_β_1_ integrin-mediated ERK phosphorylation

To gain further information about the agonist/antagonist role of our peptidomimetic integrin ligands and to verify the effect on intracellular signalling, we investigated the effect of most active compounds **17b** and **24** on fibronectin-induced phosphorylation of ERK1/2 in K562 cells, which express α_5_β_1_ integrin. The mechanism by which components of extracellular matrix generate intracellular signalling through integrins requires indeed an increased phosphorylation of cytoplasmatic second messengers. Phosphorylation of ERK1/2 plays a central role in fibronectin-mediated survival signalling through integrins: in fact, cell adhesion activates ERK1/2 by binding of α_5_β_1_ integrins at the cell surface to extracellular matrix proteins such as fibronectin. The experiment was performed by serum-starving K562 cells in RPMI-1640 containing 1% FBS for 16 h; thereafter, they were preincubated with compound **17b**, **24** (10^−7^–10^−9^ M) or the vehicle for 60 minutes and then plated for 60 minutes on fibronectin.

**Figure 2:**
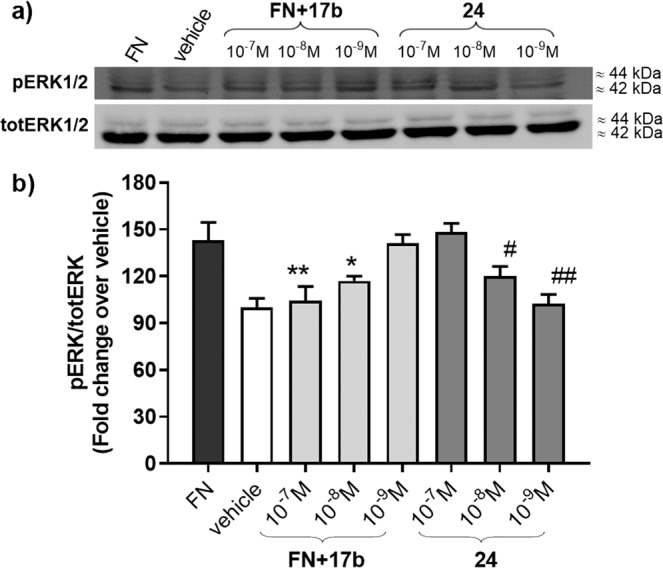
(**a**) Cropped blots related to the effect of compounds **17b** and **24** on α_5_β_1_ integrin-mediated phosphorylation of ERK1/2 in K562 cells. Cells were serum-starved in RPMI-1640 containing 1% FBS for 16 h; then cells were preincubated with different concentrations of the antagonist **17b** (10^−7^–10^−9^ M) or its vehicle for 1 h in suspension and then were allowed to adhere for 1 h on fibronectin (FN). Cells treated with the agonist **24** (10^−7^–10^−9^ M) were not incubated with fibronectin. Thereafter cells were lysed and lysates were analysed in Western blot using an antibody directed against phosphorylated ERK1/2 (pERK1/2) or total ERK1/2 (totERK1/2). Western blot showed that cells plated on FN had a much stronger signal for phosphorylated ERK1/2 than vehicle-treated cells. Compound **17b** prevented FN-induced phosphorylation of ERK1/2 in a concentration-dependent manner, while agonist **24** significantly increased ERK1/2 phosphorylation. Full-length blots are presented in Supplementary Figure S40. (**b**) Densitometric analysis of the bands (Mean ± SEM; n = 4); the amount of pERK1/2 is normalized to that of totERK1/2. *p < 0.05, **p < 0.001 vs. FN; #p < 0.05. ##p < 0.01 vs. vehicle (Newman-Keuls test after ANOVA).

When K562 cells were exposed to fibronectin for 60 minutes a much stronger signal was observed for phosphorylated ERK1/2, in comparison to vehicle-treated cells (Fig. 2).

Preincubation with compound **17b** (10^−7^–10^−9^ M) caused a significant concentration-dependent reduction in the amount of fibronectin-induced ERK1/2 phosphorylation levels in K562 cells (Fig. 2), thus confirming a significant effect of the ligand binding on intracellular signalling cascade. On the contrary, when K562 cells, not preincubated with fibronectin, were exposed to compound **24**, a significant concentration dependent increase in ERK1/2-phosphorylation was recorded thus confirming its agonistic behaviour (Fig. 2).

#### Integrin internalization

**Figure 3:**
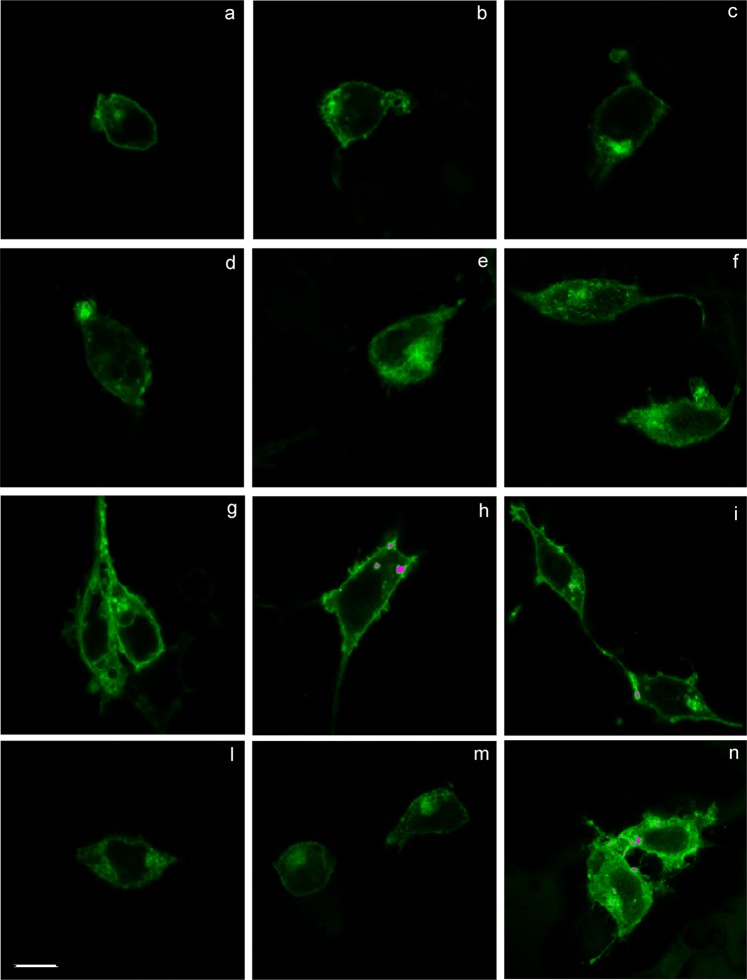
Confocal imaging on HEK293 cells transfected with α_5_-enhanced green fluorescent protein (EGFP) plasmid and treated with compounds **17b** or **24**. **Panels (a-c)** Vehicle-treated cells: integrins are located on the plasma membrane. **Panels (d-f)** FN-treated cells with 1 h incubation at 4 °C: α_5_β_1_ integrin was mainly located in the cytoplasm. **Panels (g-i)** FN-treated cells pre-incubated with the antagonist **17b** for 20 minutes at 4 °C: compound **17b** prevents FN-induced α_5_β_1_ internalization, being located in the cytoplasm. **Panels (l-n)** Cells treated with the agonist **24**: mimicking FN agonistic effect, compound **24** induced α_5_β_1_ integrin internalization. Scale bar: 10 µm. The images have been elaborated using NIS-Elements C Software.

Integrin trafficking is an important mechanism employed by cells to regulate integrin–extracellular matrix interactions, and thus cellular signalling, during processes such as cell migration and invasion^42^. α_5_β_1_ integrin is internalized, trafficked to recycling endosomes and then recycled to the plasma membrane^43,44^. Membrane trafficking pathways influence α_5_β_1_’s ability to promote invasion and metastasis^45,46^. In order to investigate the effects of peptidomimetics on integrin trafficking, α_5_β_1_ integrin internalization was observed by confocal microscopy on HEK293 cells transfected with α_5_-enhanced green fluorescent protein (EGFP) plasmid (Fig. 3), as these cells endogenously express β_1_ subunit^47,48^. HEK293 + α_5_-EGFP cells were treated with fibronectin (10 µg/mL) alone or in combination with the antagonist **17b** (1 µM) or with the agonist **24** (1 µM) alone. As shown in Fig. 3, after 15 minutes of treatment with the endogenous agonist fibronectin, α_5_β_1_ integrin was mainly localized in the cytoplasm (Fig. 3, panels d-f), if compared with vehicle-treated cells in which the integrin is quite completely located on the plasma membrane (Fig. 3, panels a-c). Moreover, when HEK293 + α_5_-EGFP cells were pre-treated with the antagonist **17b** before the addition of fibronectin, it prevented integrin internalization, that remained mainly located on the membrane (Fig. 3, panels g-i). On the contrary, compound **24**, mimicking the agonist behaviour of fibronectin, induced α_5_β_1_ internalization (Fig. 3, panels l-n): in fact, the integrin was mostly localized in the cytoplasm.

#### Stability assays

**Figure 4:**
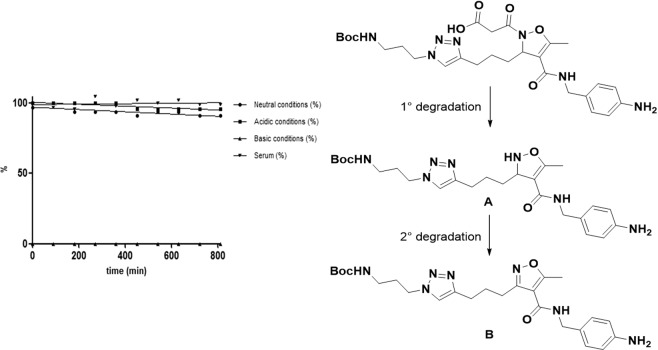
Stability of **17b** in neutral, basic, acidic and physiological conditions and its possible degradation pathway. Conditions: **17b** (0.1 mmol) in 300 µl of solvent (water or bovine serum). 5 eq of LiOH·H_2_O or HCl 1 M were added.

The ligand **17b**, which showed best results in the pharmacological evaluation, was subjected to stability assays at neutral (pH=7), basic (pH=10) and acidic (pH=3) conditions, as representative of the class of compounds. LC-MS injections were performed after 30 min from the preparation of the samples and then after each 1.5 hours, following the experiments for 14 h (Fig. 4). During the last synthetic step, the methyl ester deprotection, the amidic bond between the heterocycle and malonyl group showed high sensitivity to the basic environment and, as expected, the hydrolysed A was detected in traces as the unique degradation product. As a consequence of the spontaneous aromatization of A, also an increasing amount of B was observed after a short period. The aromatised form B was tested to evaluate its effect on the activity of selected integrins but, as consequence of the absence of a crucial pharmacophore, it was not active. This behaviour doesn’t represent a big issue since strong basic conditions are quite unusual in physiological environment. In fact under stress conditions with a large excess of base, the decomposition of **17b** to A occurred in few minutes. On the contrary, during the acidic treatment and under neutral conditions, **17b** resulted quite stable and after several hours only a 5% loss of product was detected, in favour of the formation of A and B. Finally, the stability of **17b** was also checked in bovine serum, confirming that no significant degradation occurs in this physiological medium.

#### Confocal microscopic observation of isooxazoline FITC-conjugated distribution

**Figure 5:**
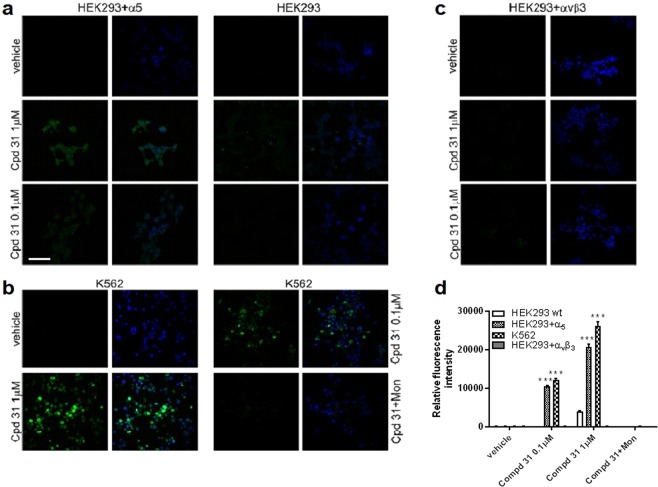
Confocal microscopy images of HEK293 or HEK293 + α_5_, HEK293 + αvβ_3_ and K562 cells treated with compound **31** (Cpd 31). Cells were exposed to compound **31** (0.1–1 μM) or its vehicle for 60 minutes at 4 °C and thereafter cells were moved to 37 °C for 15 minutes to promote integrin internalization and fixed as described in Methods. a) Compound **31**, conjugated with FITC was able to enter the cells in α_5_ integrin-dependent manner: it was internalized in HEK293 + α_5_ but not in HEK293 cells. Right Panels: HEK293 cells not expressing α_5_ integrin subunit. Left Panels: HEK293 + α_5_. b) Concentration-dependent internalization of compound **31** was observed also in K562 cancer cells, endogenously expressing α_5_β_1_ integrin. Treatment with monensin (an inhibitor of integrin trafficking, 2 µM for 2 h) significantly inhibited compound **31** internalization in K562 cells. c) Compound **31** was not able to enter in HEK293 + α_v_β_3_ cells, not expressing α_5_β_1_. In the left column of each panel green (FITC) fluorescence was shown, whereas in right one green and blue (DAPI) signals were merged. Nuclei were counterstained with DAPI. Scale bar: 30 µm. d) Compound **31** internalization was evaluated according to green fluorescence intensity in the cells and is reported in the graph. (Mean ± SEM; n = 2). ***p < 0.001 vs vehicle treated cells (Newman-Keuls test after ANOVA). The images have been elaborated using NIS-Elements C Software.

In order to visualize the localization of isoxazolines inside the cells and to explore the possibility to use them as shuttles for selective delivery of therapeutic payloads and diagnostics, compound **31** was synthesized conjugating **17b** with FITC, as described above. This FITC conjugated isoxazoline maintained the ability to reduce K562 cells adhesion in a concentration-dependent manner, similar to compound **17b**, but with a lower potency (IC_50_: 0.13 ± 0.01 μM). To study intracellular localization of compound **31**, HEK293 cells were transfected with a plasmid coding for α_5_ integrin subunit (HEK293 + α_5_), as they do not express this subunit endogenously^47,48^ but they express β_1_. When HEK293 + α_5_ cells were treated with **31** (0.1–1 μM) we observed that the compound was localized in the cell cytoplasm, as it could be internalized (Fig. 5). In addition, the internalization of compound **31** was concentration-dependent: at a higher concentration (1 μM) it accumulated inside the cells in a greater extent. Moreover, we observed that the internalization of compound **31** is α_5_ integrin-mediated because it was not able to enter inside HEK293 cells that do not express α_5_ integrin endogenously (Fig. 5a).

In order to establish the possibility for compound **31** to be internalized also in cancer cells, endogenously expressing α_5_β_1_, K562 cells, derived from chronic myelogenous leukemia, were exposed to compound **31** (0.1–1 μM), as reported in Methods section. As shown in Fig. 5b, compound **31** was internalized in K562 cells in a concentration-dependent way.

In addition, to confirm that compound **31** is internalized through an active mechanism of endocytosis, K562 cells were exposed for 2 h to monensin (2 µM), which blocks the trafficking of α_5_ integrin from the Golgi stack to the trans Golgi network^49^. Inhibition of integrin trafficking prevented compound **31** internalization in K562 cells (Fig. 5b); these results demonstrate that compound **31** enters cells relying on internalization rather than on passive permeability.

As compound **31** derived from the conjugation of FITC to compound **17b** that was a specific α_5_β_1_ integrin ligand, to confirm that the selectivity was maintained, we treated HEK293 cells transfected with plasmids coding for α_v_ and β_3_ subunits (HEK293 + α_v_β_3_) with compound **31** (0.1–1 μM) (Fig. 5c). In cells not expressing α_5_β_1_ integrin compound **31** was not able to be internalized. These results confirm from one hand the α_5_β_1_-dependent internalization mechanism of compound **31** and from the other hand displayed that the specificity towards α_5_β_1_ of compound **17b** was maintained although conjugation with FITC molecule.

### Computational studies

**Figure 6:**
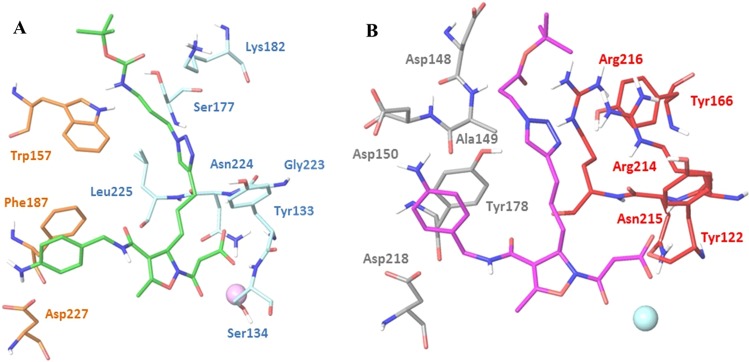
(**A**) Docking best pose of **(*R*)-17b** (green) into the crystal structure of the extracellular domain of α_5_β_1_ integrin (α_5_ orange, β_1_ blue). Only selected integrin residues involved in the interactions with the ligand are shown. The metal ion at MIDAS is displayed as a magenta CPK sphere. Non polar hydrogens are hidden for better visual representation. (**B**) Docking best pose of **(*R*)-20b** (purple) into the crystal structure of the extracellular domain of α_v_β_3_ integrin (α_v_ grey, β_3_ red). The metal ion at MIDAS is displayed as a cyan CPK sphere. The image has been generated using Maestro graphical interface [Maestro, version 10.5, Schrödinger, LLC, New York, NY, 2016].

To rationalize the pharmacological activity observed for the new isoxazoline-RGD-mimetics, compounds **17b**, **20b** and **24** were selected to investigate the effects of the side chain in position 3 of the isoxazoline core on the interaction with the target receptors. In the family of the selective α_5_β_1_ ligands, we chose the most active compound **17b** which inhibits α_5_β_1_ integrin-mediated cell adhesion at low nM concentrations, and compound **24** acting as an agonist of α_5_β_1_ receptor at µM levels. On the other hand, compound **20b** was chosen as selective agonist ligand of α_v_β_3_ integrin. Automated docking calculations were carried out with the Glide software package (version 7.0) by using the crystal structure of the extracellular segment of integrin α_5_β_1_ in complex with a disulfide-bonded cyclic RGD peptide (PDB code 4WK4) and the crystal structure of the extracellular domain of integrin α_v_β_3_ in complex with the cyclic pentapeptide Cilengitide (PDB code 1L5G), according to the procedures reported in the Experimental Section. As the docking approaches were successful in reproducing the crystallographic binding mode of the cyclic peptides at the interface of the α and β subunits by means of electrostatic and H-bond interactions, we applied the same docking protocols to both enantiomers of compounds **17b**, **20b** and **24** to generate computational models for the interaction of these ligands with α_5_β_1_ and α_v_β_3_ integrins and evaluate their ability to properly fit the receptor site. In all the calculations, the experimentally observed binding modes of the cyclic peptides with α_5_β_1_ and α_v_β_3_ integrins were used as reference models for the analysis of the docking results in terms of protein-ligand interactions. Docking results pointed out that the (*R*) and (*S*) enantiomers of the new functionalized isoxazolines show different behavior, with better performances exhibited by the (*R*)-isomer, especially in the α_5_β_1_ integrin. Based on the number of docking poses reproducing the key interactions of the X-ray complex, **(*R*)-17b** was the best α_5_β_1_ ligand confirming the pharmacological results. In the best pose of **(*R*)-17b** (as well as in ten other poses) into the α_5_β_1_ binding pocket, the carboxylate group of the ligand is coordinated to the metal cation in the MIDAS region of the β_1_ subunit, while the aniline moiety forms H-bond interactions with the negatively charged side chain of Asp227 in the α_5_ subunit (Fig. 6, left). Further stabilizing interactions involve the formation of H-bonds between the ligand carboxylate group and the backbone amide hydrogen of Asn224 and Ser134 (and Tyr133 in some poses) in the β_1_ unit, and π-stacking interactions between the ligand aromatic group and the α_5_-Phe187 side chain. A H-bond between the Boc carbonyl moiety of ligand and the amino group of β_1_-Lys182 side chain is also observed. The long-chain substituent (bearing the triazole ring) at the position 3 of the isoxazoline is well accommodated at the interface between the α and β subunit; in particular, the triazole ring establishes stabilizing contacts with the side chains of residues α_5_-Trp157, β_1_-Tyr133, β_1_-Ser177 and β_1_-Lys182 (Fig. 6, left). On the contrary, docking results show that most poses of compound **17b** (both *R* and *S* enantiomers) into α_v_β_3_ lack the H-bond interaction between the ligand aniline moiety and the side chain of α_v_-Asp218, and display an unfavorable arrangement of the long-chain substituent at the position 3 of the isoxazoline at the α/β integrin interface. Due to residue differences between the binding sites, the long chain bearing the triazole ring of compound **17b** can fit unhindered only into the pocket available at the α_5_β_1_ interface, thus confirming the pharmacological results in terms of selectivity. A comparison of α_5_β_1_ and α_v_β_3_ integrins highlights that the mutations of α_5_-Phe187 into α_v_-Tyr178 and of α_5_-Asp227 into α_v_-Asp218 might allow the aniline moiety of the ligand to maintain the same H-bond and π-stacking interactions in the two binding sites. Other mutations at the α/β interface modify size and shape of the pocket accessible to the isoxazoline substituent, thus affecting the ligand binding mode (see Supplementary Figure S41). Molecular dynamics simulations allowing partial receptor flexibility showed that **(*R*)-17b** maintains stable interactions with α_5_β_1_, similar to those observed in the docking poses. In particular, the long-chain substituent is firmly placed at the α/β integrin interface, with the triazole and the Boc carbonyl moiety engaged in interactions with residues α_5_-Trp157, β_1_-Tyr133 and β_1_-Lys182. Instead, the docking poses of compound **24** reveal some difficulties in establishing the key interactions with the α_5_β_1_ pocket, mainly due to the lack of a simultaneous favorable fitting of the long-chain substituent at the α/β interface. Compared to **17b** a reduced number of docking poses reproducing the X-ray interactions is achieved and MD simulations confirm high mobility of **(*R*)-24** in the binding pocket. The binding determinants of **20b** were finally investigated. The docking poses of **(*R*)-20b** into α_v_β_3_ integrin are characterized by the interaction of the carboxylate group with the metal cation in the MIDAS region and the Asn215 and Tyr122 residues in the β_3_ subunit, and by the interaction of the aniline moiety with the side chains of α_v_-Asp218 or α_v_-Asp150, and of α_v_-Tyr178. Moreover, the Boc carbonyl moiety of the substituent at the position 3 of the isoxazoline can create H-bonds with the backbone amide hydrogen of α_v_-Ala149 or the guanidinium group of β_3_-Arg216, while the triazole ring can form H-bond or cation-π interactions with the side chain of β_3_-Arg214 (Fig. 6, right). Molecular dynamics simulations allowing partial receptor flexibility showed that **(*R*)-20b** maintains stable interactions with α_v_β_3_, especially those involving the acid pharmacophoric group and the triazole ring. In short, according to these results, only the subtle fitting of suitable features of the isoxazoline substituent with appropriate receptor traits at the α/β integrin interface seems to allow the optimal interaction of both the pharmacophoric moieties and the functionalizable chain of the isoxazoline-RGD-mimetics with the integrin binding site.

## Conclusions

The synthesis of a small library of ligands obtained by conjugation of polyfunctionalized linkers to isoxazoline ring via Huisgen-click reaction, allowed to verify the possibility to use these scaffolds as α_v_β_3_ and α_5_β_1_ targeting motifs, with the aim to use them as “shuttles” for selective delivery of therapeutics and diagnostics to cancer cells. The different behavior of the members of the library seems to suggest a correlation between the terminal moiety of the linker chain and the cell adhesion inhibition or activation, even if a rationale in structure-activity relationship is not predictable and needs further investigation. Compound **17b**, that showed excellent potency towards α_5_β_1_ integrin in the nanomolar range as antagonist, was selected for further investigation to establish the effect on fibronectin induced ERK phosphorylation. It was able to prevent fibronectin-induced α_5_-integrin-mediated ERK1/2 intracellular signaling activation and α_5_-integrin internalization.

Moreover, compound **31**, isoxazoline FITC-conjugated derived from compound **17b**, confirmed the possibility to exploit these integrin ligands as “shuttles” for the selective delivery of therapeutics and diagnostics as consequence of its internalization only inside integrin expressing cells.

Finally, stability in water solution at different values of pH and in bovine serum was verified in order to confirm the potential exploitation of these peptidomimetic molecules for pharmaceutical applications.

## Methods

All chemicals were purchased from commercial suppliers and used without further purification. Anhydrous solvents were purchased in sure seal bottles over molecular sieves and used without further drying. Flash chromatography was performed on silica gel (230–400 mesh). NMR Spectra were recorded with Varian Mercury Plus 400 or Unity Inova 600 MHz spectrometers. Chemical shifts were reported as δ values (ppm) relative to the solvent peak of CDCl_3_ set at δ = 7.27 ppm (^1^H-NMR) or δ = 77.0 ppm (^13^C-NMR), CD_3_OD set at δ = 3.31 ppm (^1^H-NMR) or δ = 49.0 ppm (^13^C-NMR), D_2_O set at δ = 4.79 ppm (^1^H-NMR), CD_3_CN set at δ = 1.93 ppm (^1^H-NMR) or δ = 117.7 ppm (^13^C-NMR), (CD_3_)_2_CO set at δ = 2.04 ppm (^1^H-NMR) or δ = 29.8 ppm (^13^C-NMR). Coupling constants are given in Hertz. LC-MS analyses were performed on an HP1100 liquid chromatograph coupled with an electrospray ionization-mass spectrometer using a Phenomenex Gemini C18–3 µ -110 Å column, H_2_O/CH_3_CN as neutral solvent at 25 °C or H_2_O/CH_3_CN with 0.2% formic acid as acid solvents (positive scan 100–500 m/z, fragmentor 70 eV). Another set of experiments has been performed on an HP1100 liquid chromatograph coupled with an electrospray ionization-ion trap mass spectrometer MSD1100 using a Phenomenex Zorbax C18–3.5 µ -80 Å column, H_2_O/CH_3_CN with 0.08% trifluoroacetic acid as acid solvents (positive scan 100–500 m/z, fragmentor 70 eV). Compounds 9 and 10 were synthesized following a known procedure and analytical data are in agreement with the literature. Analyses indicated by the symbols of the elements or functions were within ±0.4% of the theoretical values.

### Synthesis

#### General procedure for the Swern oxidation: (1) and (10)

In a 2-neck round bottom flask, equipped to perform reaction under N_2_ atmosphere, a solution of dimethyl sulfoxide (2.36 ml, 33.3 mmol) in DCM (8.2 ml) was added at −78 °C to a solution of oxalyl chloride (1.47 ml, 17.4 mmol) in DCM (33.4 ml), and the mixture was stirred for 5 min. After this time, the alcohol (14.5 mmol) in DCM (16.4 ml) was added dropwise in 5 min and the solution was then stirred for 15 min. TEA dry (10.1 ml, 72.5 mmol) was added dropwise and the mixture was stirred further at room temperature for 10 min. The mixture was diluted with 60 ml of Et_2_O and 30 ml of water. The organic layer, after dilution with Et_2_O, was washed with NH_4_Cl sat. (20 ml x 3). It was then dried over Na_2_SO_4_ and evaporated under reduced pressure to give the product as a yellow oil and used without further purifications.

**(1)** Y > 99%. Anal. Calcd. C_6_H_8_O (96.96): C 74.97 H 8.39. ^1^H-NMR (400 MHz, Chloroform-d) δ 9.79 (t, *J* = 1.3 Hz, 1 H), 2.59 (dt, *J* = 1.3, 7.2 Hz, 2 H), 2.26 (dt, *J* = 2.6, 7.2 Hz, 2 H), 1.96 (t, *J* = 2.6 Hz, 1 H), 1.84 (m, 2 H); ^13^C-NMR (400 MHz, Chloroform-d) δ 200.1, 82.2, 68.6, 45.2, 20.0, 16.7.

#### Tert-butyl-2-acetyloct-2-en-7-ynoate (2)

In a one-neck round bottom flask, 1 (18.1 mmol), tert-butyl acetoacetate (2.96 ml, 18.1 mmol) and piperidine (269 µl, 2.7 mmol) were stirred at room temperature for 24 h. The mixture was diluted with EtOAc and washed with water (10 ml x 3). The organic layer was dried over Na_2_SO_4_ and then evaporated. The crude was purified with flash chromatography (silica gel, 99/1 cyclohexane/EtOAc) to obtain the product as a 1/4 mixture of *E/Z* isomers.

**(2)** Y = 40%. Anal. Calcd. C_14_H_20_O_3_ (236.14): C 71.29 H 8.56. 1H-NMR (400 MHz, Chloroform-d) δ 6.77 (t, *J* = 8.0 Hz, 1 H, *E* isomer), 6.74 (t, *J* = 7.6 Hz, 1 H, *Z* isomer), 2.42 (dt, *J* = 7.6, 7.2 Hz, 2 H), 2.29 (s, 3 H), 2.23 (dt, *J* = 2.4, 6.8 Hz, 2 H), 1.96 (t, J = 2.4 Hz, 1 H), 1.72 (m, 2 H), 1.53 (s, 9 H); ^13^C-NMR (400 MHz, Chloroform-d) δ 194.8, 165.5, 145.1, 138.6, 83.2, 82.1, 69.2, 28.6, 28.0, 27.0, 26.8, 18.0; LC-MS: rt=9.1 min, m/z: 259 [M + 23], 275 [M + 39], 495 [2 M + 23].

#### Tert-butyl 2-acetyl-8-(trimethylsilyl)oct-2-en-7-ynoate (11)

A 1 M solution of TiCl_4_ in DCM (29 ml, 29 mmol) was added dropwise to THF (53 ml) at 0 °C and the mixture was stirred for few minutes. Then tert-butyl acetoacetate (2.4 ml, 14.5 mmol) in THF (6.3 ml) and **9** (14.5 mmol) in THF (6.3 ml) were added at 0 °C; the mixture was stirred for 75 min. A solution of pyridine (5.1 ml, 58 mmol) in THF (10.1 ml) was then added dropwise in 90 min and the mixture stirred overnight. The solution was diluted with Et_2_O (60 ml) and washed with water (20 ml x 3) and brine (15 ml x 3). The organic layer was dried over Na_2_SO_4_ and then evaporated. The crude product was purified by flash chromatography (silica gel, 98/2 cyclohexane/EtOAc) to obtain the product as a 1/4 mixture of *E/Z* isomers.

**(11)** Y = 83%. Anal. Calcd. C_17_H_28_O_3_Si (308.18): C 65,95 H 9,15. 1H-NMR (400 MHz, Chloroform-d) δ 6.75 (t, *J* = 8.0 Hz, 1 H, *E* isomer), 6.73 (t, *J* = 7.6 Hz, 1 H, *Z* isomer), 2.39 (dt, *J* = 7.6, 7.6 Hz, 2 H), 2.28 (s, 3 H), 2.24 (m, 2 H), 1.68 (m, 2 H), 1.53 (s, 9 H), 0.12 (s, 9 H); ^13^C-NMR (400 MHz, Chloroform-d) δ 201.0 (*E*), 194.8 (*Z*), 165.5 (*Z*), 163.3 (*E*), 145.8 (*E*), 145.4 (*Z*), 138.4 (*Z*), 137.9 (*E*), 105.9 (*Z* + *E*), 85.2 (*Z* + *E*), 82.1 (*Z*), 81.7 (*E*), 28.7 (*Z* + *E*), 28.0 (*Z*), 27.9 (*E*), 27.8 (*E*), 27.4 (*E*), 27.2 (*Z*), 26.8 (*Z*), 19.5 (*Z*), 19.3 (*E*), 0.0 (*Z* + *E*); LC-MS: rt=12.5 min, m/z: 331 [M + 23], 347 [M + 39], 639 [2 M + 23].

#### General procedure for the synthesis of isoxazolidine ring (3) and (12)

In a 2-necked round bottom flask, equipped to perform reaction under N_2_ atmosphere, Yb(OTf)_3_ (76 mg, 0.123 mmol) was added to a solution of **2** or **11** (2.46 mmol) in DCM (45 ml), and the mixture stirred for 10 min at r.t. After this time, *N*,*O*-bistrimethylsilyl hydroxylamine (1.05 ml, 4.92 mmol) was added at 0 °C and the mixture stirred for 30 min. The solution was diluted with DCM and washed with water (10 ml x 3), giving the crude product as yellow oil used without further purifications.

#### General procedure for the synthesis of acylated isoxazolidine ring (4) and (13)

In two-necked round bottom flask, equipped to perform reaction under N_2_ atmosphere, TEA (514 µl, 3.69 mmol) and methyl malonyl chloride (396 µl, 3.69 mmol) were added at 0 °C to a solution of **3** or **12** (2.46 mmol) in DCM (12 ml). The mixture was stirred at room temperature for 2,5 h. The mixture was washed with water (10 ml x 3). The crude product was purified with flash chromatography (silica gel, 80/20 cyclohexane/EtOAc). The product was isolated as a yellow oil.

**(4)** Y = 51% (over two steps). Anal. Calcd. C_18_H_27_NO_7_ (369.18): C 58.62 H 7.37 N 3.79. ^1^H-NMR (400 MHz, Chloroform-d) δ 4.65 (dt, *J* = 6.8, 7.4 Hz, 1 H), 3.72 (s, 3 H), 3.59 (d, *J* = 15.5 Hz, 1 H), 3.53 (d, *J* = 15.6 Hz, 1 H), 2.86 (d, *J* = 7.4 Hz, 1 H), 2.28–2.19 (m, 2 H), 1.92 (t, *J* = 2.4 Hz, 1 H), 1.83–1.74 (m, 2 H), 1.63 (s, 3 H), 1.64–1.52 (m, 2 H), 1.46 (s, 9 H); ^13^C-NMR (400 MHz, Chloroform-d) δ 169.3, 168.4, 167.5, 105.3, 83.7, 82.6, 68.7, 61.6, 59.1, 52.3, 40.7, 33.9, 27.8, 24.6, 22.8, 17.8. LC-MS: t = 8.3 min, m/z: 370 [M + 1], 392 [M + 23], 761 [2 M + 23].

**(13)** Y = 50% (over two steps). Anal. Calcd. C_21_H_35_NO_7_Si (441.22): C 57.11 H 7.97 N 3.17. ^1^H-NMR (400 MHz, Chloroform-d) δ 4.64 (dt, *J* = 6.8, 7.2 Hz, 1 H), 3.67 (s, 3 H), 3.50 (s, 2 H), 2.82 (d, *J* = 7.2 Hz, 1 H), 2.21 (dt, *J* = 6.8, 6.0 Hz, 2 H), 1.72 (m, 2 H), 1.62 (s, 3 H), 1.59–1.47 (m, 2 H), 1.42 (s, 9 H), 0.07 (s, 9 H); ^13^C-NMR (400 MHz, Chloroform-d) δ 169.2, 168.5, 167.6, 106.7, 105.4, 84.8, 82.6, 61.7, 59.2, 52.4, 40.8, 34.0, 27.9, 24.8, 22.9, 19.4, 0.1. LC-MS: t = 11.3 min, m/z: 442 [M + 1], 464 [M + 23], 905 [2 M + 23].

#### Synthesis of isoxazoline (5) from (4) and isoxazoline (14) from (13)

In a two-necked round bottom flask, equipped to perform reaction under N_2_ atmosphere, TEA (530 µl, 3.8 mmol) and methanesulfonyl chloride (294 µl, 3.8 mmol) were added to a solution of **4** or **13** (1.9 mmol) in DCM (8 ml) at 0 °C, and the mixture stirred at room temperature for 24 h. The mixture was diluted with DCM and washed with water (10 ml x 3). The organic layer was dried over Na_2_SO_4_ and then evaporated under reduced pressure. The crude product was purified by flash chromatography (silica gel, 80/20 cyclohexane/EtOAc) giving the product as a white solid.

**(5)** Y = 51%. Anal. Calcd. C_18_H_25_NO_6_ (351.17): C 61.40 H 7.17 N 3.99. ^1^H-NMR (400 MHz, Chloroform-d) δ 5.30 (m, 1 H), 3.72 (s, 3 H), 3.58 (d, *J* = 15.9 Hz, 1 H), 3.46 (d, *J* = 15.9 Hz, 1 H), 2.29–2.14 (m, 2 H), 2.20 (s, 3 H), 1.99 (m, 1 H), 1.92 (t, *J* = 2.4 Hz, 1 H), 1.76 (m, 1 H), 1.64–1.53 (m, 2 H), 1.48 (s, 9 H); ^13^C-NMR (400 MHz, Chloroform-d) δ 166.8, 162.0, 161.0, 105.8, 83.8, 80.9, 68.4, 62.8, 52.2, 40.4, 32.4, 28.0, 26.7, 23.6, 17.8, 11.1. LC-MS: LC-MS: rt=10.1 min, m/z: 352 [M + 1], 374 [M + 23], 725 [2 M + 23].

**(14)** Y = 87%. Anal. Calcd. C_21_H_33_NO_6_Si (423.21): C 59.61 H 7.86 N 3.30. 1H-NMR (400 MHz, Chloroform-d) δ 5.30 (m, 1 H), 3.72 (s, 3 H), 3.58 (d, *J* = 15.9 Hz, 1 H), 3.46 (d, *J* = 15.9 Hz, 1 H), 2.24 (dt, *J* = 3.2, 7.2 Hz, 2 H), 2.20 (s, 3 H), 1.92 (m, 1 H), 1.71 (m, 1 H), 1.64–1.53 (m, 2 H), 1.48 (s, 9 H), 0.12 (s, 9 H); ^13^C-NMR (400 MHz, Chloroform-d) δ 167.0, 167.9, 162.2, 161.1, 106.9, 106.0, 84.6, 81.1, 63.1, 52.4, 40.6, 32.8, 28.2, 23.9, 19.5, 11.3, 0.1; LC-MS: rt= 13.2 min, m/z: 424 [M + 1], 446 [M + 23],462 [M + 39].

#### Synthesis of isoxazoline ring (5) from (14) by TMS protecting group removal

To a solution of **14** (440 mg, 1.1 mmol) in THF (2.3 ml), tetrabutylammonium fluoride trihydrate (656 mg, 2.08 mmol) was added and the mixture stirred overnight at room temperature. After adding 5 ml of H_2_O, THF was removed and the solution diluted with 10 ml of DCM. The organic layer was washed with water (5 ml x 3), dried over Na_2_SO_4_ and evaporated. The crude product was purified by flash chromatography (silica gel, 8/2 cyclohexane/EtOAc) to obtain the product as a yellow oil. Yield of **5**: 53%.

#### Removal of tert-butyl ester: synthesis of acid (6)

In one-necked round bottom flask, to a solution of **5** (378 mg, 1.08 mmol) in DCM (36 ml) trifluoroacetic acid (1.24 ml, 16.15 mmol) was added and the mixture stirred for 24 h. The solution was evaporated to obtain the desired product as brown oil.

**(6)** Y > 99%. Anal. Calcd. C_14_H_17_NO_6_ (295.11): C 56.82 H 5.82 N 4.74. 1H-NMR (400 MHz, Chloroform-d) δ 5.36 (m, 1 H), 3.73 (s, 3 H), 3.63 (d, *J* = 15.9 Hz, 1 H), 3.50 (d, *J* = 16.0 Hz, 1 H), 2.27 (s, 3 H), 2.20 (m, 2 H), 2.03 (m, 1 H), 1.93 (t, *J* = 2.6 Hz, 1 H), 1.78 (m, 1 H), 1.65–1.52 (m, 2 H); ^13^C-NMR (400 MHz, Chloroform-d) δ 168.3, 167.9, 167.0, 164.4, 104.2, 83.7, 68.7, 62.9, 52.6, 40.4, 32.3, 23.6, 18.0, 11.8; LC-MS: rt= 5.0 min, m/z: 296 [M + 1], 318 [M + 23], 334 [M + 39], 613 [2 M + 23].

#### Synthesis of isoxazoline- 4-amino benzylamide (7)

In two-necked round bottom flask, equipped to perform reaction under N_2_ atmosphere, TEA (226 µl, 1.62 mmol) and HBTU (614 mg, 1.62 mmol) were added to a solution of **6** (1.08 mmol) in DCM (7 ml), and the mixture was stirred at room temperature for 5 min. 4-aminobenzylamine (245 µl, 2.16 mmol) was then added and the mixture stirred for further 4 h. The mixture was diluted with DCM and washed with water (10 ml x 3). The organic layer was dried over Na_2_SO_4_ and then evaporated under reduced pressure. The crude product was purified by flash chromatography (silica gel, 80/20 cyclohexane/EtOAc) giving the product as a yellow oil.

**(7)** Y = 76%. Anal. Calcd. C_21_H_25_N_3_O_5_ (399.18): C 63.39 H 6.32 N 10.49. ^1^H-NMR (400 MHz, Chloroform-d) δ 7.06 (d, *J* = 8.0 Hz, 2 H), 6.63 (d, *J* = 8.0 Hz, 2 H), 5.62 (bs, 1 H), 5.27 (m, 1 H), 4.41 (dd, *J* = 5.6, 14.0 Hz, 1 H), 4.29 (dd, *J* = 5.2, 14.0 Hz, 1 H), 3.70 (s, 3 H), 3.58 (d, *J* = 15.9 Hz, 1 H), 3.44 (d, *J* = 16.0 Hz, 1 H), 2.38 (m, 1 H), 2.21 (m, 1 H), 2.02 (s, 3 H), 1.97 (m, 1 H), 1.65 (m, 1 H), 1.58–1.51 (m, 2 H); ^13^C-NMR (400 MHz, Chloroform-d) δ 168.5, 166.9, 162.0, 159.0, 145.9, 129.1, 127.6, 115.1, 106.5, 83.7, 69.3, 61.6, 52.4, 43.1, 40.5, 38.5, 32.6, 23.0, 17.3, 11.3; LC-MS: rt= 1.4 min, m/z: 400 [M + 1], 422 [M + 23], 438 [M + 39], 821 [2 M + 23].

#### General procedure for the hydrolysis of malonyl methyl ester. Synthesis of (8), (17a), (17b), (20a), (20b), (24),(28)

In a one-necked round bottom flask, LiOH^.^H_2_O (5.24 mg, 0,125 mmol) was added to a solution of ester (0.125 mmol) in a 2/1 mixture of THF/H_2_O (18.75 ml). The reaction was stopped after disappearance of the starting material, following the evolution by TLC. The solution was neutralized adding dropwise HCl 1 M solution and then water was evaporated. The product was purified by flash chromatography (C18 reverse phase silica gel, 8/2 water/CH_3_CN).

**(8)** Y = 99%. Anal. Calcd. C_20_H_23_N_3_O_5_ (385.16): C 62.08 H 6.00 N 10.89. ^1^H-NMR (400 MHz, Methanol-d4) δ 7.04 (d, *J* = 8.2 Hz, 2 H), 6.66 (d, *J* = 8.2 Hz, 2 H), 5.43 (m, 1 H), 4.84 (m, 2 H), 4.36 (d, *J* = 14.6 Hz, 1 H), 4.17 (d, *J* = 14.6 Hz, 1 H), 2.22–2.11 (m, 2 H), 2, 18 (s, 3 H), 1.87 (bs, 1 H), 1.86 (m, 1 H), 1.66 (m, 1 H), 1.57–1.48 (m, 2 H); ^13^C-NMR (400 MHz, Methanol-d_4_) δ 172.4, 172.0, 163.6, 158.3, 146.4, 128.3, 128.1, 115.2, 106.6, 83.2, 68.5, 62.3, 42.3, 39.9, 32.8, 23.5, 17.3, 10.0. LC-MS: rt= 1.6 min, m/z: 386 [M + 1], 400 [M + 23], 771 [2 M + 1].

**(17a)** Y = 80%. Anal. Calcd. C_27_H_37_N_7_O_7_ (571.28): C 56.85 H 6.51 N 17.13. ^1^H-NMR (400 MHz, Methanol-d_4_) δ 7.64 (s, 1 H), 7.61 (bs, 1 H), 7.01 (d, *J* = 8.3 Hz, 2 H), 6.90 (bs, 1 H), 6.65 (d, *J* = 8.3 Hz, 2 H), 5.45 (m, 1 H), 4.85 (m, 2 H), 4.39–4.32 (m, 3 H), 4.18 (d, *J* = 14.4 Hz, 1 H), 3.45 (t, J = 5.6 Hz, 2 H), 2.67 (t, J = 8.0 Hz, 2 H), 2.17 (s, 3 H), 1.87–1.53 (m 4 H), 1.37 (s, 9 H). 13C-NMR (400 MHz, Methanol-d4) δ 169.7, 163.4, 163.0, 161.9, 158.5, 147.3, 144.9, 129.3, 129.0, 121.9, 115.4, 107.3, 79.8, 61.3, 49.8, 42.7, 40.5, 38.8, 32.4, 28.3, 24.4, 23.2, 11.3; LC-MS: rt= 1.6 min, m/z: 572 [M + 1].

**(17b)** Y = 87%. Anal. Calcd. C_28_H_39_N_7_O_7_ (585.29): C 57.32 H 6.73 N 16.71. ^1^H-NMR (400 MHz, Methanol-d_4_) δ 7.69 (s, 1 H), 7.00 (d, *J* = 8.0 Hz, 2 H), 6.64 (d, *J* = 8.0 Hz, 2 H), 5.45 (m, 1 H), 4.35–4.31 (m, 3 H), 4.17 (d, *J* = 14.6 Hz, 1 H), 3.46 (d, *J* = 14.8 Hz, 1 H), 3.21 (d, *J* = 14.8 Hz, 1 H), 3.01 (t, *J* = 6.0 Hz, 2 H), 2.67 (t, *J* = 7.2 Hz, 2 H), 2.17 (s, 3 H), 2.01–1.98 (m, 2 H), 1.86–1.61 (m, 4 H), 1.40 (s, 9 H); ^13^C-NMR (400 MHz, Methanol-d_4_) δ 171.9, 169.5, 163.6, 161.7, 158.3, 147.4, 146.4, 135.7, 128.2, 122.0, 115.1, 106.5, 78.7, 62.3, 48.2, 42.2, 37.1, 32.8, 30.1, 29.2, 27.3, 24.3, 24.1, 10.0. LC-MS: rt= 1.6 min, m/z: 586 [M + 1], 608[M + 23].

**(20a)** Y = 75%. Anal. Calcd. C_26_H_34_N_6_O_7_ (542.25): C 57.54 H 6.33 N 15.51. ^1^H-NMR (400 MHz, Chloroform-d) δ 7.76 (s, 1 H), 7.47 (bs, 1 H), 7.41 (d, *J* = 8.0 Hz, 2 H), 7.28 (d, *J* = 8.0 Hz, 2 H), 5.51 (m, 1 H), 5.20 (s, 2 H), 4.50 (d, *J* = 15.2 Hz, 2 H), 4.39 (d, *J* = 15.2 Hz, 2 H), 2.76–2.69 (m, 2 H), 2.20 (s, 3 H), 1.85–1.60 (m, 4 H), 1.45 (s, 9 H); ^13^C-NMR (400 MHz, Chloroform-d) δ 173.6, 171.2, 169.9, 169.2,167.2, 147.0, 146.8, 134.1, 128.2, 122.4, 115.3, 106.6, 82.4, 61.0, 51.4, 43.2, 34.0, 33.3, 27.8, 24.6, 24.0,11.0; LC-MS (acid eluents): rt= 9.4 min, m/z: 543 [M + 1], 565 [M + 23].

**(20b)** Y = 75%. Anal. Calcd. C_27_H_36_N_6_O_7_ (556.26): C 58.47 H 6.51 N 15.14. ^1^H-NMR (400 MHz, Chloroform-d) δ 7.82 (bs, 1 H), 7.80 (s, 1 H), 7.44 (d, *J* = 8.0 Hz, 2 H), 7.34 (d, *J* = 8.0 Hz, 2 H), 5.50 (m, 1 H), 4.61–4.56 (m, 3 H), 4.46 (d, *J* = 12.4 Hz, 1 H), 2.90–2.85 (m, 2 H), 2.75–2.71 (m, 2 H), 2.20 (s, 3 H), 1.86–1.65 (m, 4 H), 1.38 (s, 9 H); ^13^C-NMR (400 MHz, Chloroform-d) δ 173.6, 170.8, 170.2, 169.6, 167.5, 147.0, 146.2, 133.8, 128.6, 122.0, 115.1, 106.4, 80.9, 60.7, 48.2, 43.4, 35.4, 33.9, 30.8, 27.6, 24.8, 24.2,11.5; LC-MS(acid eluents): rt= 10.0 min, m/z: 557 [M + 1], 579 [M + 23].

**(24)** Y = 40%. Anal. Calcd. C_31_H_43_N_7_O_8_ (641.32): C 57.85 H 6.73 N 15.27. ^1^H-NMR (400 MHz, Methanol-d_4_) δ 7.80 (s, 1 H), 7.43 (d, *J* = 8.0 Hz, 2 H), 7.35 (d, *J* = 8.0 Hz, 2 H), 5.52 (m, 1 H), 4.51 (d, *J* = 15.0 Hz, 1 H), 4.22–4.35 (m, 3 H), 3.18–3.13 (m, 2 H),2.78–2.68 (m, 2 H), 2.63–2.57 (m, 2 H), 2.48–2.44 (m, 2 H), 2.21 (s, 3 H), 2.08–2.03 (m, 2 H), 1,98–1,94 (m, 1 H), 1.87–1.83 (m, 1 H), 1.76–1.59 (m, 4 H), 1.41 (s, 9 H); ^13^C-NMR (400 MHz, Methanol-d_4_) δ 172.5, 172.2, 172.0, 169.1, 165.6, 162.7, 147.0, 146.5, 135.2, 128.6, 122.1, 115.3, 106.2, 80.8, 62.0, 48.4, 42.8, 37.0, 33.1, 31.2, 30.8, 29.4, 28.8, 28.0, 24.4, 24.0,11.0; LC-MS (acid eluents): rt= 9.4 min, m/z: 642 [M + 1], 664 [M + 23].

**(28)** Y = 28: 40%. Anal. Calcd. C_31_H_45_N_7_O_9_ (659.33): C 56.36 H 6.91 N 14.90. ^1^H-NMR (400 MHz Methanol-d_4_) δ 7.73 (s, 1 H), 7.03 (d, *J* = 8.4 Hz, 2 H), 6.67 (d, *J* = 8.4 Hz, 2 H), 5.47 (m, 1 H), 4.82 (m, 2 H), 4.51 (t, *J* = 5.2 Hz, 2 H), 4.37 (d, *J* = 14.6 Hz, 1 H), 4.20 (d, *J* = 14.6 Hz, 1 H), 3.87 (t, *J* = 5.2 Hz, 2 H), 3.60–3.57 (m, 4 H), 3.46 (t, *J* = 5.6 Hz, 2 H), 3.19 (t, *J* = 6.0 Hz, 2 H), 2.71 (t, *J* = 6.8 Hz, 2 H), 2.20 (s, 3 H), 1.85–1.58 (m, 4 H), 1.42 (s, 9 H); ^13^C-NMR (400 MHz, Methanol-d_4_) δ 175.4, 172.4, 170.3, 168.2, 158.2, 156.9, 147.3, 146.4, 128.4, 128.2, 122.5, 115.1, 107.1, 78.7, 70.0, 69.8, 69.6, 69.0, 49.8, 42.2, 42.1, 39.8, 33.3, 29.3, 27.3, 24.9, 10.3 LC-MS(acid eluents): rt= 9.9 min, m/z: 660 [M + 1], 682 [M + 23].

#### General procedure for the synthesis of azides (15a), (15b)

In two-necked round bottom flask, equipped to perform reaction under N_2_ atmosphere, to a solution of 2-bromo-ethylamine hydrobromide or 3-bromo-propylamine hydrobromide (2,44 mmol) in DCM (12.2 ml), Boc_2_O (510 µl, 2.22 mmol) and TEA (464 µl, 3.33 mmol) were added at 0 °C and the mixture stirred at room temperature overnight. The mixture was then washed with NH_4_Cl saturated solution, NaHCO_3_ saturated solution, brine and evaporated to afford the N-Boc-bromo amine intermediate as a colourless oil that was used without further purifications. The intermediate was dissolved in DMF (3.4 ml) and NaN_3_ (174 mg, 2.68 mmol) was added in one portion. The mixture was stirred at 60 °C for 4 h and then diluted with EtOAc and washed with water (10 ml x 3). The organic layer was evaporated after anhydrification over Na_2_SO_4_ to give the azides as yellow oils.

**(15a)** Y = 70%. Anal. Calcd. C_7_H_14_N_4_O_2_ (186.11): C 45.05 H 7.59 N 30.13. ^1^H-NMR (400 MHz, Chloroform-d) δ 4.79 (bs, 1 H), 3.40 (t, *J* = 5.7 Hz, 2 H), 3.28 (m, 2 H), 1.43 (s, 9 H). ^13^C-NMR (400 MHz, Chloroform-d) δ 155.8, 79.9, 50.8, 39.8, 28.2; LC-MS: rt= 6.5 min, m/z: 209 [M + 23], 372 [2 M + 1], 395 [2 M + 23].

**(15b)** Y = 90%. Anal. Calcd. C_8_H_16_N_4_O_2_ (200.13): C 47.88 H 8.06 N 27.99. ^1^H-NMR (400 MHz, Chloroform-d) δ 4.63 (bs, 1 H), 3.33 (t, *J* = 8.0 Hz, 2 H), 3.18 (m, 2 H), 1.71–1.77 (m, 2 H), 1.41 (s, 9 H). ^13^C-NMR (400 MHz, Chloroform-d) δ 162.2, 78.4, 48.7, 36.1, 31.0, 28.0; LC-MS: rt= 7.4 min, m/z: 223 [M + 23], 423 [2 M + 23].

#### Tert-butyl 4-((3-bromopropyl)amino)-succinate (21)

In two-necked round bottom flask, equipped to perform reaction under N_2_ atmosphere, to a solution of mono tert-butyl pentandioic acid (100 mg, 0.57 mmol) in DCM (4 ml), HBTU (324 mg, 0.86 mmol), TEA (120 µl, 0.86 mmol) and 3-bromo propylamine hydrobromide (130 mg, 0.63 mmol) were added and the mixture stirred at room temperature for 4 h. The mixture was diluted with DCM, washed with water (5 ml x 3) and evaporated. The crude product was purified by flash chromatography (silica gel, 9/1 cyclohexane/EtOAc).

**(21)** Y = 50%. Anal. Calcd. C_11_H_20_BrNO_3_ (293.06): C 44.99 H 6.84 N 4.74. ^1^H-NMR (400 MHz, Chloroform-d) δ 5.93 (bs, 1 H), 3.43–3.36 (m, 4 H), 2.62–2.52 (m, 2 H), 2.40 (t, *J* = 6.8 Hz, 2 H), 2.07 (m, 2 H), 1.44 (s, 9 H); ^13^C-NMR (400 MHz, Chloroform-d) δ 172.3, 172.2, 80.8, 38.0, 32.2, 31.1, 30.8, 30.7, 27.9; LC-MS: rt= 6.6 min, m/z: 316 [M + 23], 609 [2 M + 23].

#### General procedure for the synthesis of (18a), (18b), (22) and (25)

Bromo derivative (1 mmol) was dissolved in DMF (1.4 ml) and NaN_3_ (71 mg, 1.1 mmol) was added. The mixture was stirred at 60 °C for 4 h and then diluted with EtOAc and washed with water (5 ml x 3).

**(18a)** Y = 45%. Anal. Calcd. C_6_H_11_N_3_O_2_ (157.09): C 45.67 H 7.05 N 26.64. 1H-NMR (400 MHz, Chloroform-d) δ 3.72 (s, 2 H); 1.48 (s, 9 H); ^13^C-NMR (400 MHz, Chloroform-d) δ 167.2, 82.6, 50.6, 27.7. LC-MS: rt= 8.2 min, m/z: 180 [M + 23].

**(18b)** Y = 45%. Anal. Calcd. C_7_H_13_N_3_O_2_ (171.10): C 48.95 H 7.63 N 24.51. ^1^H-NMR (400 MHz, Chloroform-d) δ 3.50 (t, *J* = 6.4 Hz, 2 H); 2.48 (t, *J* = 6.8 Hz, 2 H); 1.45 (s, 9 H); ^13^C-NMR (400 MHz, Chloroform-d) δ 169.7, 80.7, 46.6, 35.9, 27.6. LC-MS: rt= 8.8 min, m/z: 194 [M + 23].

**(22)** Y = 90%. Anal. Calcd. C_11_H_20_N_4_O_3_ (256.15): C 51.62 H 7.88 N 21.90. ^1^H-NMR (400 MHz, Chloroform-d) δ 6.00 (bs, 1 H), 3.36–3.30 (m, 4 H), 2.56 (t, *J* = 6.8 Hz, 2 H); 2.40 (t, *J* = 6.8 Hz, 2 H), 1.80–1.72 (m, 2 H), 1.43 (s, 9 H); ^13^C-NMR (400 MHz, Chloroform-d) δ 172.4, 172.0, 80.8, 49.2, 37.0, 31.2, 30.8, 28.8, 28.0; LC-MS: rt= 6.0 min, m/z: 279 [M + 23], 535 [2 M + 23].

**(25)** Y = 85%. Anal. Calcd. C_7_H_13_N_3_O_2_ (171.10): C 49.03 H 7.66 N 24.56. ^1^H-NMR (400 MHz, Chloroform-d) δ 4.10 (q, *J* = 7.2 Hz, 2 H), 3.27 (t, *J* = 6.4 Hz, 2 H), 2.31 (t, *J* = 6.8 Hz, 2 H), 1.73–1.57 (m, 4 H), 1.23 (t, *J* = 7.2 Hz, 3 H); ^13^C-NMR (400 MHz, Chloroform-d) δ 172.9, 60.2, 50.9, 33.5, 28.2, 22.0, 14.1; LC-MS: rt= 8.0 min, m/z: 172 [M + 1], 194 [M + 23].

#### Ethyl 2-(2-(2-azidoethoxy)ethoxy)acetate (29)

[2-(2-azidoethoxy)ethoxy]acetic acid cyclohexylamine salt (250 mg, 0.87 mmol) was dissolved in Et_2_O (850 µl) and BF_3_^.^Et_2_O (107 µl, 0.87 mmol) was added at 0 °C. The mixture was refluxed for 3 h and then evaporated. The crude product was purified by flash chromatography (silica gel, 1/1 EtOAc/cyclohexane) affording the pure product as a colourless oil.

**(29)** Y = 40%. Anal. Calcd. C_8_H_15_N_3_O_4_ (217.11): C 44.36 H 6.96 N 19.38. ^1^H-NMR (400 MHz, Chloroform-d) δ 4.20 (q, *J* = 7.2 Hz, 2 H), 4.14 (s, 2 H); 3.75–3.72 (m, 2 H), 3.70–3.66 (m, 4 H), 3.38 (q, *J* = 5.2 Hz, 2 H), 1.27 (q, *J* = 7.2 Hz, 3 H); ^13^C-NMR (400 MHz, Chloroform-d) δ 170.2, 70.8, 70.7, 70.5, 68.6, 60.6, 50.5, 14.0. LC-MS: rt= 5.4 min, m/z: 218 [M + 1], 240[M + 23].

#### General procedure for the Huisgen reaction between methyl ester (7) and azides: (16a), (16b), (19a), (19b), (23), (27)

In one-necked round bottom flask, in a mixture of tBuOH:H_2_O = 1:1 (1 ml) TEA (0.15 mmol) and HCl 1 M (0.15 mmol) were added and stirred for 2 min. 7 (0.15 mmol), Cu(0) (0.015 mmol) and azide (0.15 mmol) were added and the mixture stirred at room temperature following the reaction by TLC. The mixture was filtered through celite to remove the copper powder and evaporated. The crude was purified by flash chromatography (silica gel, 9/1 EtOAc/cyclohexane).

**(16a)** Y = 57%. Anal. Calcd. C_28_H_39_N_7_O_7_ (585.29): C 57.42 H 6.71 N 16.74. ^1^H-NMR (400 MHz, Chloroform-d) δ 7.44 (bs, 1 H), 7.29 (s, 1 H), 7.13 (d, *J* = 8.2 Hz, 2 H), 6.66 (d, *J* = 8.2 Hz, 2 H), 5.45 (m, 1 H), 5.09 (bs, 1 H), 4.42–4.35 (m, 4 H), 3.69 (s, 3 H), 3.56 (d, *J* = 16.0 Hz, 1 H), 3.51 (m, 2 H), 3.46 (d, *J* = 16.0 Hz, 1 H), 2.72–2.67 (m, 2 H), 2.24 (s, 3 H), 1.85 (m, 1 H), 1.75 (m, 1 H), 1.61–1.55 (m, 2 H), 1.39 (s, 9 H); ^13^C-NMR (400 MHz, Chloroform-d) δ 168.9, 167.1, 162.2, 158.9, 155.9, 147.3, 144.9, 129.3, 129.0, 121.9, 115.4, 107.3, 79.8, 61.2, 52.4, 49.8, 42.7, 40.5, 32.3, 29.6, 28.3, 24.4, 23.2, 11.3; LC-MS: rt= 5.6 min, m/z: 586 [M + 1], 608 [M + 23].

**(16b)** Y = 67%. Anal. Calcd. C_29_H_41_N_7_O_7_ (599.31): C 58.08 H 6.89 N 16.35. ^1^H-NMR (400 MHz, Chloroform-d) δ 7.50 (bs, 1 H)7.35 (s, 1 H), 7.12 (d, *J* = 7.9 Hz, 2 H), 6.72 (d, *J* = 7.9 Hz, 2 H), 5.52 (m, 1 H), 4.88 (bs, 1 H), 4.42 (m, 2 H), 4.27 (t, *J* = 6.9 Hz, 2 H), 3.71 (s, 3 H), 3.58 (d, *J* = 16.0 Hz, 1 H), 3.48 (d, *J* = 15.9 Hz, 1 H), 3.10–3.05 (m, 2 H), 2.68 (m, 2 H), 2.24 (s, 3 H), 1.99–1.95 (m, 2 H), 1.75–1.84 (m, 2 H), 1.63–1.53 (m, 2 H), 1.43 (s, 9 H); ^13^C-NMR (400 MHz, Chloroform-d) δ 168.9, 167.1, 162.2, 158.9, 156.1, 147.2, 145.1, 129.2, 128.9, 121.4, 115.3, 107.3, 79.4, 61.2, 52.4, 47.4, 42.7, 40.5, 37.4, 32.3, 30.5, 28.4, 24.4, 23.0, 11.3; LC-MS: rt= 6.3 min, m/z: 600 [M + 1], 622 [M + 23].

**(19a)** Y = 20%. Anal. Calcd. C_27_H_36_N_6_O_7_ (556.26): C 58.19 H 6.51 N 15.10. ^1^H-NMR (400 MHz, Chloroform-d) δ 7.54 (s, 1 H), 7.02 (d, *J* = 8.4 Hz, 2 H), 6.92 (bs, 1 H), 6.58 (d, *J* = 8.4 Hz, 2 H), 5.39 (m, 1 H), 4.26 (d, *J* = 6.0 Hz, 2 H), 3.67 (s, 3 H), 3.58 (d, *J* = 16.4 Hz, 1 H), 3.50 (d, *J* = 16.4 Hz, 1 H), 2.81–2.70 (m, 2 H), 2.17 (s, 3 H), 1.81–1.51 (m, 6 H), 1.46 (s, 9 H); ^13^C-NMR (400 MHz, Chloroform-d) δ 173.9, 171.0, 169.1,167.3, 163.9, 147.1, 146.4, 134.0, 128.6, 122.2, 115.1, 106.3, 82.2, 60.8, 52.1, 51.6, 43.8, 33.4, 31.5, 27.9, 24.8, 24.3,11.8; LC-MS: rt= 6.8 min, m/z: 557 [M + 1], 1135 [2 M + 23].

**(19b)** Y = 34%. Anal. Calcd. C_28_H_38_N_6_O_7_ (570.28): C 58.92 H 6.72 N 14.73. ^1^H-NMR (400 MHz, Chloroform-d) δ 7.50 (s, 1 H), 7.02 (d, *J* = 8.4 Hz, 2 H), 7.29 (bs, 1 H), 6.65 (d, *J* = 8.4 Hz, 2 H), 5.47 (m, 1 H), 4.55 (bs, 1 H), 4.38–4.34 (m, 3 H), 4.18 (d, *J* = 14.4 Hz, 1 H), 3.67 (s, 3 H), 3.58 (d, *J* = 16.0 Hz, 1 H), 3.47 (d, *J* = 16.0 Hz, 1 H), 2.80 (t, *J* = 6.4 Hz, 2 H), 2.72 (m, 1 H), 2.39 (m, 1 H), 2.10 (s, 3 H), 1.78–1.54 (m, 4 H), 1.38 (s, 9 H); ^13^C-NMR (400 MHz, Chloroform-d) δ; 173.7, 170.9, 169.6, 167.6, 164.2, 147.3, 146.6, 133.7, 128.4, 122.0, 115.3, 106.1, 81.3, 60.6, 52.3, 48.8, 43.6, 35.4, 31.9, 30.4, 27.9, 24.6, 24.2,11.6. LC-MS: rt= 6.7 min, m/z: 571 [M + 1], 593 [M + 23].

**(23)** Y = 75%. Anal. Calcd. C_32_H_45_N_7_O_8_ (655.33): C 58.52 H 6.92 N 14.97. ^1^H-NMR (400 MHz, Chloroform-d) δ 7.67 (s, 1 H), 7.07 (m, 1 H), 7.00 (d, *J* = 8.4 Hz, 2 H), 6.58 (d, *J* = 8.4 Hz, 2 H), 5.39 (m, 1 H), 4.50 (m, 2 H), 4.26 (d, *J* = 5.6 Hz, 2 H), 3.67 (s, 3 H), 3.29 (m, 2 H), 3.16 (m, 2 H), 2.70–2.66 (m, 2 H), 2.53–2.48 (m, 2 H), 2.41–2.38 (m, 2 H), 2.17 (s, 3 H), 2.07–2.00 (m, 2 H), 1.83–1.57 (m, 4 H), 1.41 (s, 9 H); ^13^C-NMR (400 MHz, Chloroform-d) δ 172.0, 168.5, 166.8, 161.9, 158.5, 146.9, 145.0, 128.7, 128.5, 121.4, 114.8, 107.0, 80.4, 61.0, 60.0, 52.1, 47.1, 42.3, 40.2, 36.1, 32.0, 30.7, 30.3, 29.6, 27.7, 24.1, 13.8, 10.9; LC-MS: rt= 6.0 min, m/z: 656 [M + 1], 678 [M + 23].

**(27)** Y = 79%. Anal. Calcd. C_32_H_47_N_7_O_9_ (673.34): C 56.83 H 7.00 N 14.56. ^1^H-NMR (400 MHz, Chloroform-d) δ 7.53 (bs, 1 H), 7.45 (s, 1 H), 7.17 (d, *J* = 8.4 Hz, 2 H), 6.72 (d, *J* = 8.4 Hz, 2 H), 5.59 (m, 1 H), 4.95 (bs, 1 H), 4.47–4.45 (m, 4 H), 3.82 (t, *J* = 5.2 Hz, 2 H), 3.71 (s, 3 H), 3.61–3.46 (m, 6 H), 3.28–3.26 (m, 2 H), 3.08 (m, 1 H), 2.72 (m, 1 H), 2.27 (s, 3 H), 1.93–1.76 (m, 2 H), 1.63–1.54 (m, 2 H), 1.42 (s, 9 H), 1.27–1.22 (m, 2 H); ^13^C-NMR (400 MHz, Chloroform-d) δ 169.0, 167.0, 162.5, 162.2, 159.0, 155.9, 147.1, 145.2, 129.3, 129.1, 122.2, 115.2, 107.4, 70.5, 70.2, 70.0, 69.5, 61.2, 52.4, 50.1, 42.7, 40.6, 36.4, 32.3, 29.6, 28.4, 24.6, 11.3; LC-MS: rt= 6.4 min, m/z: 674 [M + 1], 696 [M + 23].

#### General procedure for the Huisgen reaction between acid (8) and azides: (26), (30)

In one-necked round bottom flask, in a mixture of tBuOH:H_2_O = 6:4 (4.5 ml) compound **8** (0.07 mmol), Cu(OAc)_2_ (2.5 mg, 0.014 mmol), Na-ascorbate (5.5 mg, 0.028 mmol) and azide (0.07 mmol) were added and the mixture was stirred at room temperature following the reaction by TLC. The mixture was filtered through celite and evaporated. The product was purified by flash chromatography (C18 reverse phase silica gel, 8/2 water/CH_3_CN).

**(26)** Y = 20%. Anal. Calcd. C_27_H_36_N_6_O_7_ (556.26): C 58.08 H 6.52 N 15.13. ^1^H-NMR (400 MHz, Methanol-d_4_) δ 7.43 (s, 1 H), 7.24 (bs, 1 H), 7.04 (d, *J* = 8.0 Hz, 2 H), 6.64 (d, *J* = 8.0 Hz, 2 H), 5.46 (m, 1 H), 4.85 (m, 2 H), 4.35–4.31 (m, 3 H), 4.18 (d, *J* = 15.0 Hz, 1 H), 4.08 (q, *J* = 7.6 Hz, 2 H), 3.19–3.14 (m, 2 H), 2.72–2.64 (m, 2 H), 2.32–2.30 (m, 2 H), 2.20–2.15 (m, 2 H), 2.13 (s, 3 H), 2.01–1.98 (m, 2 H), 1.87–1.78 (m, 2 H), 1.71–1.52 (m, 2 H), 1.28 (t, *J* = 7.6 Hz, 3 H); ^13^C-NMR (400 MHz, Methanol-d_4_) δ 173.5, 172.9, 171.8, 169.4, 164.2, 147.2, 146.6, 133.9, 128.3, 122.2, 115.4, 106.1, 61.4, 60.2, 49.2, 43.9, 36.4, 33.5, 30.5, 28.2, 24.5, 24.2, 22.0, 14.1, 10.2; LC-MS (acid eluents): rt= 9.0 min, m/z: 557 [M + 1], 579 [M + 23].

**(30)** Y = 40%. Anal. Calcd. C_28_H_38_N_6_O_9_ (602.27): C 55.98 H 6.34 N 13.94. ^1^H-NMR (400 MHz Methanol-d_4_) δ 7.82 (bs, 1 H), 7.27 (s, 1 H), 7.07 (d, *J* = 8.4 Hz, 2 H), 6.62 (d, *J* = 8.4 Hz, 2 H), 5.48 (m, 1 H), 4.86 (m, 2 H), 4.56–4.47 (m, 3 H), 4.40 (m, 1 H), 4.18 (q, *J* = 7.2 Hz, 2 H), 4.11–4.05 (m, 2 H), 3.88–3.85 (m, 2 H), 3.75–3.56 (m, 4 H), 2.70 (m, 1 H), 2.34 (m, 1 H), 2.20 (s, 3 H), 1.87–1.53 (m, 4 H), 1.26 (t, *J* = 7.2 Hz, 2 H)); ^13^C-NMR (400 MHz, Methanol-d_4_) δ 175.5, 172.6, 170.5, 170.0,168.5, 147.1, 146.4, 128.6, 128.1, 122.4, 115.3, 107.2, 71.8, 70.8, 70.3, 68.6, 61.6, 60.5, 49.8, 42.3, 39.7, 33.2, 29.3, 24.8, 11.3, 14.0. LC-MS: rt= 1.6 min, m/z: 603 [M + 1].

#### Synthesis of FITC-conjugated ligand (31)

In one-necked round bottom flask, to a solution of **16b** (12 mg, 0.02 mmol) in DCM (100 µl) trifluoroacetic acid (21 µl, 0.3 mmol) was added and the mixture stirred for 2 h. The solution was evaporated to obtain the desired intermediate as an orange oil. It was dissolved in DCM (300 µl) and TEA (11 µl, 0.08 mmol) was added dropwise. After 10 min, FITC (7 mg, 0.02 mmol) was added and the mixture stirred overnight at room temperature. The solvent was removed under vacuum and the crude used without further purifications. It was dissolved in a 2/1 mixture of THF/H_2_O (2 ml) and LiOH^.^H_2_O (0.4 mg, 0,02 mmol) was added to a solution of ester. The reaction was stopped after disappearance of the starting material, following the evolution by HPLC-MS. The solution was neutralized adding dropwise HCl 1 M solution and then water was evaporated. The crude was purified by preparative HPLC, affording 10 mg of the desired product. LC-MS: rt= 27.4 min, m/z: 875 [M + 1], 897 [M + 23].

### Pharmacology

#### Cell Culture

SK-MEL-24 (American Tissue Culture Collection, ATCC, Rockville, MD) were routinely grown in minimum essential medium (MEM, Cambrex, Walkersville, MD) supplemented with 10% fetal bovine serum (FBS), nonessential amino acids, and sodium pyruvate. K-562 (ATCC, Rockville, MD) were maintained as a stationary suspension culture in RPMI-1640 and L-glutamine with 10% FBS (Invitrogen (Carlsbad, CA). Cells were kept at 37 °C in a 5% CO_2_ humidified atmosphere. Forty hours before experiments, K-562 were treated with 25 ng/mL PMA (Sigma-Aldrich SRL, Milan, Italy) to induce differentiation and to increase expression of cell surface antigens^50^.

#### Cell adhesion assays

Plates (96 wells) (Corning Costar, Celbio, Milan,Italy) were coated by passive adsorption with fibronectin (10 µg/mL) or poly-L-lysine (0.002%) (Sigma-Aldrich SRL) overnight at 4 °C. Cells were counted with a haemocytometer and pre-incubated with different concentrations of each compound or with the vehicle (methanol) for 30 min at room temperature to reach a ligand-receptor equilibrium. Stock solutions (10^–2^ M) of the assayed compounds were prepared in phosphate-buffered saline (PBS). At the end of the incubation time, the cells were plated (50000 cells/well) and incubated at room temperature for 1 h. Then, all the wells were washed with PBS to remove nonadherent cells, and 50 µL of hexosaminidase [4-nitrophenylN-acetyl-β-D-glucosaminide dissolved at a concentration of 7.5 mM in 0.09 M citrate buffer solution (pH 5) and mixed with an equal volume of 0.5% Triton X-100 in water] was added. This product is a chromogenic substrate for β-N-acetylglucosaminidase that is transformed in 4-nitrophenol whose absorbance can be measured at 405 nm. As previously described^51^, there is a linear correlation between absorbance and enzymatic activity. Therefore, it is possible to identify the number of adherent cells among treated wells, interpolating the absorbance values of unknowns in a calibration curve. The reaction was blocked by addition of 100 µL of a stopping solution [50 µM glycine and 5 µM EDTA (pH 10.4)], and the plates were read in an EnSpire Multimode Plate Reader (PerkinElmer, Waltham, MA, USA). Experiments were carried out in quadruplicate and repeated at least three times. Data analysis and IC_50_/EC_50_ values were calculated using Graph-Pad Prism 5.0 (GraphPad Software, San Diego, CA).

#### Western blotting analysis

K562 cells were incubated in RPMI-1640 with 1% FBS for 16 h. Plates were coated with 10 µg/ml of fibronectin and blocked with 1% BSA (Sigma-Aldrich SRL). Subsequently, 4 × 106 cells were pre-incubated with different concentrations of compounds for 30 minutes. Cells were allowed to adhere for 1 hour on fibronectin in RPMI-1640 with 1% FBS. Cells treated with agonists were not incubated with fibronectin. Then, the cells were lysed in M-PER Mammalian Protein Extraction Reagent; (M-PER Pierce, Rockford, IL, USA) supplemented with phosphatase inhibitor (Sigma-Aldrich SRL) for 10 min at 4 °C by gently shaking. Cell debris were removed by centrifugation (14,000 × g for 15 minutes at 4 °C) and protein concentrations were estimated by BCA assay (Pierce, Rockford, IL, USA). Protein extracts (100 µg) were denatured at 95 °C for 3 min before being loaded and separated in 12% SDS–PAGE gels. The membranes were blocked in 1% BSA and incubated for 2 hours with anti-phospho-ERK 1/2 (extracellular signal-regulated kinase 1/2) (1:2500) or anti-total ERK 1/2 antibodies (1:5000) (Promega, Madison, WI, USA) and, thereafter with anti-rabbit peroxidase-conjugated secondary antibody. Digital images were acquired and analyzed following previously reported methods^52^.

#### Confocal laser scanning microscopy

HEK293 cells (not expressing α_5_ but endogenously expressing β_1_ integrin^47,48^ were plated in 6 well plates and at 50–60% confluence were transiently transfected with α_5_-EGFP plasmid using Lipofectamine2000 transfection reagent (LifeTechnologies). After 48 h from transfection, HEK293 + α_5_-EGFP cells were assessed to verify integrins expression by flow cytometry (data not shown). α_5_-EGFP integrin plasmid was a kind gift from Michael Davidson (Addgene plasmid #56423). HEK293 + α_5_-EGFP cells were treated at 4 °C with fibronectin (10 µg/ml) or the agonist **24** (1 µM) in MEM for 1 hour. Cells exposed to the antagonist **17b** (1 µM), before the addition of fibronectin, were pre-incubated with the compound for 20 minutes at 4 °C. After 1 h incubation with fibronectin or compound **24**, cells were moved to 37 °C for 15 minutes. Then, the cells were washed twice with PBS and fixed with paraformaldehyde (3% in PBS, pH 7.4, 10 min). Thereafter, the cover slips were washed twice with 0.1 M glycine in PBS and twice with 1% BSA (bovine serum albumin) in PBS.

In another set of experiments, to study the localization of FITC-conjugated integrin ligand **31**, K562 cancer cells or HEK293 cells transfected or not with α_5_ subunit coding plasmid (pCB7 alpha5) or with α_v_^53^ and β_3_ subunit coding plasmids were treated with compound **31** (0.1–1 μM) for 60 minutes, moved to 37 °C for 15 minutes and then fixed as previously described. Monensin (2 µM, 2 h) was employed as an inhibitor of integrin trafficking, and was added 1 h before compound **31** addition to K562 cells. As K562 were grown in suspension and they could not be grown attached to glass coverslip, for confocal microscopy experiments cells were treated and fixed as described above and then centrifuged using cytospin technique (1200 rpm 5 min; 1 ml/sample of 0.5 mil cells/ml solution). Nuclei were counterstained with 4’,6-diamidino-2-phenylindole dilactate (DAPI, Sigma). pCB7 alpha5 was a gift from Filippo Giancotti (Addgene plasmid #16041^54^). α_v_ integrin plasmid was a kind gift from Michael Davidson (Addgene plasmid no.57345), while β_3_ subunit coding plasmid was a kind gift of Prof. S.J.Shattil.

Specimens were embedded in Mowiol and analyzed using a Nikon C1s confocal laser-scanning microscope, equipped with a Nikon PlanApo 60×, 1.4-NA oil immersion lens. The images have elaborated using NIS-Elements C Software.

For internalization analysis of ligand **31**, the mean fluorescence intensity was related to the background; the relative fluorescence intensity is reported in the graph in Fig. 5.

### Computational studies

All calculations were performed using the Schrödinger Suite through the Maestro graphical interface [Maestro, version 10.5, Schrödinger, LLC, New York, NY, 2016].

#### Ligand preparation

Ionized carboxylate and neutral aniline are suggested by the Epik module [Epik version 3.5, Schrödinger, LLC, New York, NY, 2016] as the relevant protonation states at pH = 7 for the acid and basic pharmacophoric groups of isoxazoline derivatives according to predicted pK_a_ values of 3.3 (carboxylic acid) and 4.6 (phenyl anilinium derivative). These protonation states were considered for computational studies of the isoxazoline-containing compounds. Ionized carboxylate and protonated guanidinium groups have been employed for the cyclic RGD integrin ligands.

#### Protein preparation

The crystal structure of the extracellular domain of the integrin α_5_β_1_ in complex with the disulfide-bonded cyclic peptide ACRGDGWC (PDB code 4WK4) and the crystal structure of the extracellular domain of the integrin α_v_β_3_ in complex with the cyclic pentapeptide RGDf(NMe)V Cilengitide (PDB code 1L5G) were used for docking studies. The α_5_β_1_ integrin structure was set up for docking as previously reported (residues 40–351 for chain α_5_ and 121–358 for chain β_1_, Mg^2+^ ion at MIDAS)^55^. The α_v_β_3_ integrin structure was truncated to residue sequences 1–438 for chain α_v_ and 107–354 for chain β_3_, and all the bivalent cations were modeled as Mn^2+^ ions. Then, the Protein Preparation Wizard using the OPLS2005 force field was run to get the final structures.

#### Molecular docking

Docking calculations were performed using Glide version 7.0 [Glide version 7.0, Schrödinger, LLC, New York, NY, 2016] in the SP (Standard Precision) mode. Receptor grids were generated on the extracellular fragments of α_5_β_1_ and α_v_β_3_ integrin prepared as described in Protein Preparation. The settings of the docking step were defined as previously reported^32,52^. The GlideScore function was used to select 20 poses for each ligand after a post-minimization of the ligand structure within the binding site. The flexible-ligand docking method was selected and the SP mode was used with the option for amide bonds set to ‘Penalize non planar conformation’. No Epik state penalty was added to the docking score.

Each docking protocol was initially tested for its ability to reproduce the X-ray binding mode of the cyclic RGD ligand in the receptor crystal structure. Glide was successful in reproducing the experimentally determined binding mode of the cyclic peptide ACRGDGWC in α_5_β_1_ integrin and of Cilegitide in α_v_β_3_ integrin, as they correspond to the best-scored poses in the two docking runs.

#### Molecular dynamics simulations

Short MD simulations were run for selected derivatives to investigate the reliability of docking poses while allowing partial receptor flexibility. MD simulations were performed starting from the docking best poses and using MacroModel version 11.1 [MacroModel version 11.1, Schrödinger, LLC, New York, NY, 2016]. The following protocol was employed: OPLS2005 force field, implicit GB/SA water model, temperature 300 K, 1.0 fs integration step, 10 ps equilibration time and 10 ns simulation time. Variable degrees of freedom were assigned to the different moieties of the ligand-receptor complex, constraining the position of the atoms that are farthest from the receptor binding site and removing the interactions which are likely to have a negligible influence on the results. The ligand-integrin complex employed in the docking calculations is divided into four substructures according to the following scheme: the ligand is allowed to freely move, residues within 10 Å from the ligand are constrained at the crystal positions with a force constant K = 100 kJ mol^−1^ Å^−2^, residues within 5 Å from the second shell are constrained at the crystal positions with a force constant K = 200 kJ mol^−1^ Å^−2^, residues within 10 Å from the third shell are frozen in their respective crystal positions. The remaining residues are not considered in the calculation. Each structure has undergone a TNCG minimization step and, after the equilibration at 300 K, 2.000 structures from each simulation were saved for the analysis.

## Supplementary information

Supplementary Information.

## References

[1] Lammers T, Kiessling F, Hennink WE, Storm G (2012). Drug Target To Tumors: Principles, Pit-falls and (pre-) Clinical Progress. J. Controlled Release.

[2] Marelli UK, Rechenmacher F, Ali Sobahi TR, Mas-Moruno C, Kessler H (2013). Tumor Targeting Via Integrin Ligands. Front. Oncol..

[3] Liu Y, Miyoshi H, Nakamura M (2007). Nanomedicine For Drug Delivery And Imaging: A Promising Avenue For Cancer Therapy and Diagnosis Using Targeted Functional Nanoparticles. Int. J. Cancer.

[4] Srinivasarao M, Galliford CV, Low PS (2015). Principles In The Design Of Ligand-targeted Cancer Therapeutics And Imaging Agents. Nature Rev. Drug Disc..

[5] Desgrosellier JS, Cheresh DA (2010). Integrin In Cancer: Biological Implications And Therapeutic Opportunities. Nature Rev. Cancer.

[6] Ruoslathi. E (1996). RGD And Other Recognition Sequences For Integrins. Annu. Rev. Cell. Dev. Biol..

[7] Chen, K. & Chen, X. Integrin Targeted Delivery Of Chemotherapeutics. *Theranostics*, **1**, 189–200. PMID: 21547159; PMCID: PMC3086622 (2011). 10.7150/thno/v01p0189 PMC3086622 21547159

[8] Stupp R (2015). Cilengitide Combined With Standard Treatment For Patients With Newly Diagnosed Glioblastoma With Methylated MGMT Promoter (CENTRIC EORTC 26071-22072 study): A Multicenter, Randomized, open-label, phase 3 trial. Lancet Oncol.

[9] Danhier F, Le Breton A, Préat V (2012). RGD-based Strategies To Target Alpha(v) Beta(3) Integrin In Cancer Therapy And Diagnosis. Mol. Pharmaceutics.

[10] McEnaney PJ, Parker CG, Zhang AX, Spiegel DA (2012). Antibody-Recruiting Molecules: An Emerging Paradigm For Engaging Immune Function In Treating Human Disease. ACS Chem. Biol..

[11] Chari RVJ, Miller ML, Widdison WC (2014). Antibody–Drug Conjugates: An Emerging Concept In Cancer Therapy. Angew. Chem. Int. Ed..

[12] Dal Corso, A. *et al*. Synthesis And Biological Evaluation Of RGD Peptidomimetic–Paclitaxel Conjugates Bearing Lysosomally Cleavable Linkers. *Chem. Eur. J*., **21**, 6921 – 6929, 10.1002/chem.201500158 (2015). 10.1002/chem.201500158 25784522

[13] Dal Corso A, Pignataro L, Belvisi L, Gennari C (2016). α_v_β_3_ Integrin-Targeted Peptide/Peptidomimetic-Drug Conjugates: In-Depth Analysis Of The Linker Technology. Curr. Top. Med. Chem..

[14] D’souza AJM, Topp EM (2004). Release From Polymeric Prodrugs: Linkages And Their Degradation. J. Pharm. Sci..

[15] Pilkington-Miksa M (2012). Design, Synthesis, And Biological Evaluation Of Novel cRGD–Paclitaxel Conjugates For Integrin-Assisted Drug Delivery. Bioconjug. Chem..

[16] Alloatti D (2012). Camptothecins In Tumour Homing Via An RGD Sequence Mimetic. Bioorg. Med. Chem. Lett..

[17] Ganesh T (2007). Improved Biochemical Strategies For Targeted Delivery of Taxoids. Bioorg. Med. Chem..

[18] Tolomelli A (2011). Development Of Isoxazoline‐Containing Peptidomimetics As Dual αvβ3 And α5β1 Integrin Ligands. ChemMedChem.

[19] Benfatti F, Cardillo G, Gentilucci L, Mosconi E, Tolomelli A (2008). Lewis Acid Induced Highly Regioselective Synthesis Of A New Class Of Substituted Isoxazolidines. Synlett.

[20] Cruciani P, Stammler R, Aubert C, Malacria M (1996). New Cobalt-Catalyzed Cycloisomerization Of ε-Acetylenic β-Keto Esters. Application To A Powerful Cyclization Reactions Cascade. J. Org. Chem..

[21] Tolomelli A (2012). Exploring The Reactivity Of Alkylidene Malonamides: Synthesis Of Polyfunctionalized Isoxazolidinones, Aziridines and Oxazolines. ARKIVOC.

[22] Ferrazzano L (2016). New Isoxazolidinone And 3,4-dehydro-β-proline Derivatives As Antibacterial Agents And MAO-inhibitors: A Complex Balance Between Two Activities. *Eur*. J. Med. Chem..

[23] Cardillo G, Gentilucci L, Gianotti M, Perciaccante R, Tolomelli A (2001). Synthesis Of Aziridine-2,2-dicarboxylates Via 1,4-Addition Of N,O-(Bistrimethylsilyl)hydroxylamine To α,β-Unsaturated Malonates. J. Org. Chem..

[24] Ornelas C, Broichhagen J, Weck M (2010). Strain-Promoted Alkyne Azide Cycloaddition For The Functionalization Of Poly(amide)-Based Dendrons And Dendrimers. J. Am. Chem. Soc..

[25] Creary X, Anderso A, Brophy C, Crowell F, Funk Z (2012). Method For Assigning Structure Of 1,2,3-Triazoles. J. Org. Chem..

[26] Ndungu JM (2010). Targeted Delivery Of Paclitaxel To Tumor Cells: Synthesis And *In Vitro* Evaluation. J. Med. Chem..

[27] Jiang Y (2015). Design, Synthesis And Antifungal Activity Of Novel Paeonol Derivatives Linked With 1,2,3-Triazole Moiety By The Click Reaction. J. Chem. Res..

[28] Tu Y, Zhu V (2015). Enhancing Cancer Targeting And Anticancer Activity By A Stimulus-sensitive Multifunctional Polymer-drug conjugate. J. Contr. Release.

[29] Caltabiano S (1999). The Integrin Specificity Of Human Recombinant Osteopontin. Biochem. Pharmacol..

[30] Tolomelli, A. *et al*. Modulation Of αvβ_3_- And α_5_β_1_-integrin-mediated Adhesion By Dehydro-β-amino Acids Containing Peptidomimetics. *Eur. J. Med. Chem*., **66**, 258 – 268, 10.1016/j.ejmech.2013.05.050 (2013). g) Delouvrié, B. *et al*. Structure-Activity Relationship Of A Series Of Non Peptidic RGD Integrin Antagonists Targeting α5β1: Part 1. *Bioorg. Med. Chem. Lett*., **22**, 4111-6, 10.1016/j.bmcl.2012.04.063 (2012). 10.1016/j.ejmech.2013.05.050 23811088

[31] Galletti P (2014). Targeting Integrins α_v_β_3_ And α_5_β_1_ With New β-lactam Derivatives. *Eur*. J. Med. Chem..

[32] Baiula M (2016). New β-Lactam Derivatives Modulate Cell Adhesion And Signaling Mediated By RGD-Binding And Leukocyte Integrins. J. Med. Chem..

[33] Pisano M (2013). *In Vitro* Activity Of The α_v_β_3_ Integrin Antagonist RGDechi-hCit On Malignant Melanoma Cells. Anticancer Res..

[34] Sernissi L, Trabocchi A, Scarpi D, Bianchini F, Occhiato EG (2016). Cyclic RGD Peptidomimetics Containing 4- and 5-amino-cyclopropane Pipecolic Acid (CPA) Templates As Dual α_V_β_3_ And α_5_β_1_ Integrin Ligands. Bioorg. Med. Chem..

[35] Santulli RJ (2008). Studies With An Orally Bioavailable α_V_ Integrin Antagonist In Animal Models Of Ocular Vasculopathy: Retinal Neovascularization In Mice And Retinal Vascular Permeability In Diabetic Rats. J. Pharmacol. Exp. Ther..

[36] Fujii H (1995). Antimetastatic Activities Of Synthetic Arg-Gly-Asp-Ser (RGDS) And Arg-Leu-Asp-Ser (RLDS) Peptide Analogues And Their Inhibitory Mechanisms. Biol. Pharm. Bull..

[37] Baiula M (2016). New β-Lactam Derivatives Modulate Cell Adhesion and Signaling Mediated by RGD-Binding and Leukocyte Integrins. J. Med. Chem..

[38] Weis SM, Stupack DG, Cheresh DA (2009). Agonizing Integrin Antagonists?. Cancer Cell.

[39] Cox BD, Natarajan M, Gladson MR, Candece L (2006). New Concepts Regarding Focal Adhesion Kinase Promotion Of Cell Migration And Proliferation. J. Cell. Biochem..

[40] Schlaepfer DD, Hauck CR, Sieg DJ (1999). Signaling Through Focal Adhesion Kinase. Progr. Biophys. Mol. Biol..

[41] Benfatti F (2007). Synthesis and Biological Evaluation Of Non-peptide Alpha(v)Beta(3)/Alpha(5)Beta(1) Integrin Dual Antagonists Containing 5,6-dihydropyridin-2-one Scaffolds. Bioorg. Med. Chem..

[42] De Franceschi N, Hamidi H, Alanko J, Sahgal P, Ivaska J (2015). Integrin Traffic – The Update. J. Cell. Sci..

[43] Caswell PT, Norman JC (2006). Integrin Trafficking And The Control Of Cell Migration. Traffic.

[44] Pellinen T, Ivaska J (2006). Integrin Traffic. J. Cell Sci..

[45] Dozynkiewicz MA (2012). Rab25 And CLIC3 Collaborate To Promote Integrin Recycling From Late Endosomes/Lysosomes And Drive Cancer Progression. Dev. Cell.

[46] Lobert VH, Stenmark H (2012). The ESCRT Machinery Mediates Polarization Of Fibroblasts Through Regulation Of Myosin Light Chain. J. Cell Sci..

[47] Taherian, A., Li, X., Liu, Y. & Haas, T.A. a) Differences In Integrin Expression And Signaling Within Human Breast Cancer cells. *BMC Cancer*, **11**, 293–307, 10.1186/1471-2407-11-293 (2011). b) Qasem, A.R. *et al*. Contribution Of Alpha4beta1 Integrin To The Antiallergic Effect Of Levocabastine. *Biochem. Pharmacol*., 76, 751-762, 10.1016/j.bcp.2008.07.007 (2008). 10.1186/1471-2407-11-293 PMC3146943 21752268

[48] Qasem AR (2008). Contribution Of Alpha4beta1 Integrin To The Antiallergic Effect Of Levocabastine. Biochem. Pharmacol..

[49] Ahat E (2019). GRASP depletion-mediated Golgi destruction decreases cell adhesion and migration via the reduction of α5β1 integrin. Mol Biol Cell..

[50] Hunakova, L., Sedlak, J., Klobusicka, M., Sulikova, M. & Chorvath, B. Phorbol Ester (TPA)-induced Differential Modulation Of Cell Surface Antigens In Human Pluripotential Leukemia (K-562) Cell Line: Effects Of Protein Kinase Inhibitors With Broad- And PKC Selective Inhibitory Activity. *Neoplasma*, **42**, 249–253. PMID: 8552204 (1995). 8552204

[51] Shibaa H, Yagi T (1996). Rate Assay Of With 4-nitrophenyl As An Artificial Substrate. Clin. Chim. Acta.

[52] Bedini A, Baiula M, Spampinato S (2008). Transcriptional Activation Of Human Mu‐opioid Receptor Gene By Insulin‐like Growth Factor‐I In Neuronal Cells Is Modulated By The Transcription Factor REST. J. Neurochem..

[53] McKinney, S. A. *et al*. A bright and photostable photoconvertible fluorescent protein. *Nat. Methods*, **6**, 131−133, 10.1038/NMETH.1296 (2009). 10.1038/nmeth.1296 PMC2745648 19169260

[54] Giancotti FG, Ruoslahti E (1990). Elevated Levels Of The α_5_β_1_ Fibronectin Receptor Suppress The Transformed Phenotype Of Chinese Hamster Ovary Cells. Cell.

[55] Guzzetti I (2017). Insights Into The Binding Of Cyclic RGD Peptidomimetics To α5β1 Integrin By Live Cell NMR And Computational Studies. ChemistryOpen.

