# Supplementary material for: Side chain effect in the modulation of αvβ3/α5β1 integrin activity via clickable isoxazoline-RGD-mimetics: development of molecular delivery systems
Source: Sci Rep. 2020 May 4;10:7410. doi: 10.1038/s41598-020-64396-4 (PMC7198601; doi:10.1038/s41598-020-64396-4)
Supplement: Supplementary file 1 — Supplementary Information. [file 41598_2020_64396_MOESM1_ESM.docx]

**Supporting Information**

**Side chain effect in the modulation of α_v_β_3_/α_5_β_1_ integrin activity via clickable isoxazoline-RGD-mimetics: development of molecular delivery systems.**

Lucia Ferrazzano,*^[a]^ Dario Corbisiero,^[a]^ Eleonora Potenza,^[a]^ Monica Baiula,*^[b]^ Samantha Deianira Dattoli,^[b]^ Santi Spampinato,^[b]^ Laura Belvisi,^[c]^ Monica Civera^[c]^ and Alessandra Tolomelli^[a]^

*^[a]Department of Chemistry “G.Ciamician”, University of Bologna, Via Selmi 2, 40126, Bologna (Italy)^*

*^[b] Department of Pharmacy and Biothecnology, FABIT, University of Bologna, Via Irnerio 48, 40126, Bologna (Italy)^*

*^[c] Department of Chemistry, University of Milano, Via Golgi 19, 20133, Milano (Italy)^*

-) Representative ^1^H-NMR and ^13^C-NMR (pages 2-22)

-) Image of phosphorylation of ERK1/2 in K562 cells (page 23).

-) Docking poses of compound 17b in α_v_β_3_ and α_5_β_1_ pockets (page 24).

-) Results of calculation of physicochemical properties of the new compounds (page 25-35).


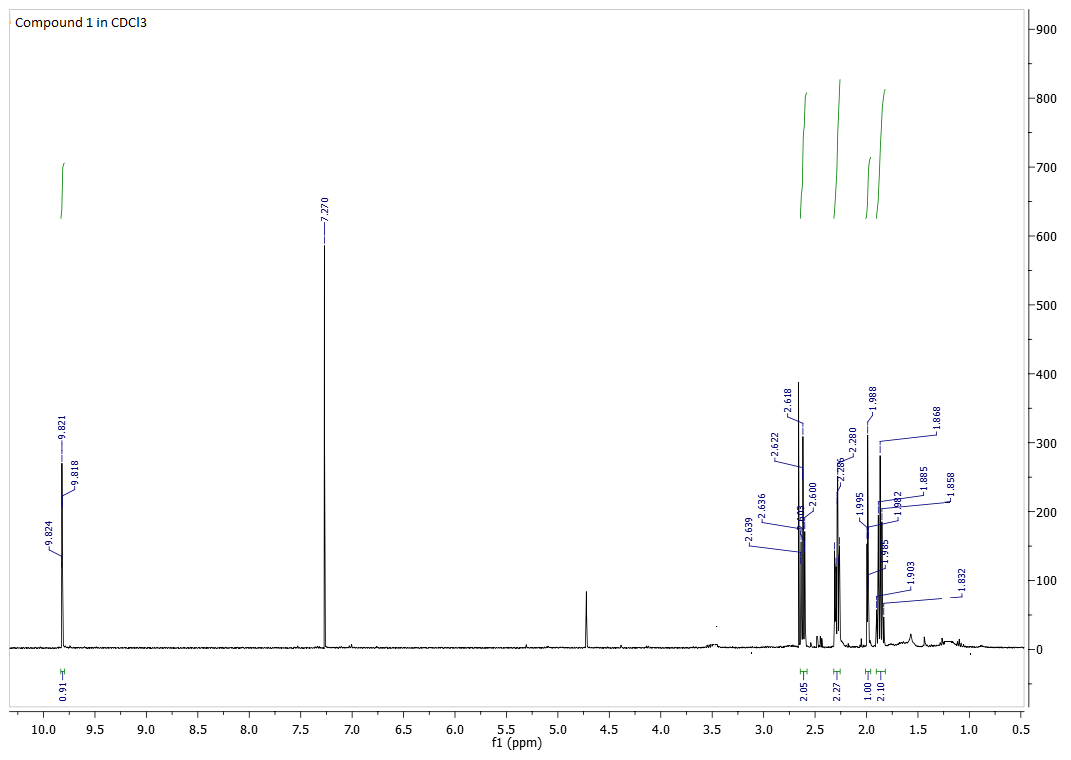


**Fig. S1** ^1^H-NMR spectrum of compound 1.


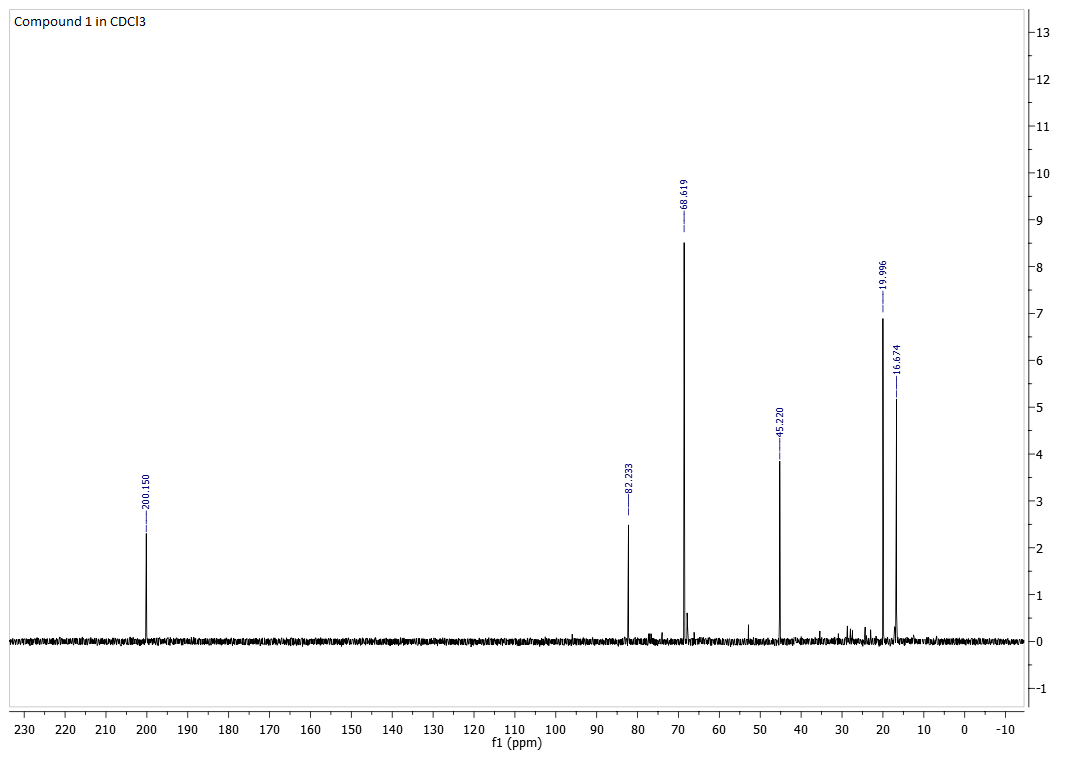


**Fig. S2** ^13^C-NMR spectrum of compound 1.


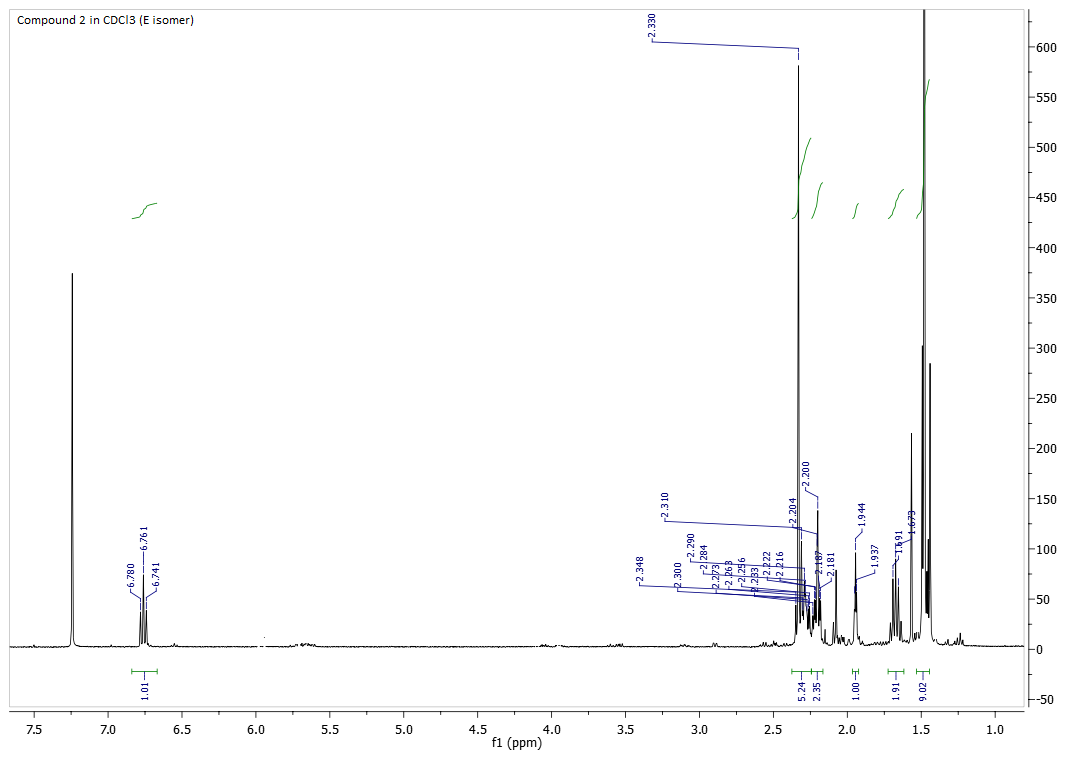


**Fig. S3** ^1^H-NMR spectrum of compound 2 (E isomer).


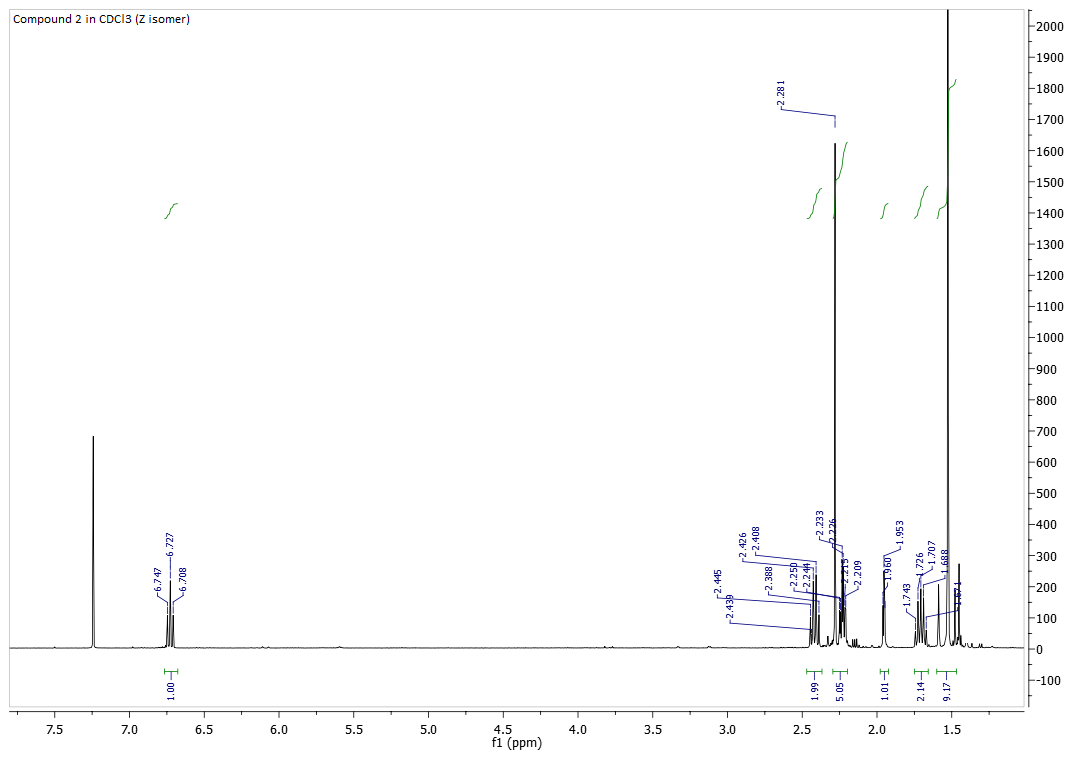


**Fig. S4** ^1^H-NMR spectrum of compound 2 (Z isomer).


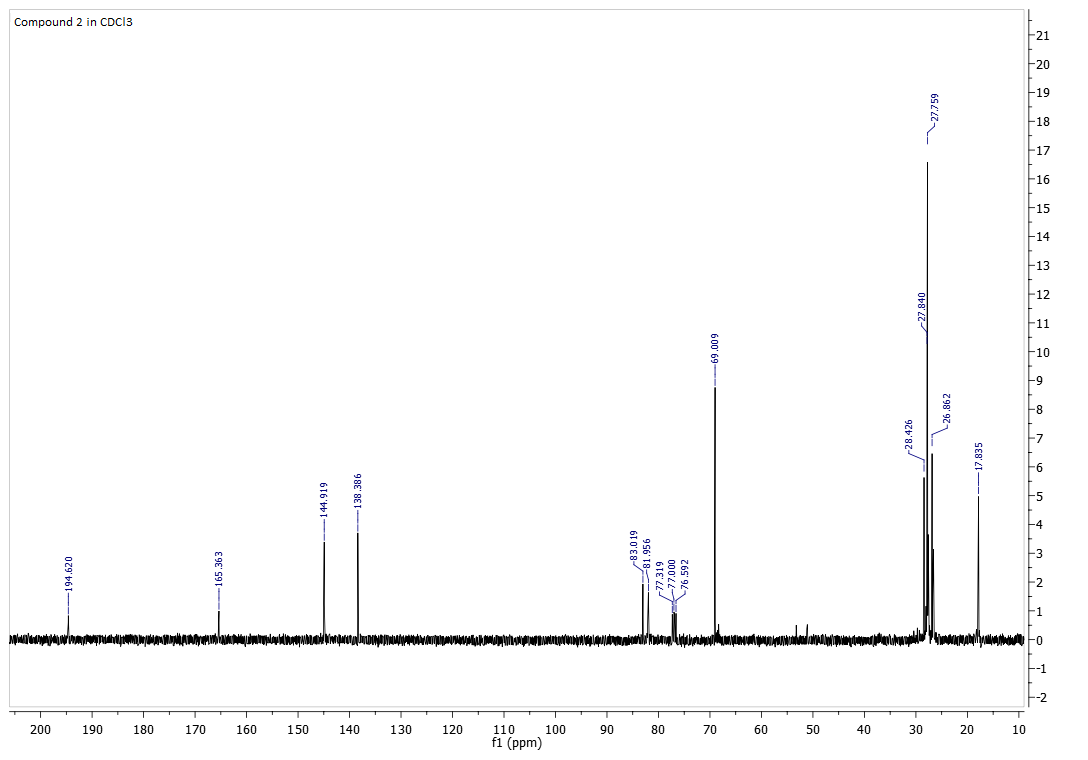


**Fig. S5** ^13^C-NMR spectrum of compound 2.


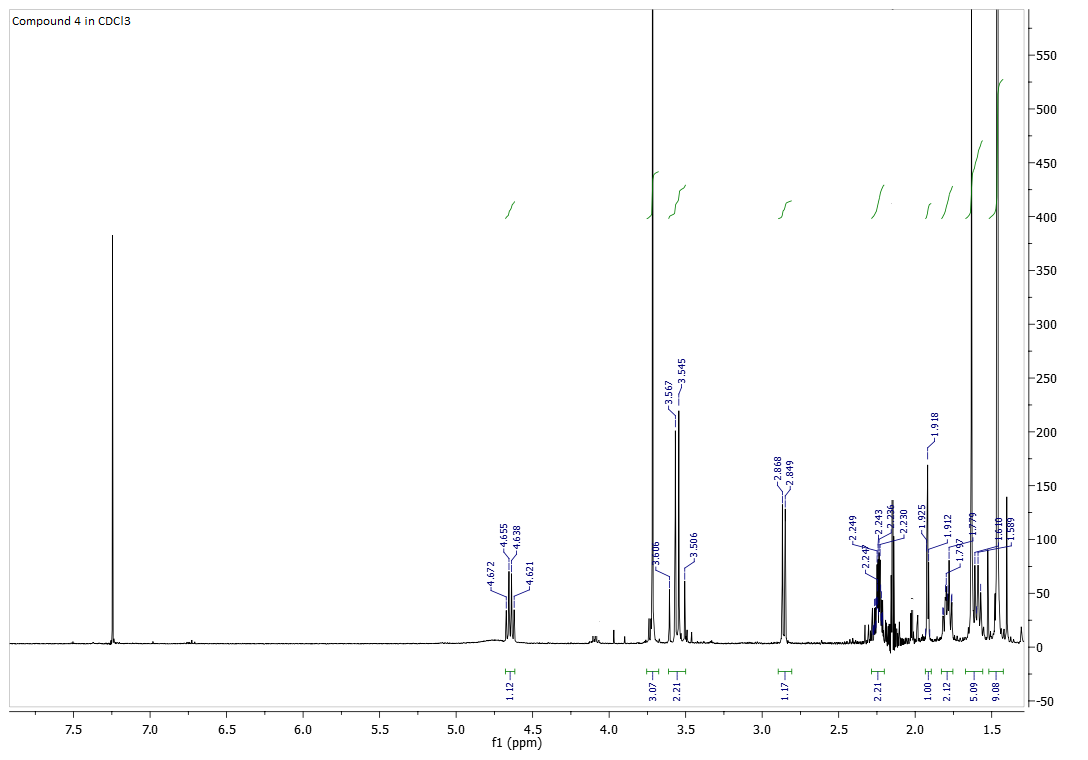


**Fig. S6** ^1^H-NMR spectrum of compound 4.


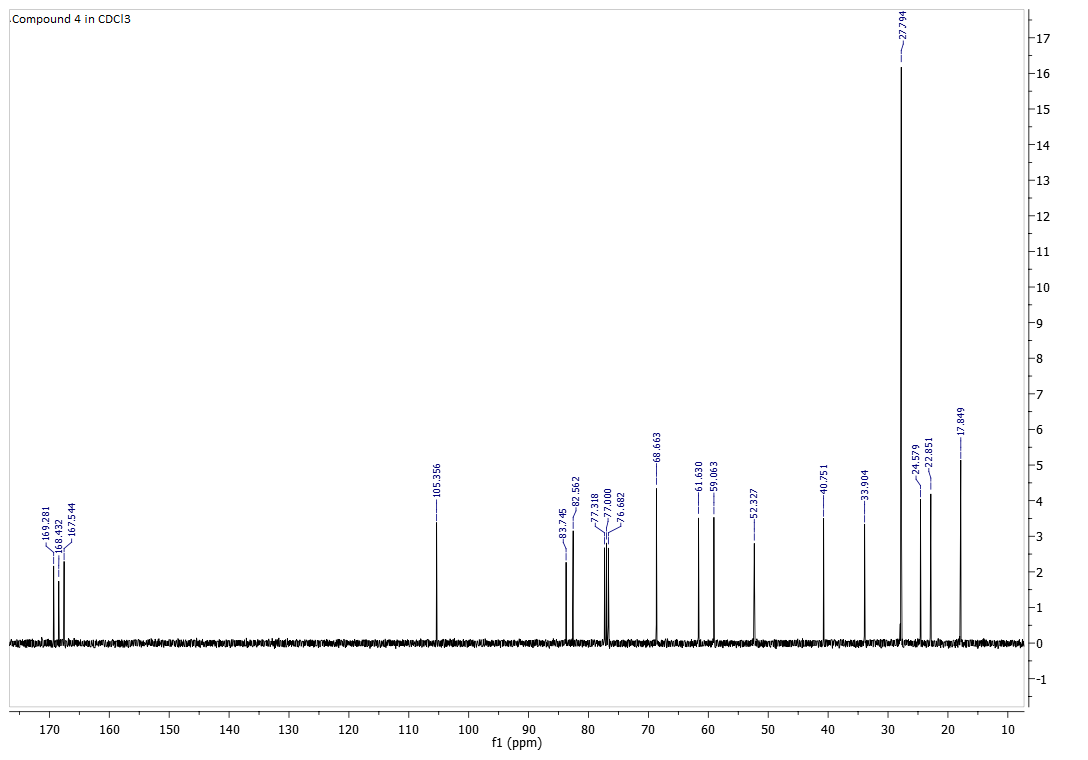


**Fig. S7** ^13^C-NMR spectrum of compound 4.


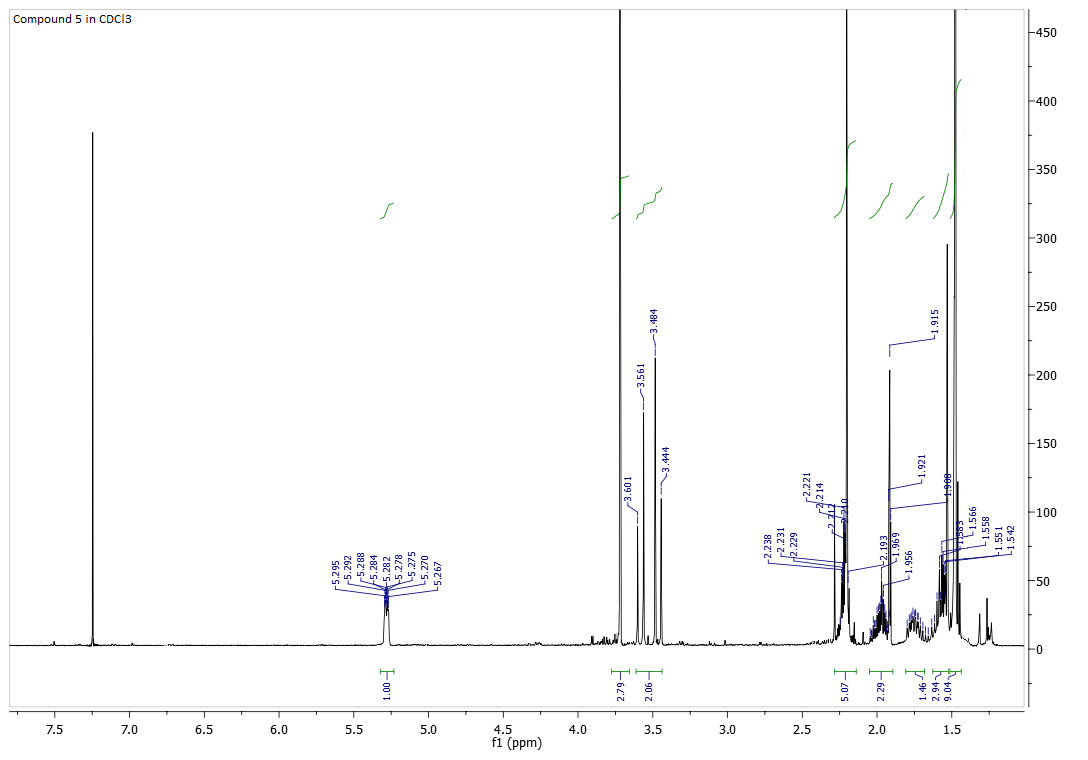


**Fig. S8** ^1^H-NMR spectrum of compound 5.


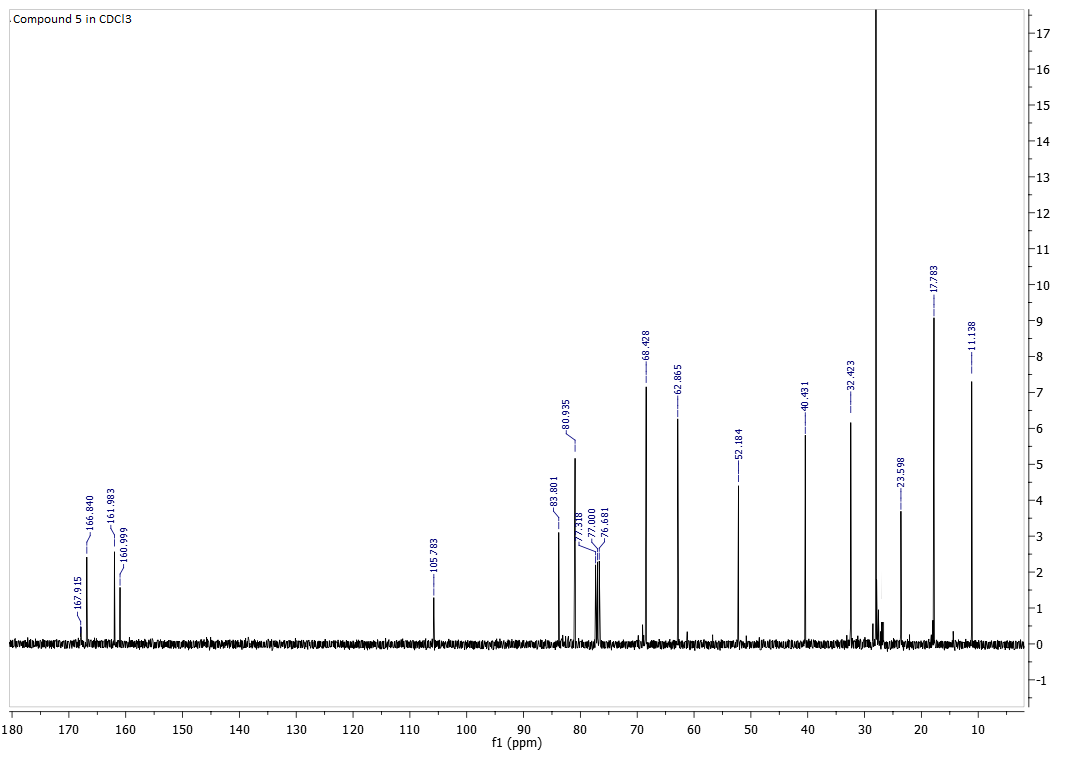


**Fig. S9** ^13^C-NMR spectrum of compound 5.


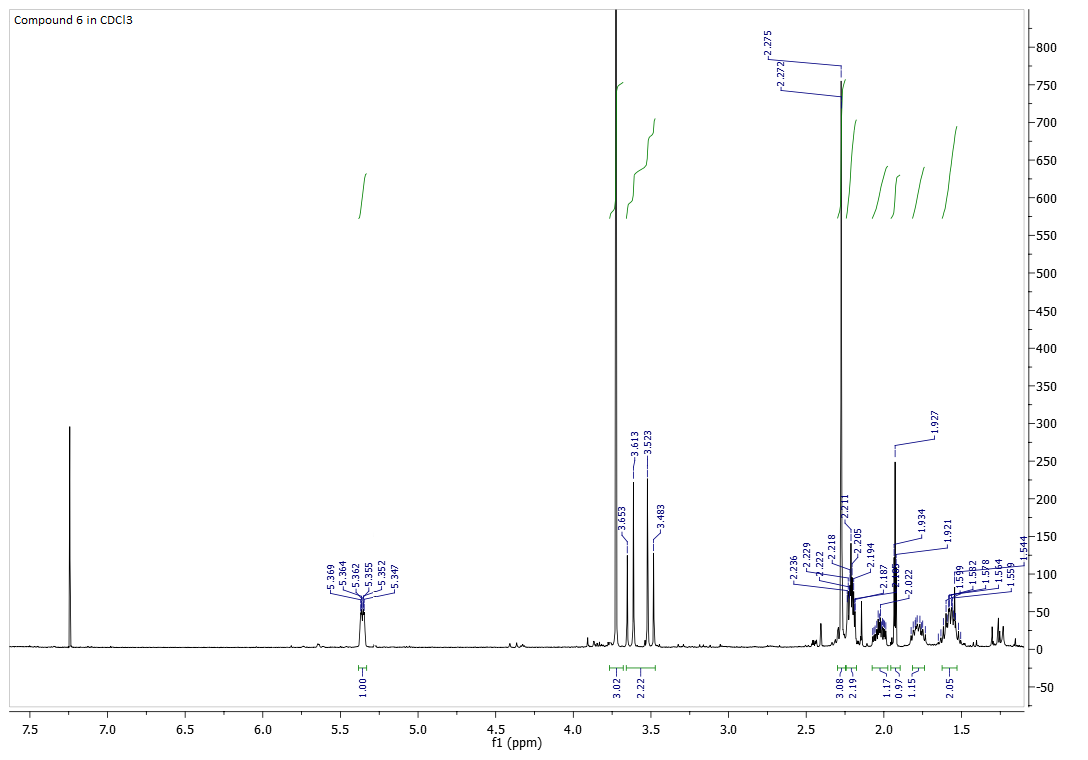


**Fig. S10** ^1^H-NMR spectrum of compound 6.


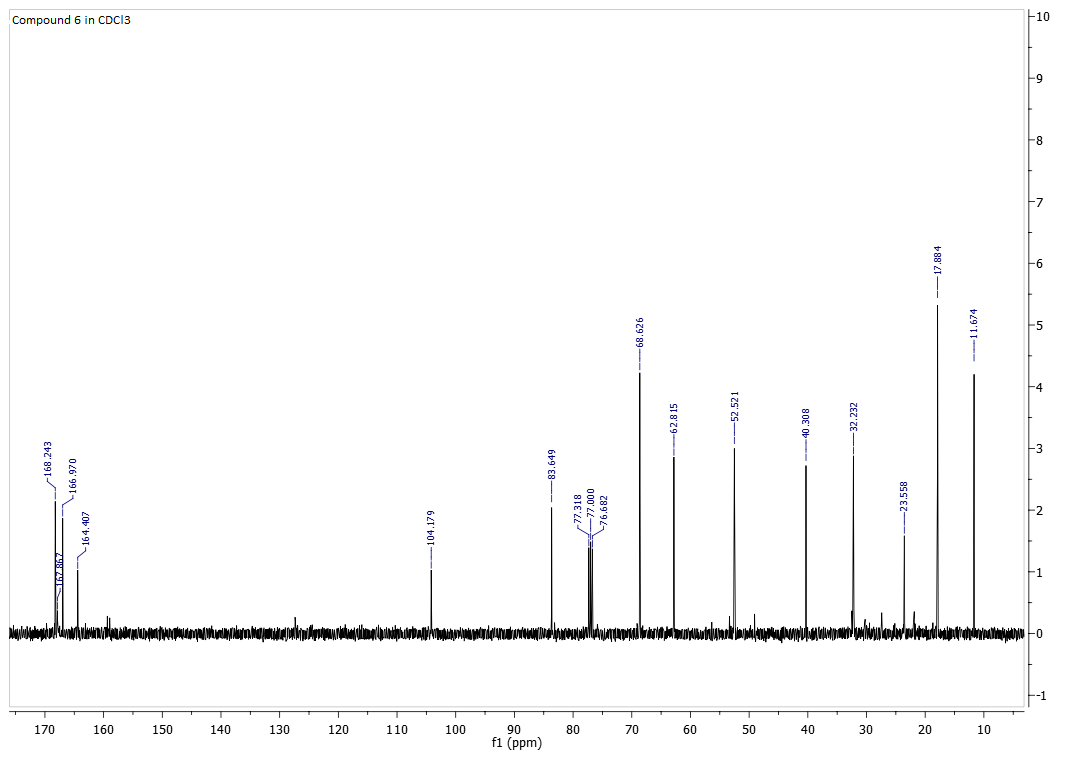


**Fig. S11** ^13^C-NMR spectrum of compound 6.


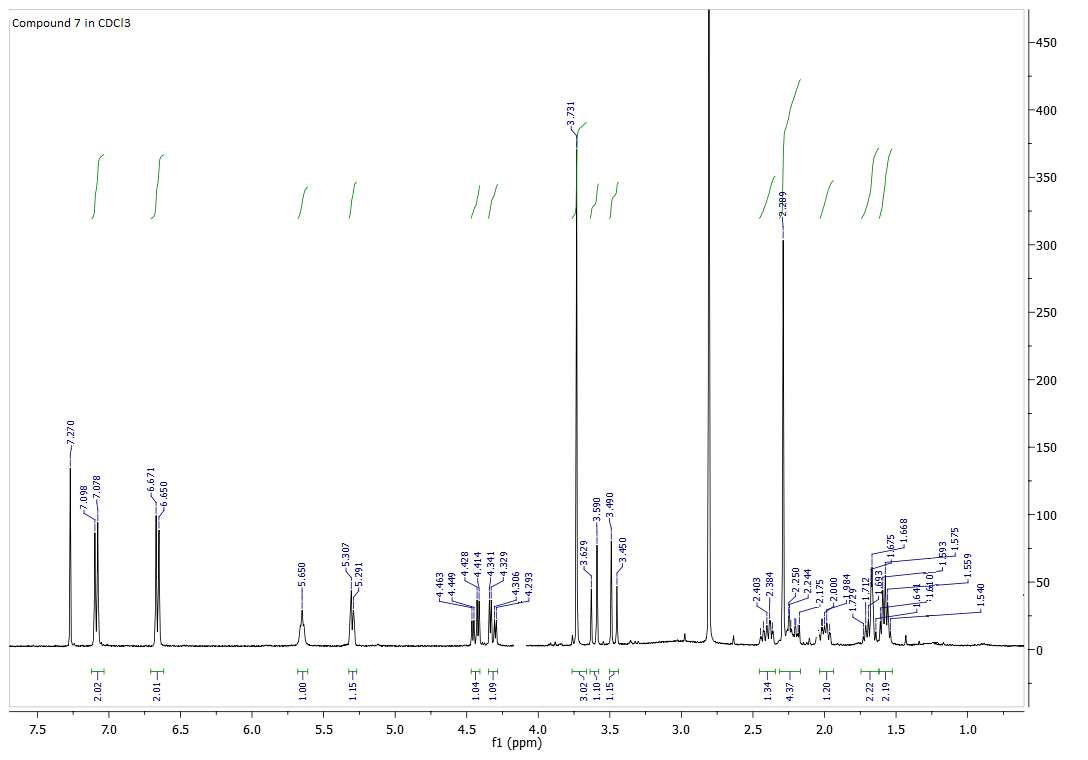


**Fig. S12** ^1^H-NMR spectrum of compound 7.


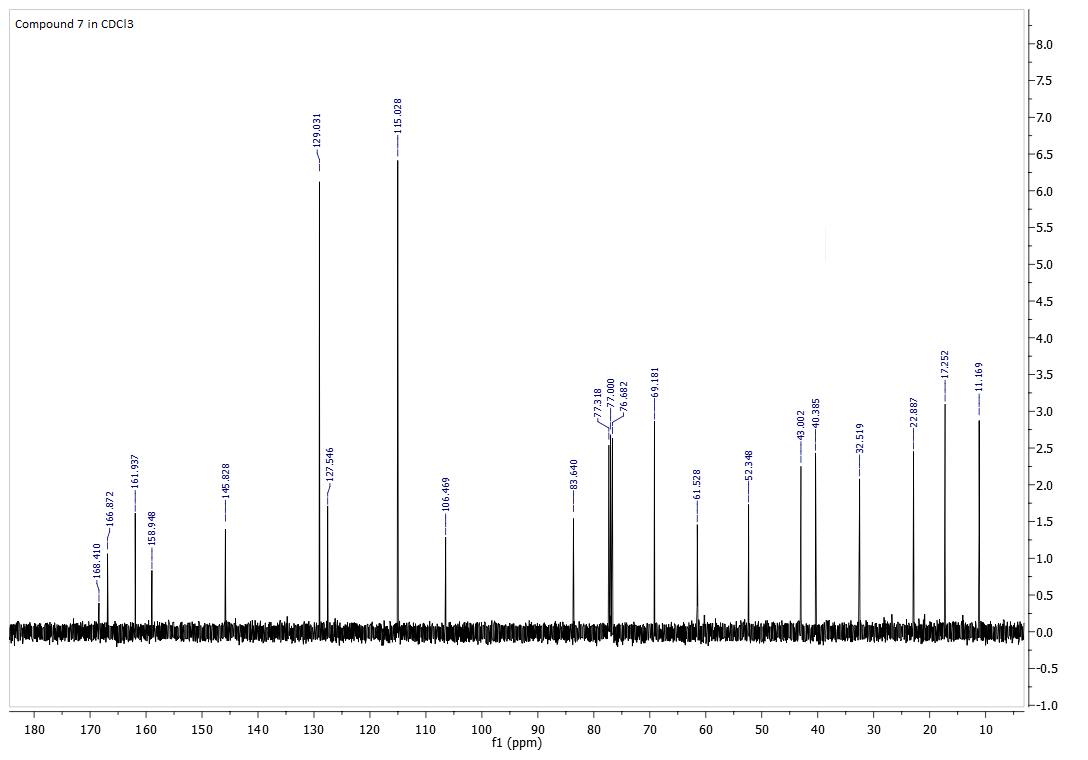


**Fig. S13** ^13^C-NMR spectrum of compound 7.


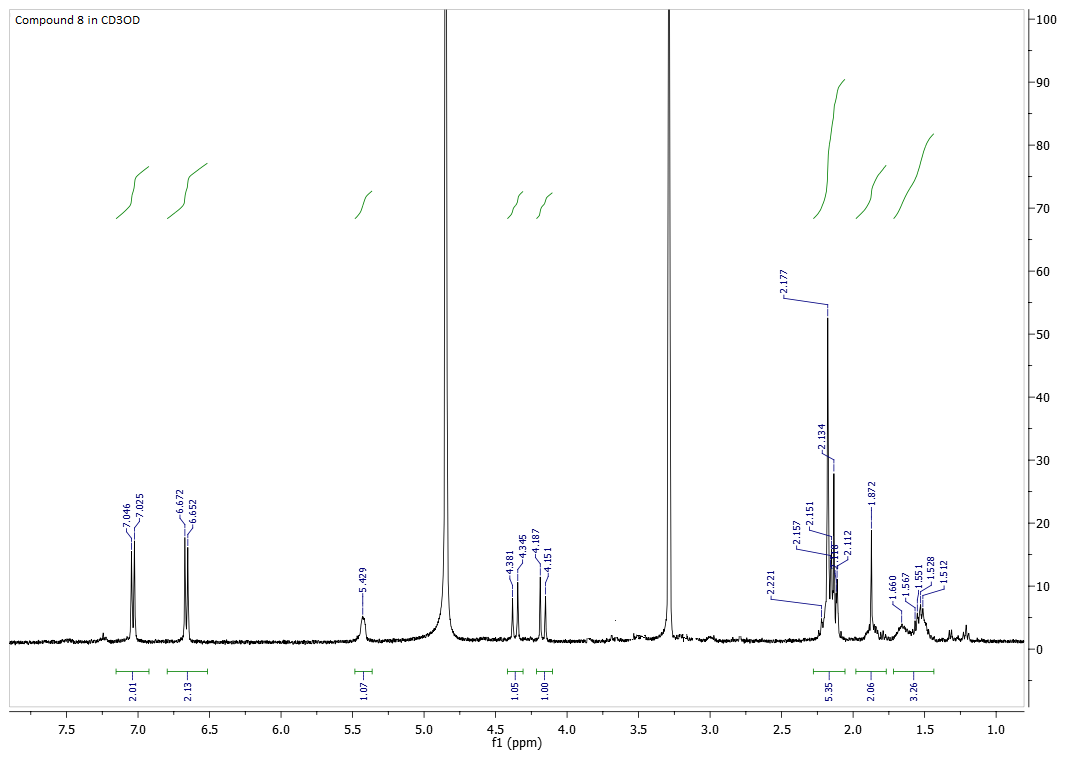


**Fig. S14** ^1^H-NMR spectrum of compound 8.


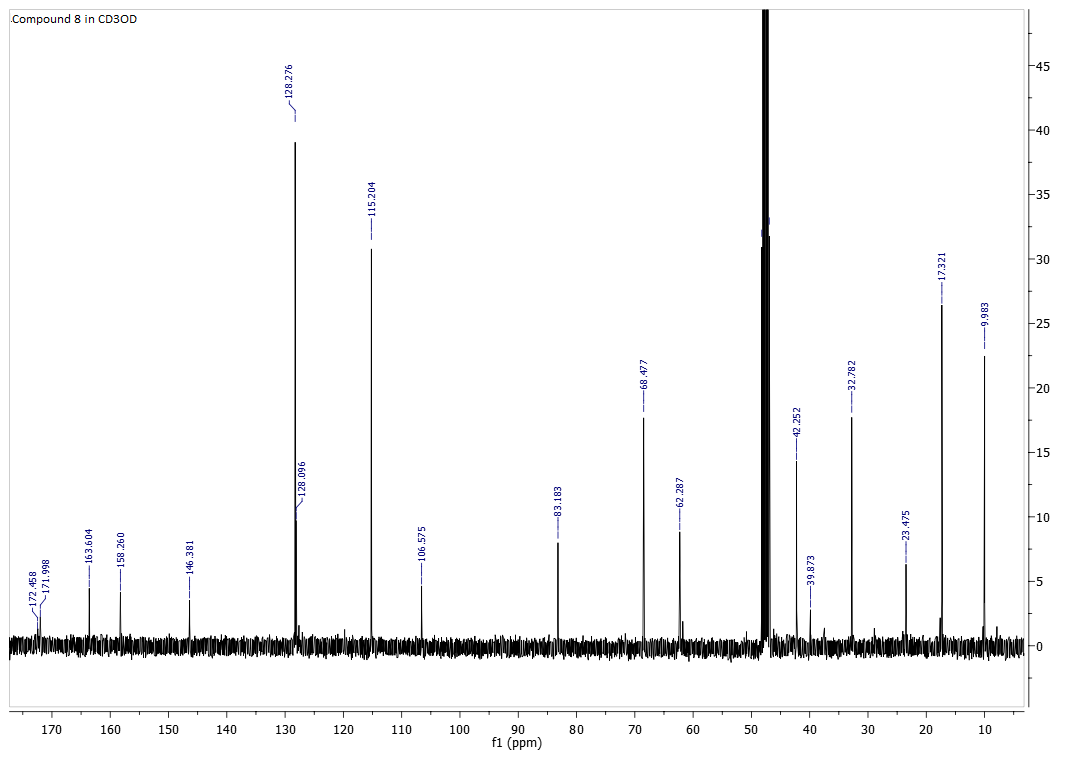


**Fig. S15** ^13^C-NMR spectrum of compound 8.


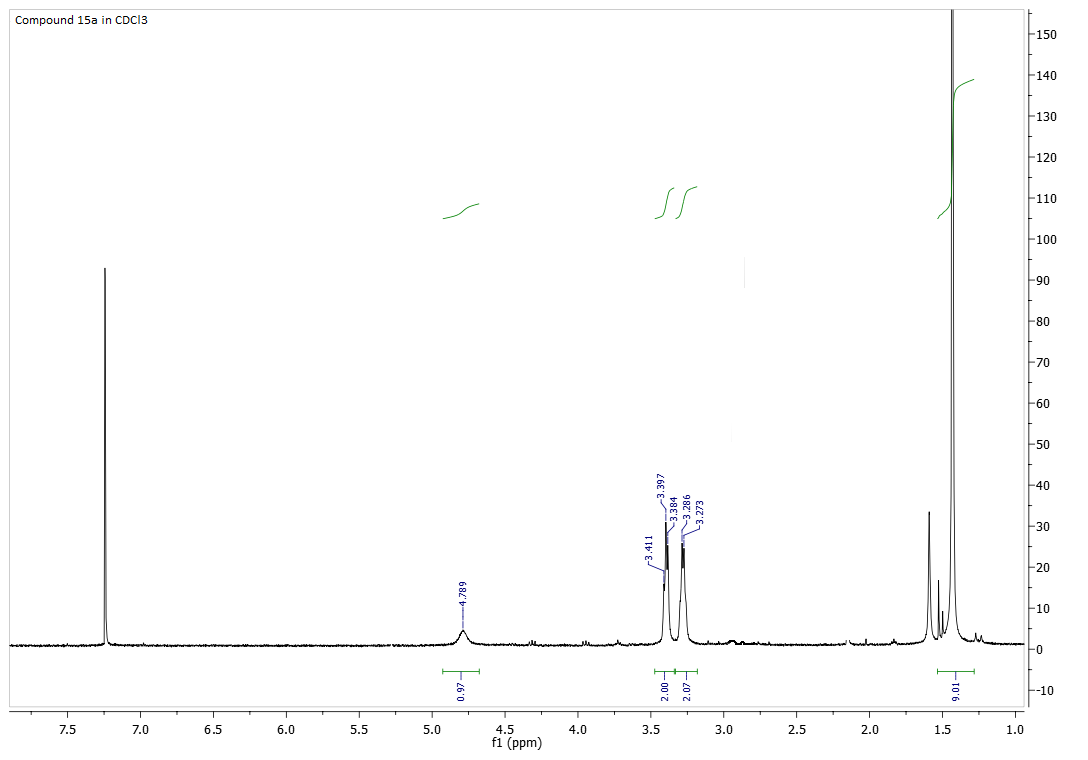


**Fig. S16** ^1^H-NMR spectrum of compound 15a.


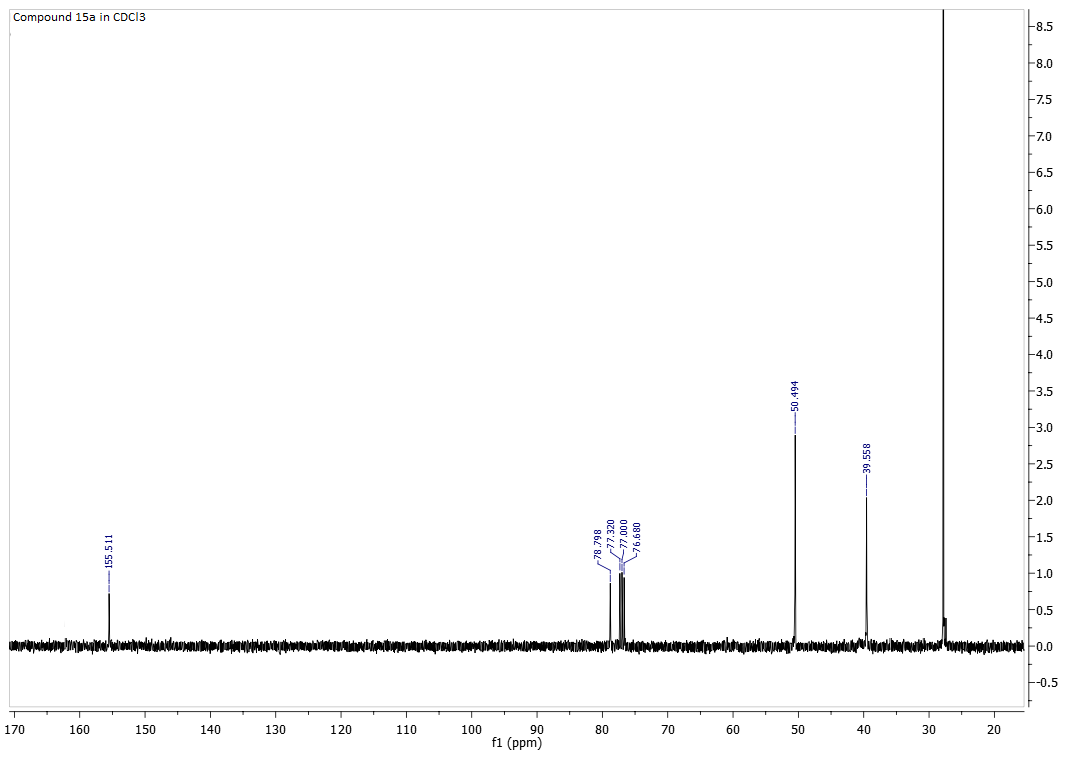


**Fig. S17** ^13^C-NMR spectrum of compound 15a.
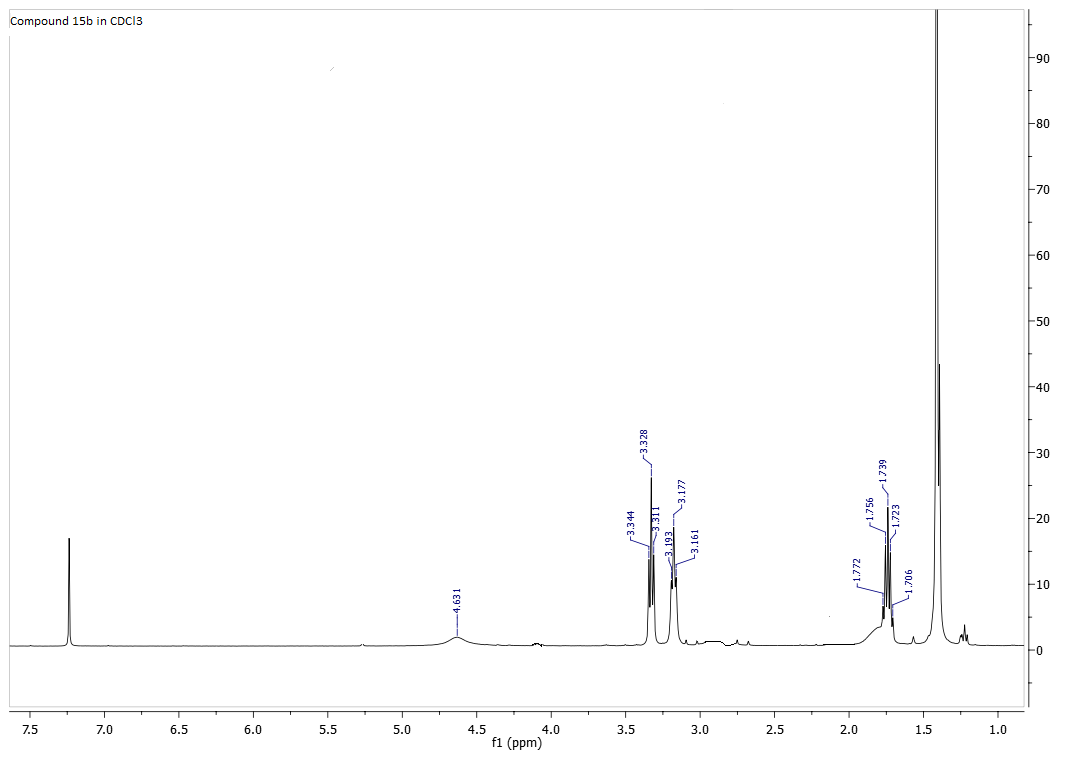


**Fig. S18** ^1^H-NMR spectrum of compound 15b.


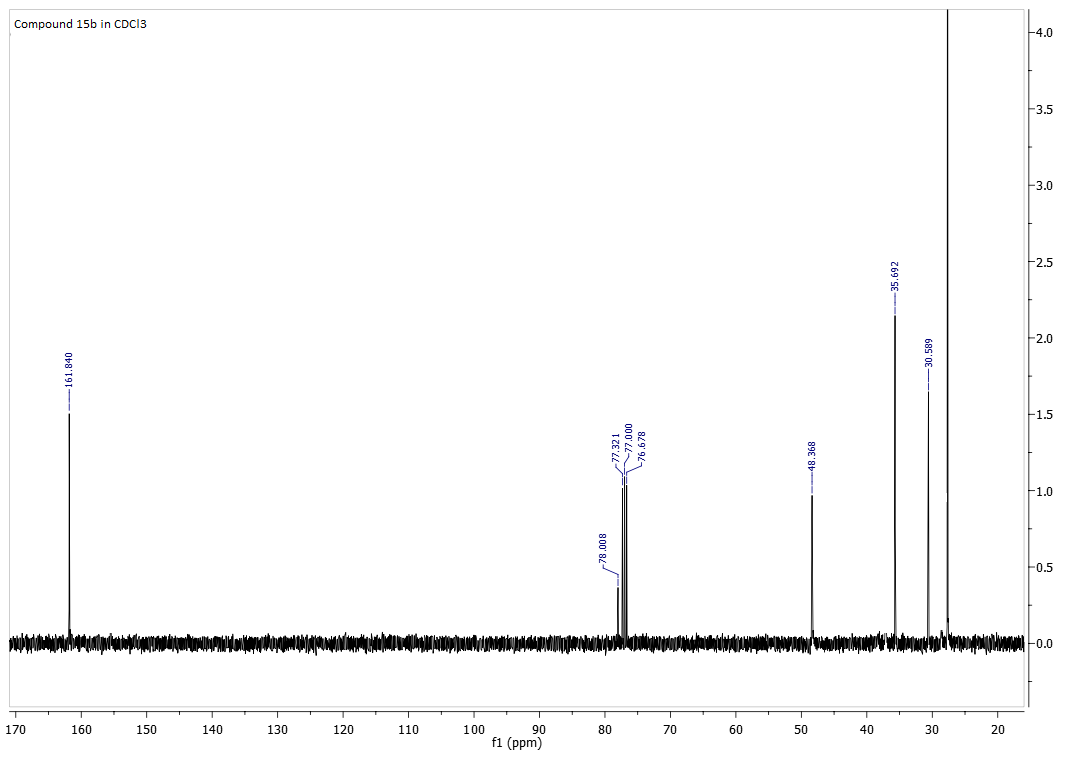


**Fig. S19** ^13^C-NMR spectrum of compound 15b.


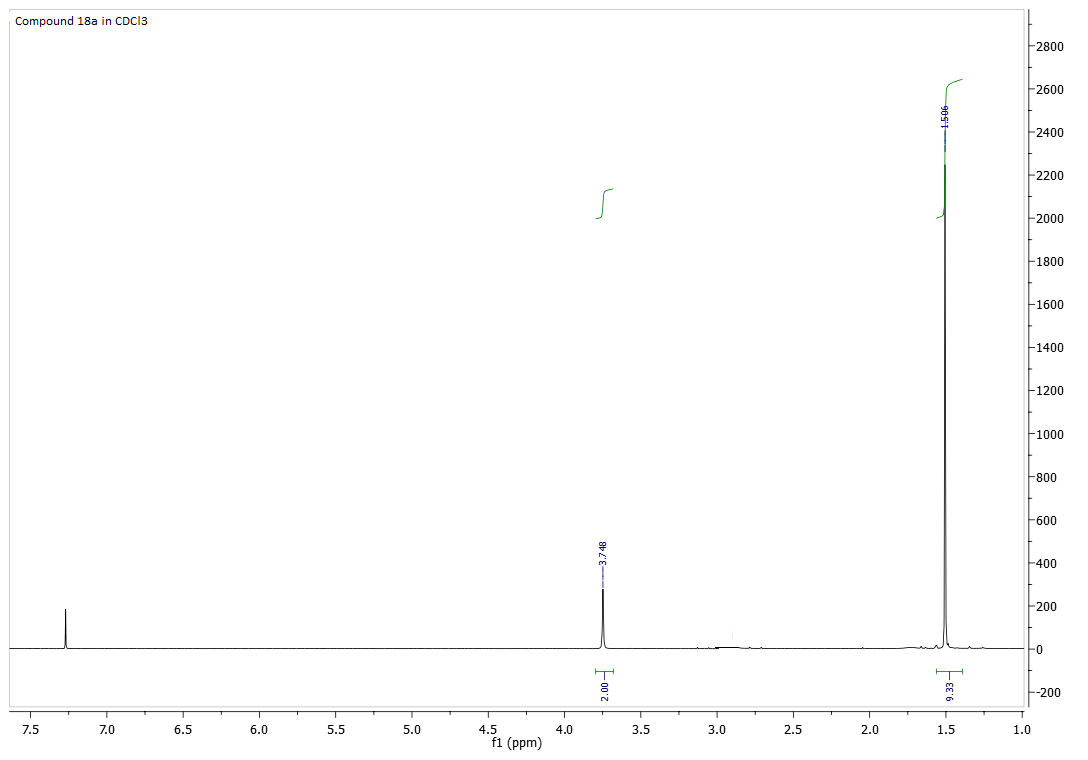


**Fig. S20** ^1^H-NMR spectrum of compound 18a.


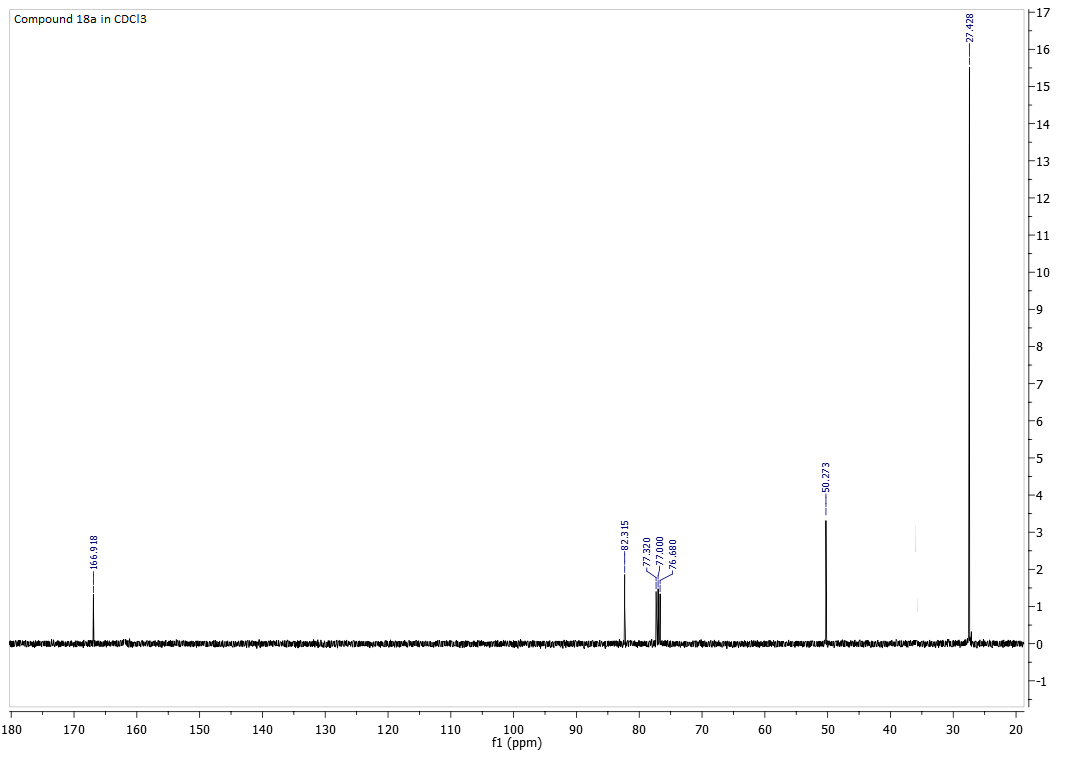


**Fig. S21** ^13^C-NMR spectrum of compound 18a.


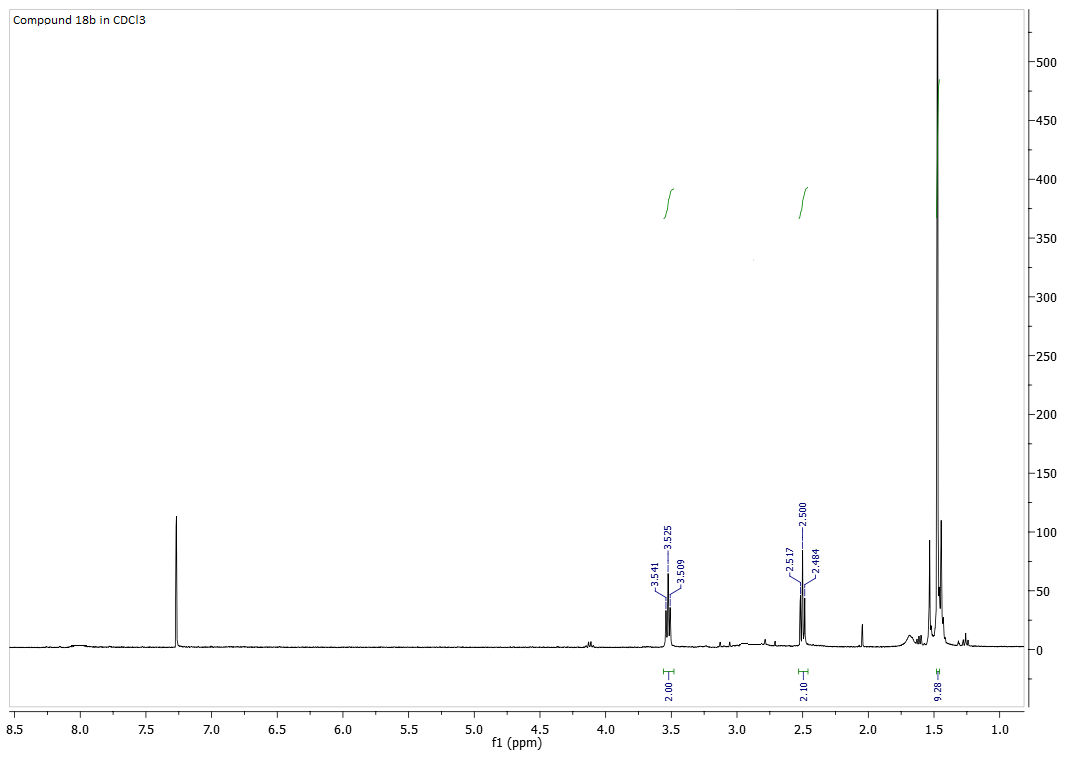


**Fig. S22** ^1^H-NMR spectrum of compound 18b.


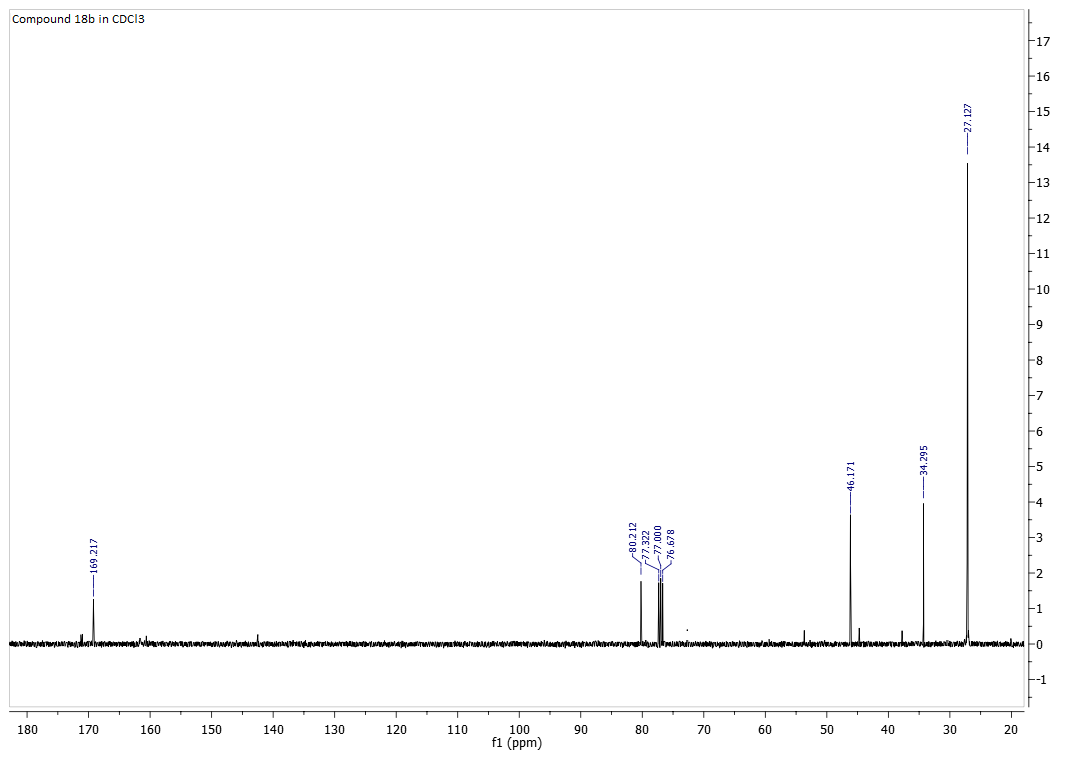


**Fig. S23** ^13^C-NMR spectrum of compound 18b.


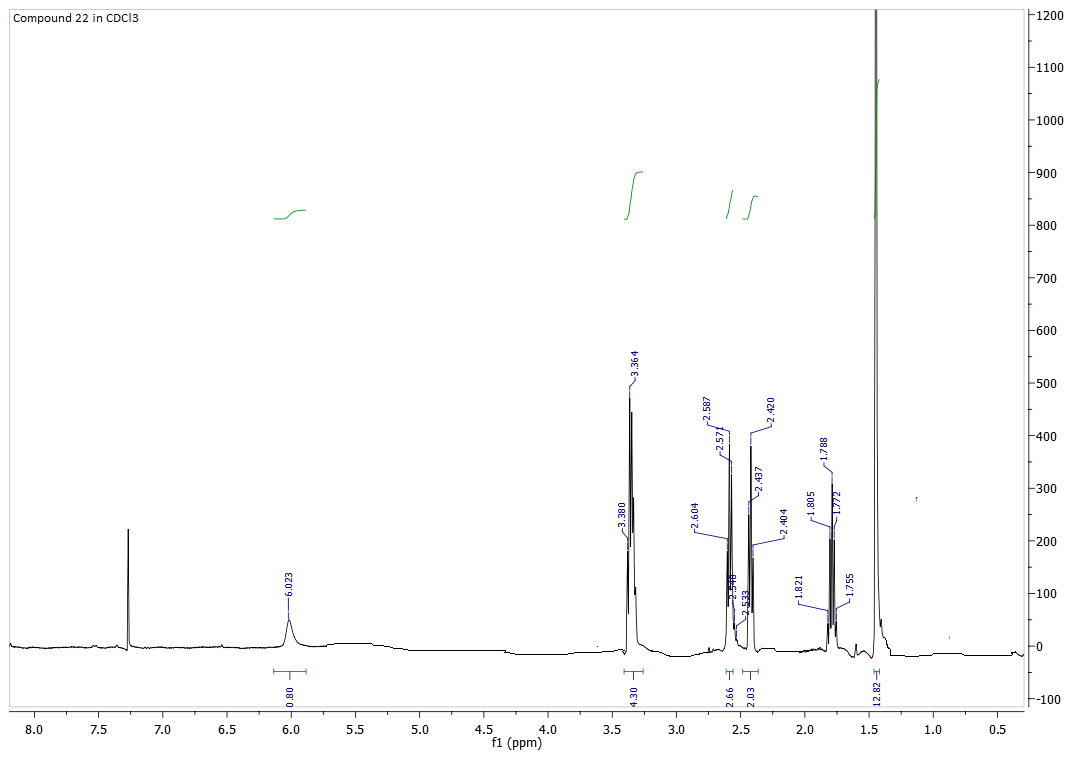


**Fig. S24** ^1^H-NMR spectrum of compound 22.


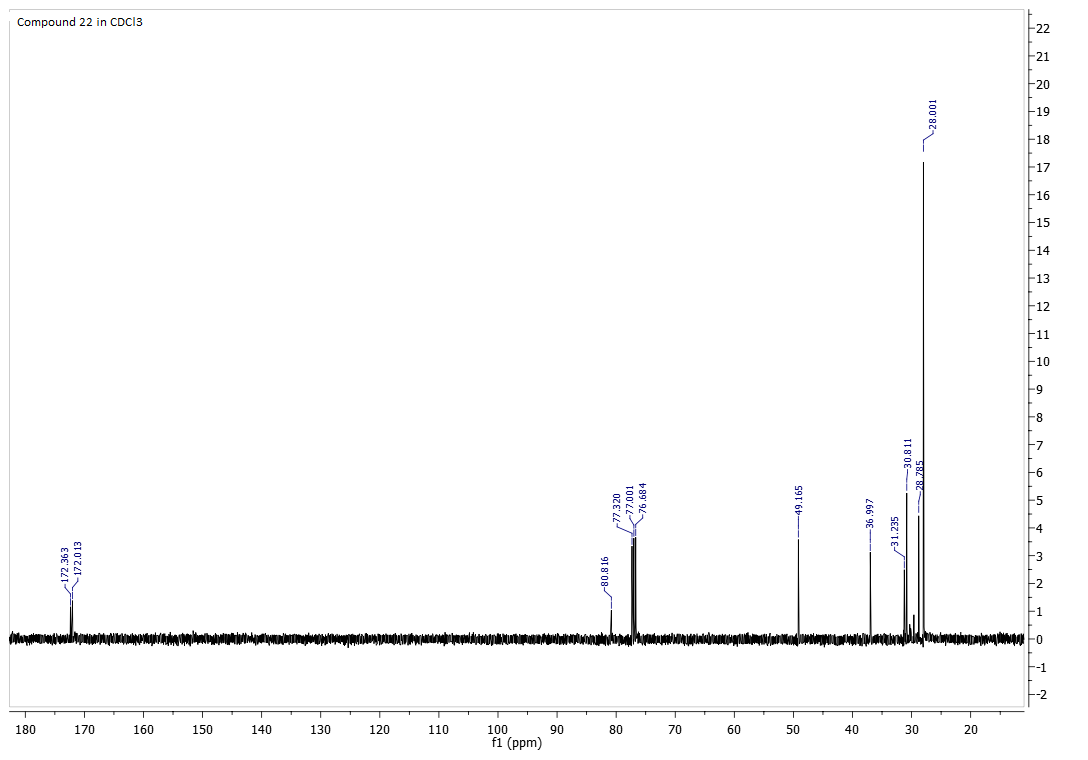


**Fig. S25** ^13^C-NMR spectrum of compound 22.


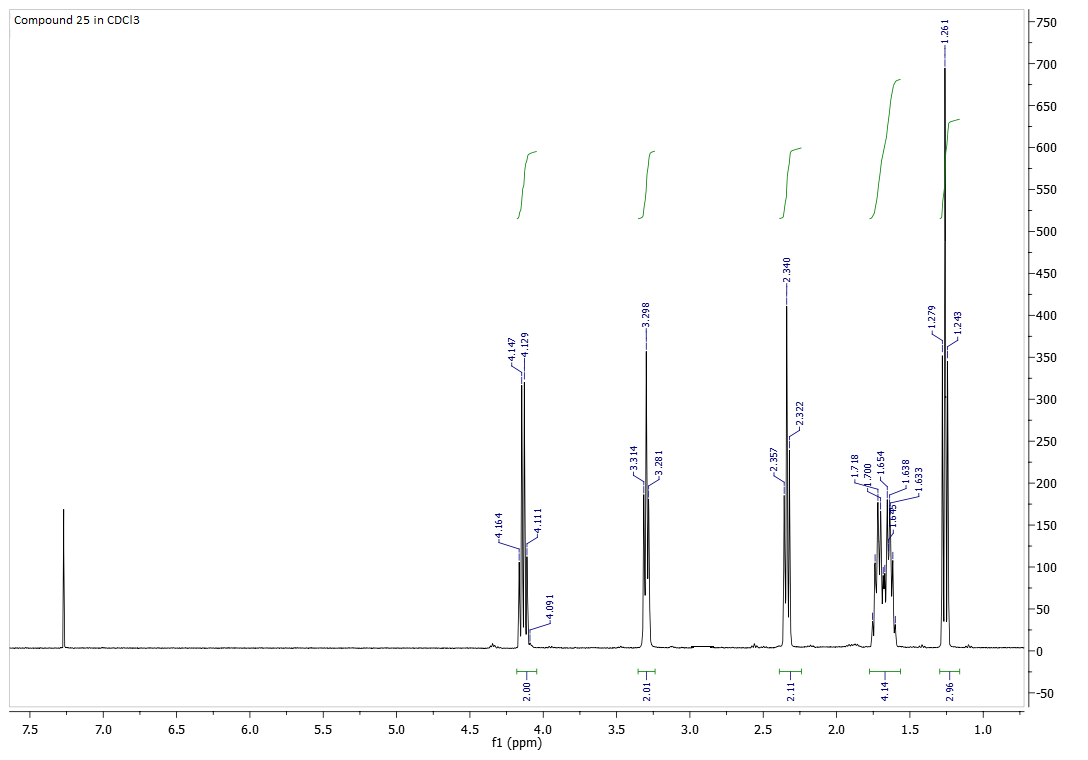


**Fig. S26** ^1^H-NMR spectrum of compound 25.


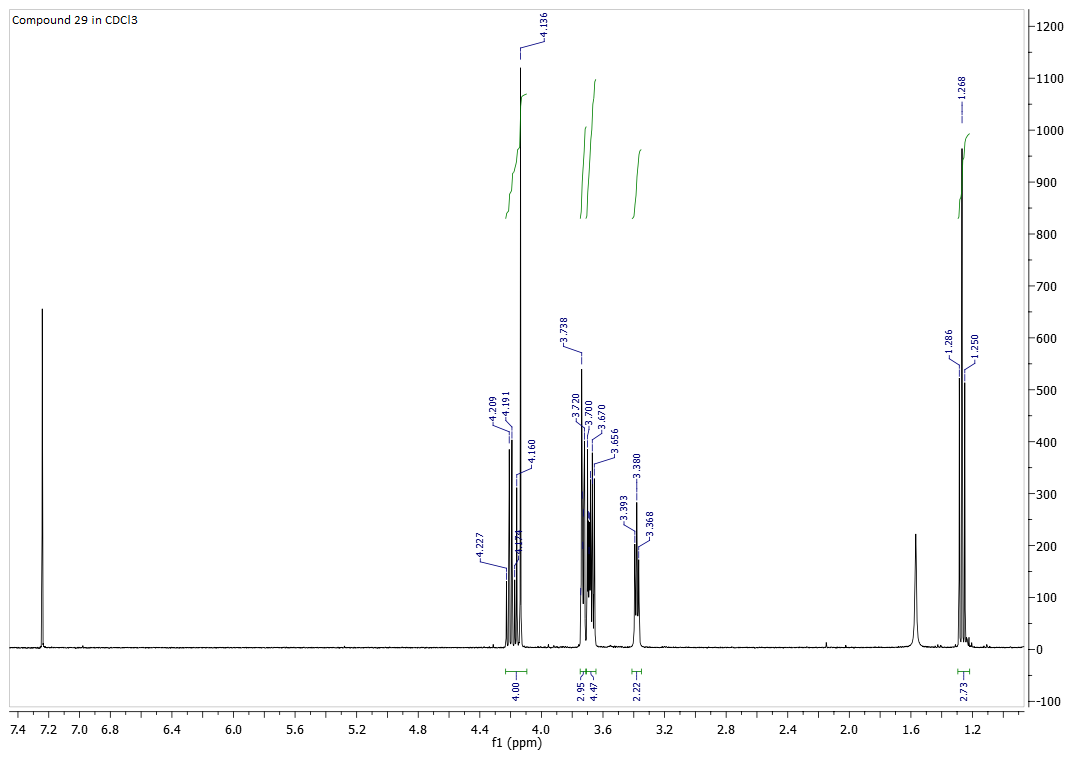


**Fig. S27** ^1^H-NMR spectrum of compound 29.


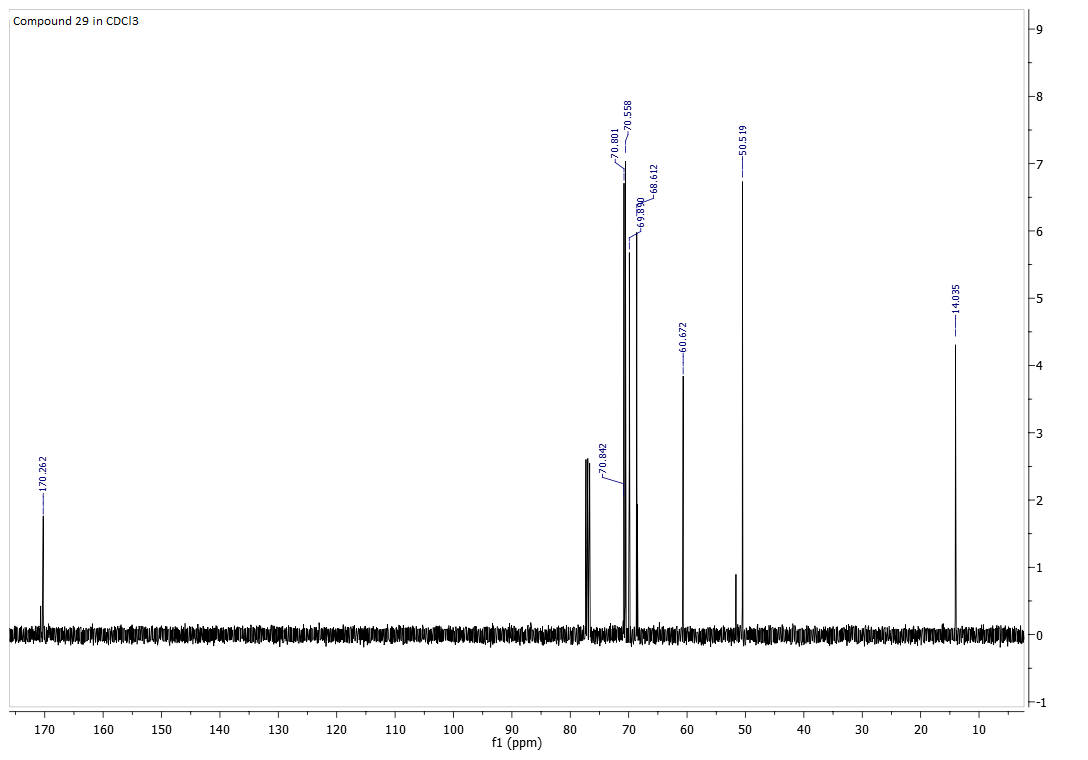


**Fig. S28** ^13^C-NMR spectrum of compound 29.


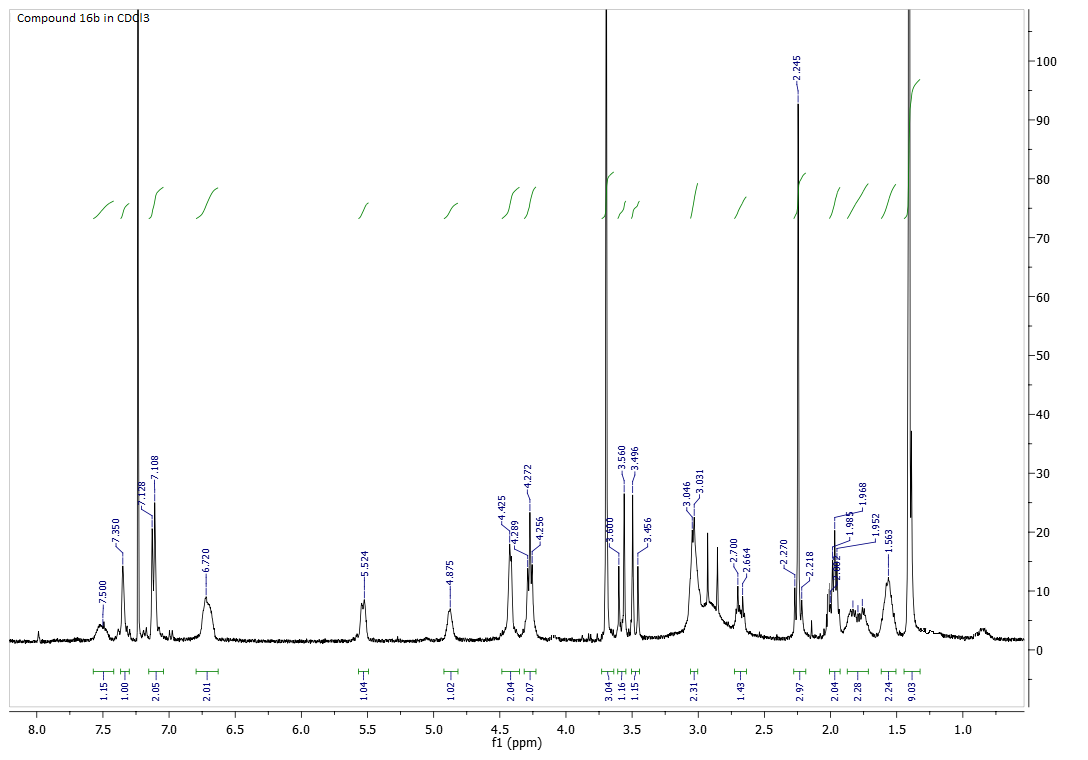


**Fig. S29** ^1^H-NMR spectrum of compound 16b.


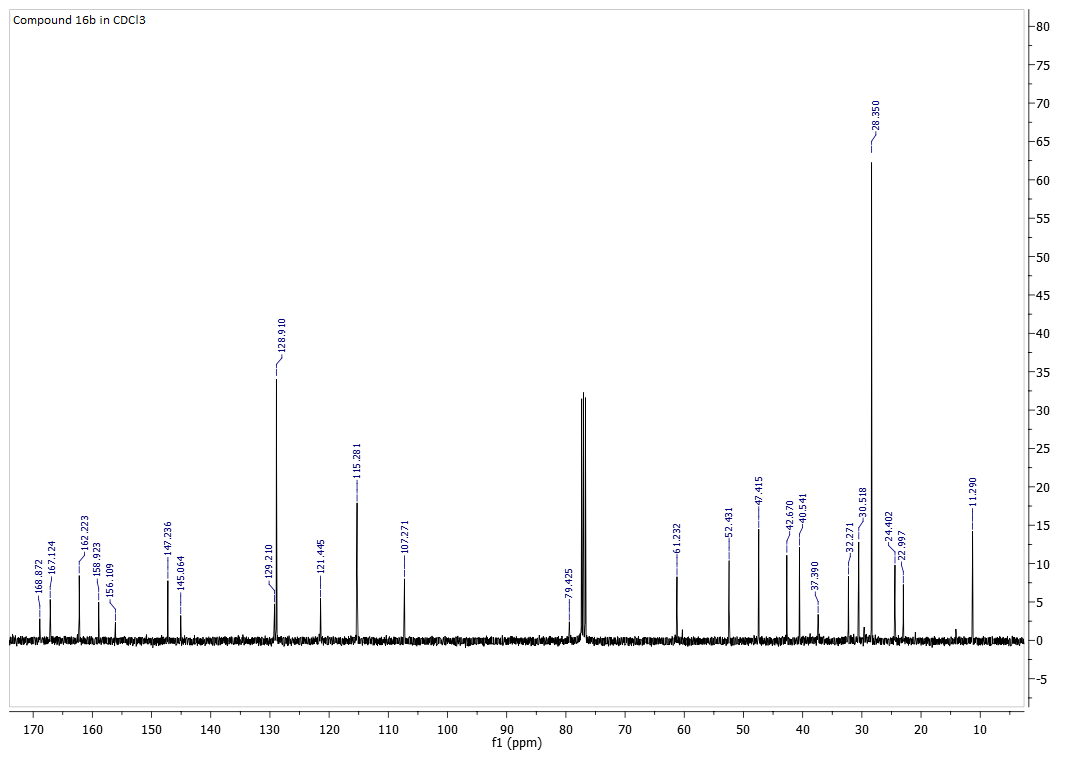


**Fig. S30** ^13^C-NMR spectrum of compound 16b.


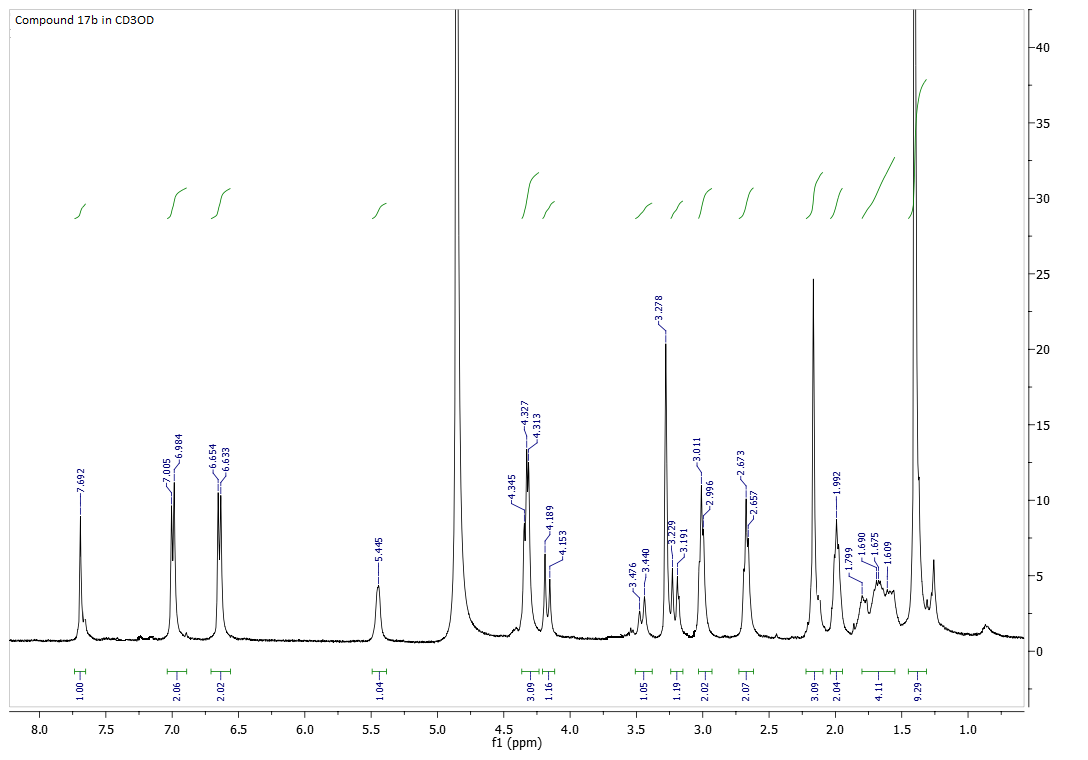


**Fig. S31** ^1^H-NMR spectrum of compound 17b.


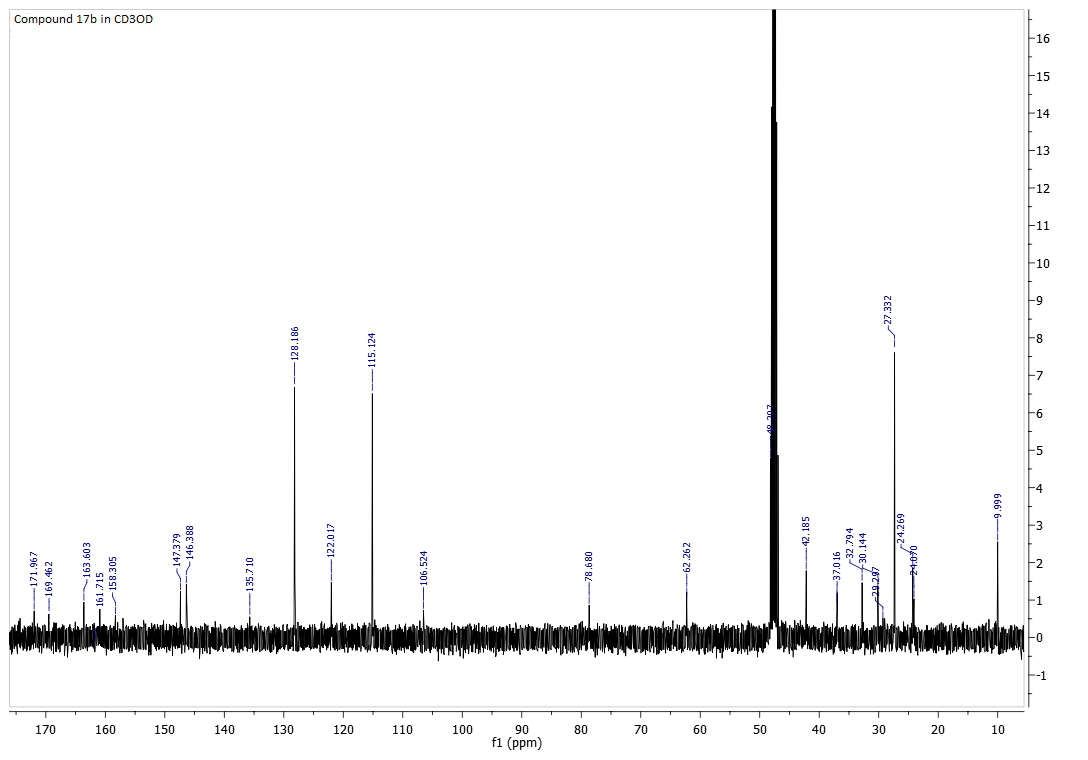


**Fig. S32** ^13^C-NMR spectrum of compound 17b.


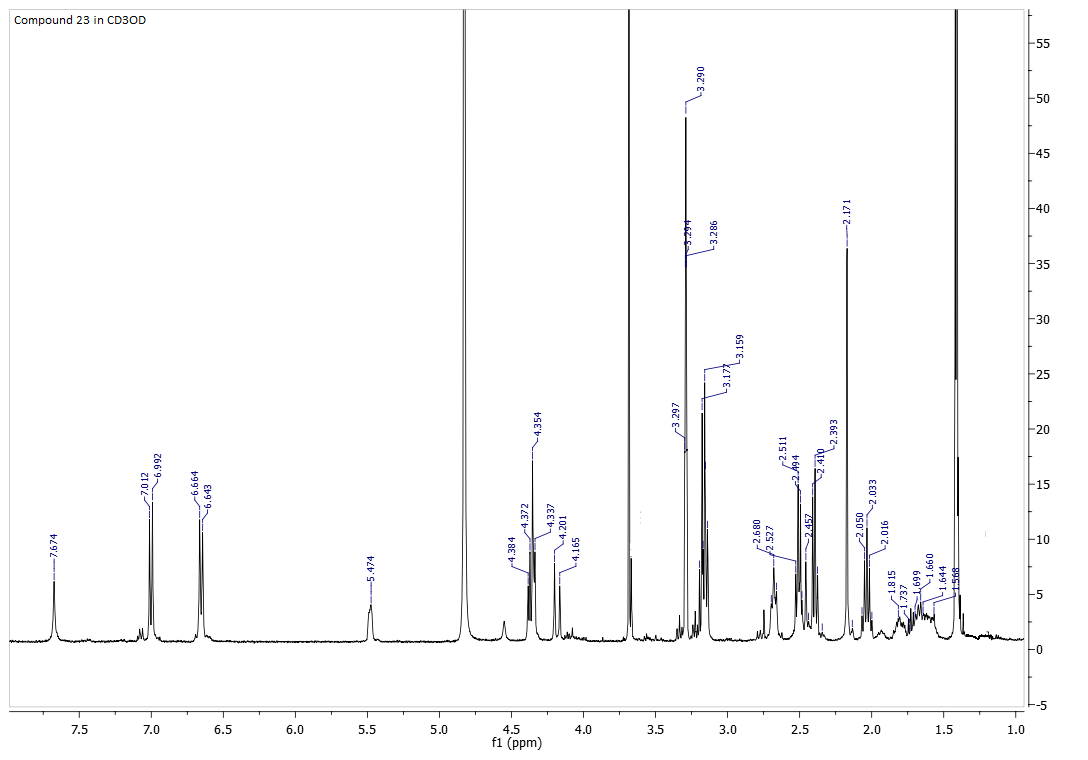


**Fig. S33** ^1^H-NMR spectrum of compound 23.


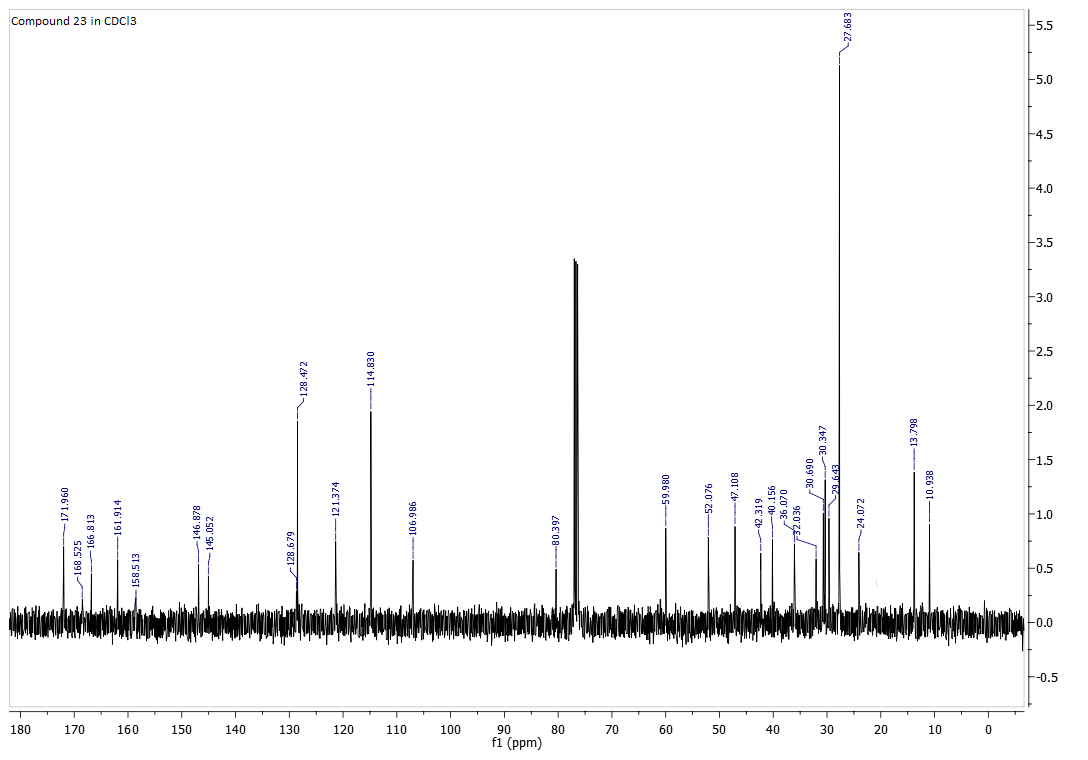


**Fig. S34** ^13^C-NMR spectrum of compound 23.


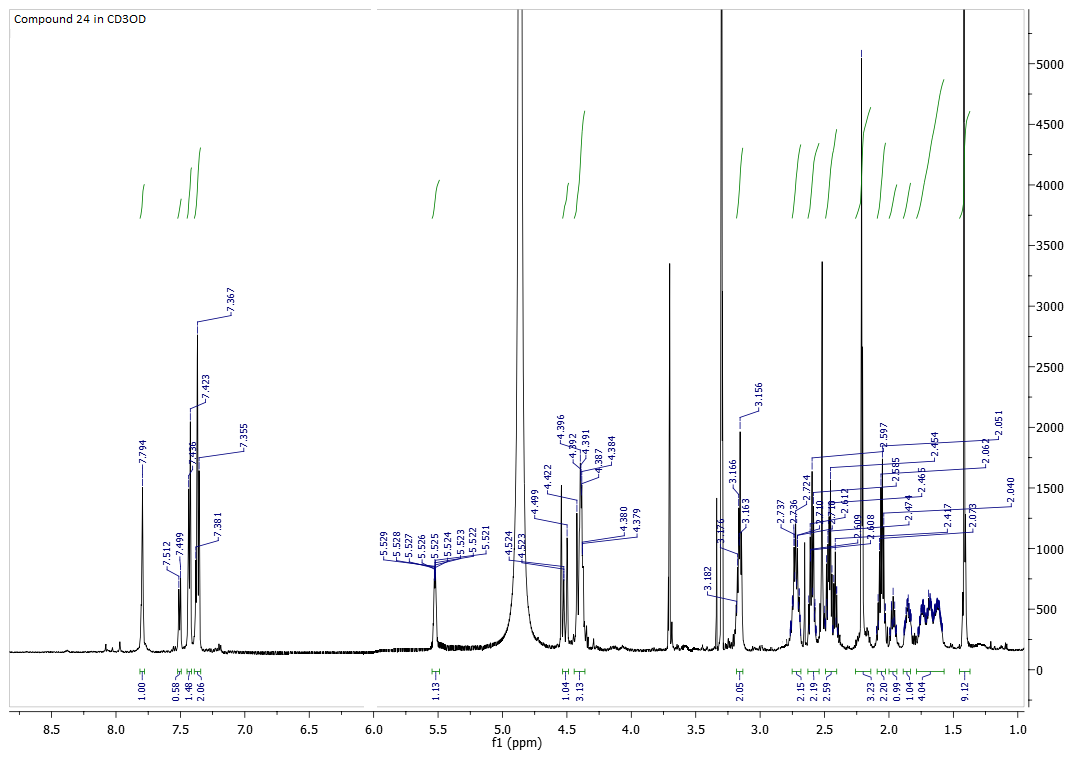


**Fig. S35** ^1^H-NMR spectrum of compound 24.


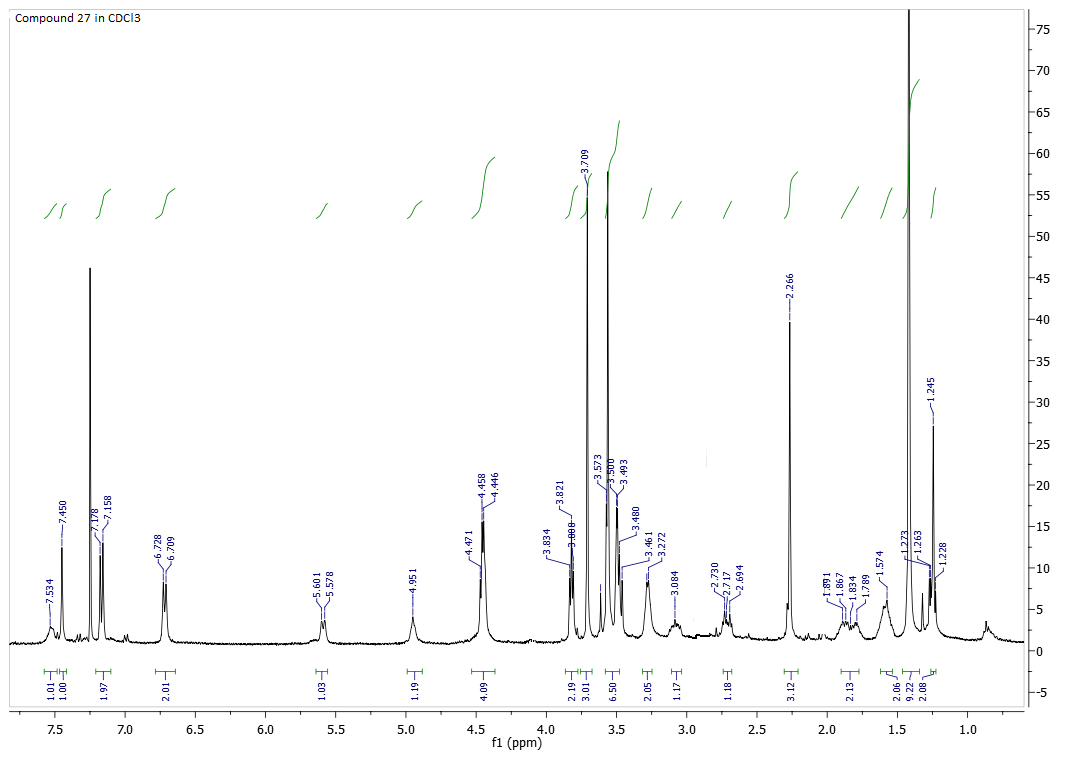


**Fig. S36** ^1^H-NMR spectrum of compound 27.


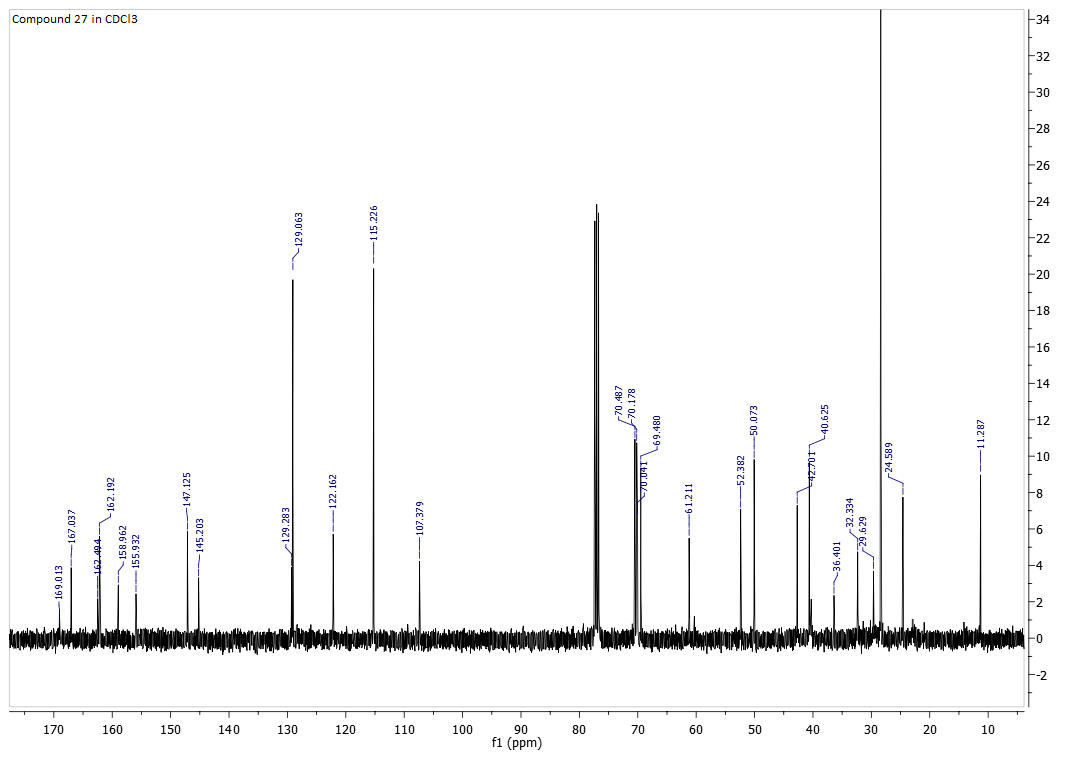


**Fig. S37** ^13^C-NMR spectrum of compound 27.


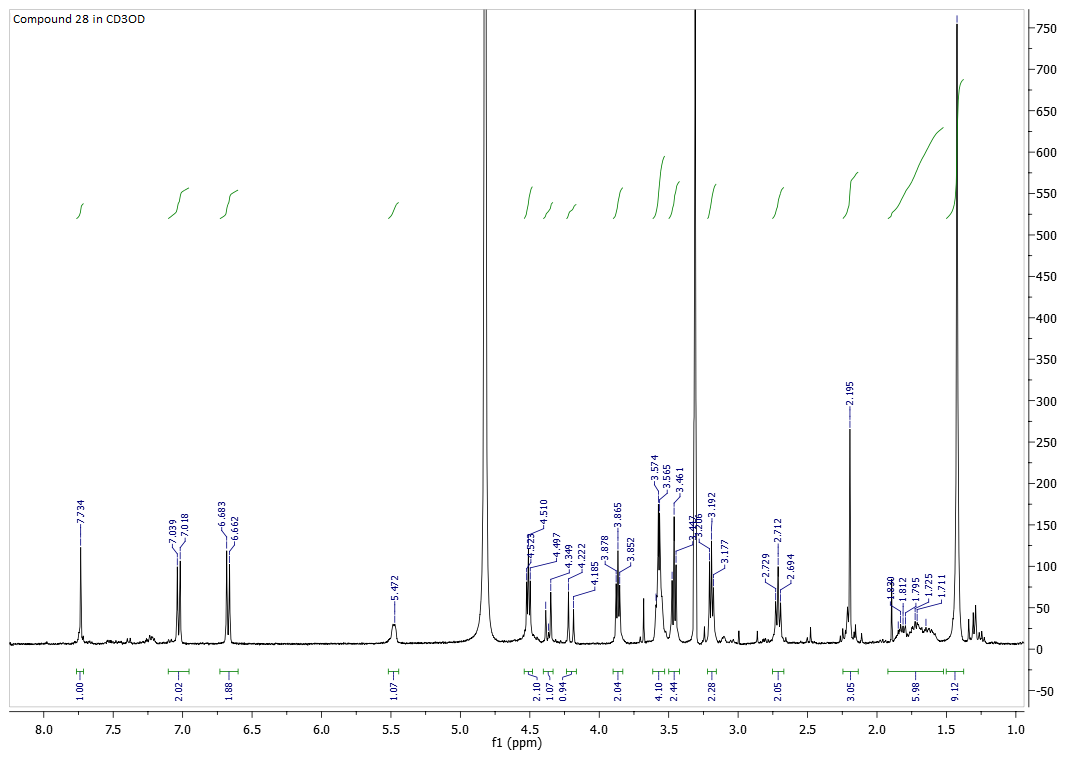


**Fig. S38** ^1^H-NMR spectrum of compound 28.


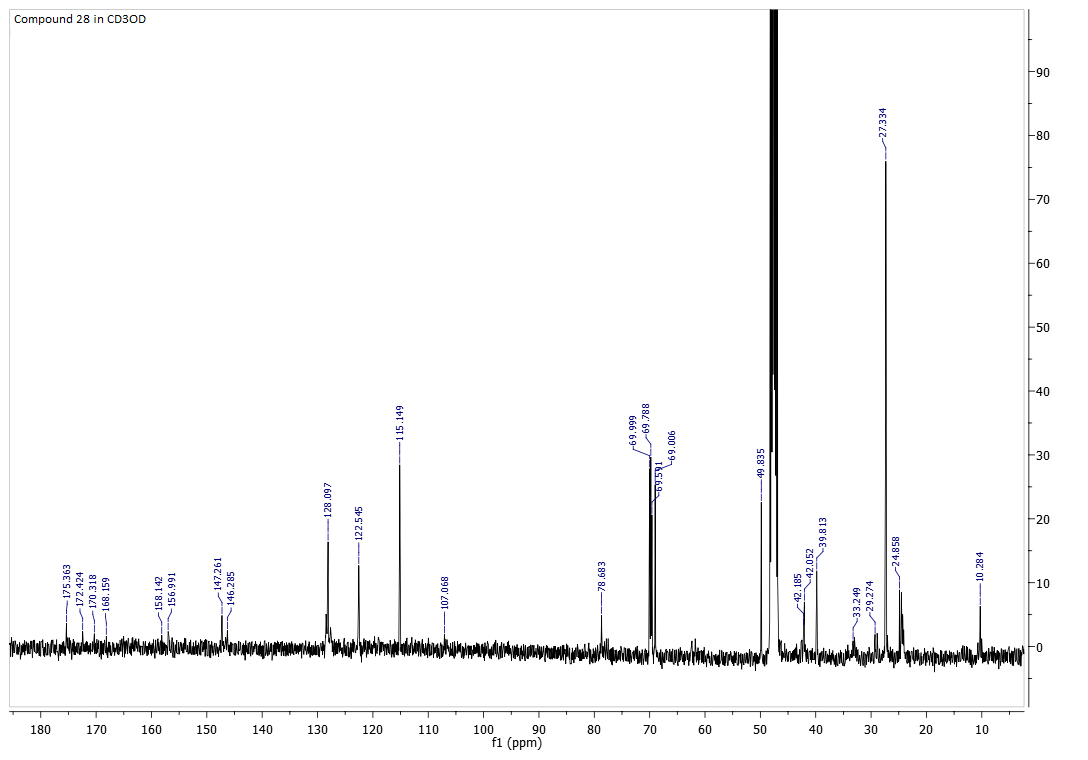


**Fig. S39** ^13^C-NMR spectrum of compound 28.





**totERK1/2**

**pERK1/2**





**Fig. S40** Full-length blots of cropped blots, reported in Figure 2a of the manuscript, related to the effect of compounds 17b and 24 on α5β1 integrin-mediated phosphorylation of pERK1/2 and totERK1/2 in K562 cells. Cells were serum-starved in RPMI-1640 containing 1% FBS for 16 h; then cells were preincubated with different concentrations of the antagonist 17b (10-7-10-9 M) or its vehicle for 1h in suspension and then were allowed to adhere for 1h on fibronectin (FN). Cells treated with the agonist 24 (10-7-10-9 M) were not incubated with fibronectin. Thereafter cells were lysed and lysates were analysed in Western blot using an antibody directed against phosphorylated ERK1/2 (pERK1/2) or total ERK1/2 (totERK1/2). Western blot showed that cells plated on FN had a much stronger signal for phosphorylated ERK1/2 than vehicle-treated cells. Compound 17b prevented FN-induced phosphorylation of ERK1/2 in a concentration-dependent manner, while agonist 24 significantly increased ERK1/2 phosphorylation.


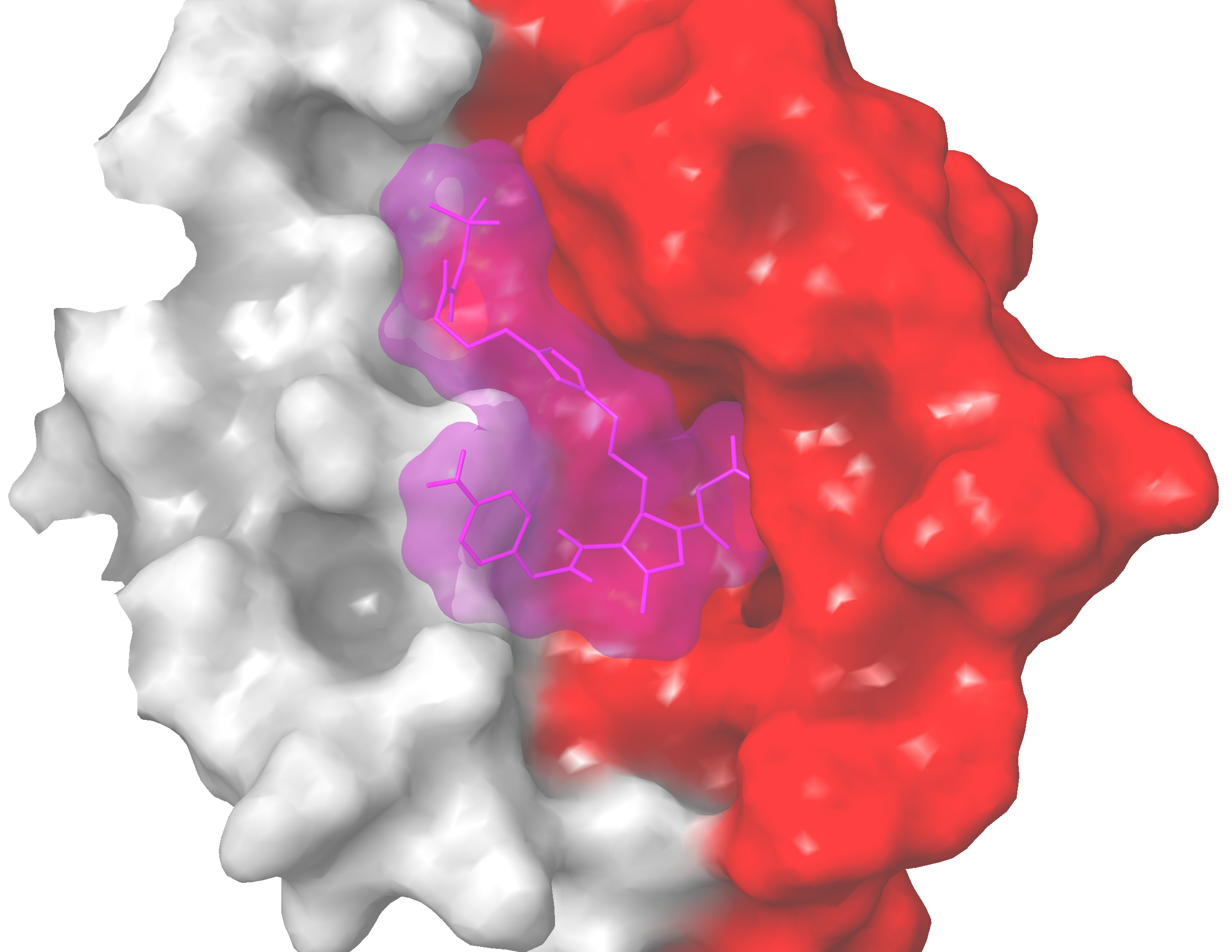

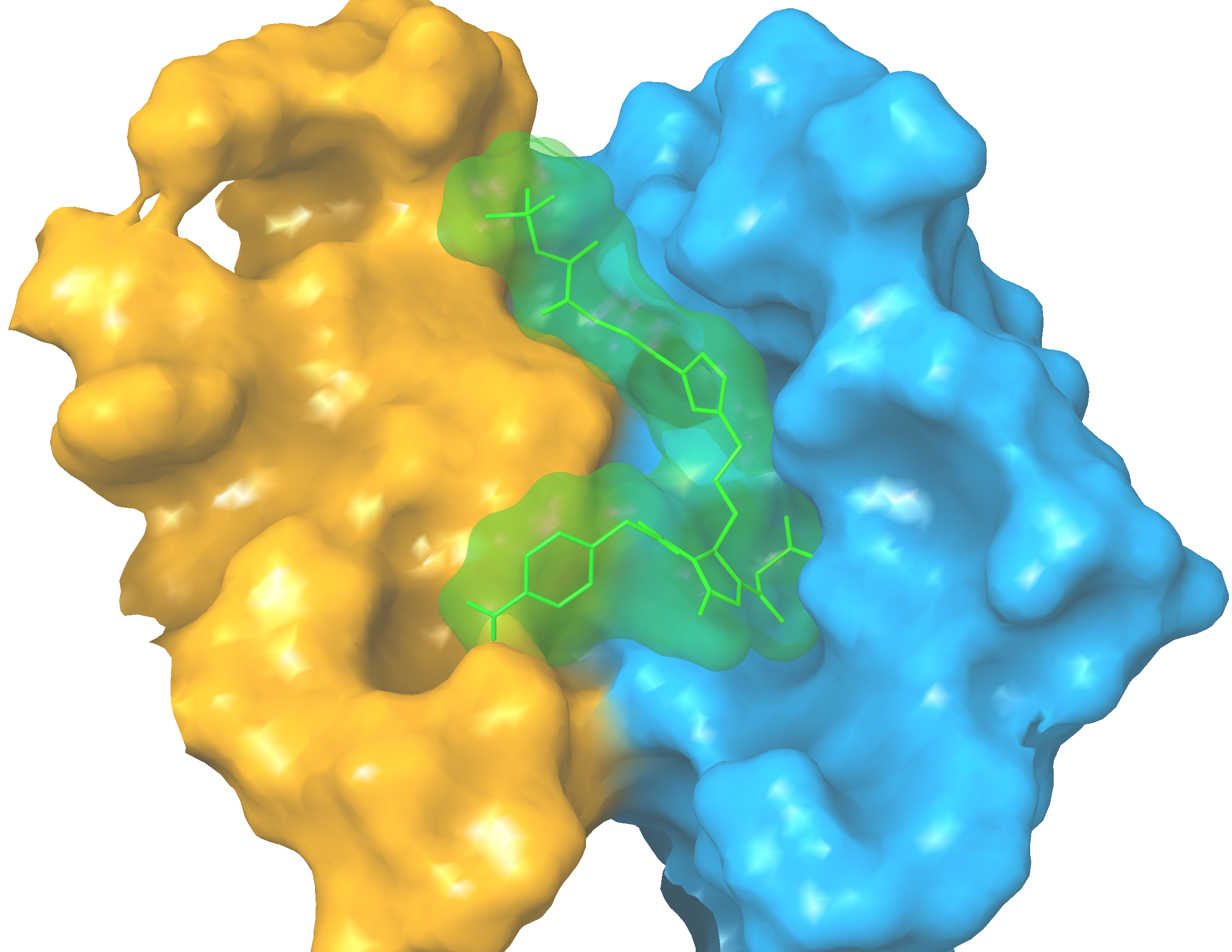

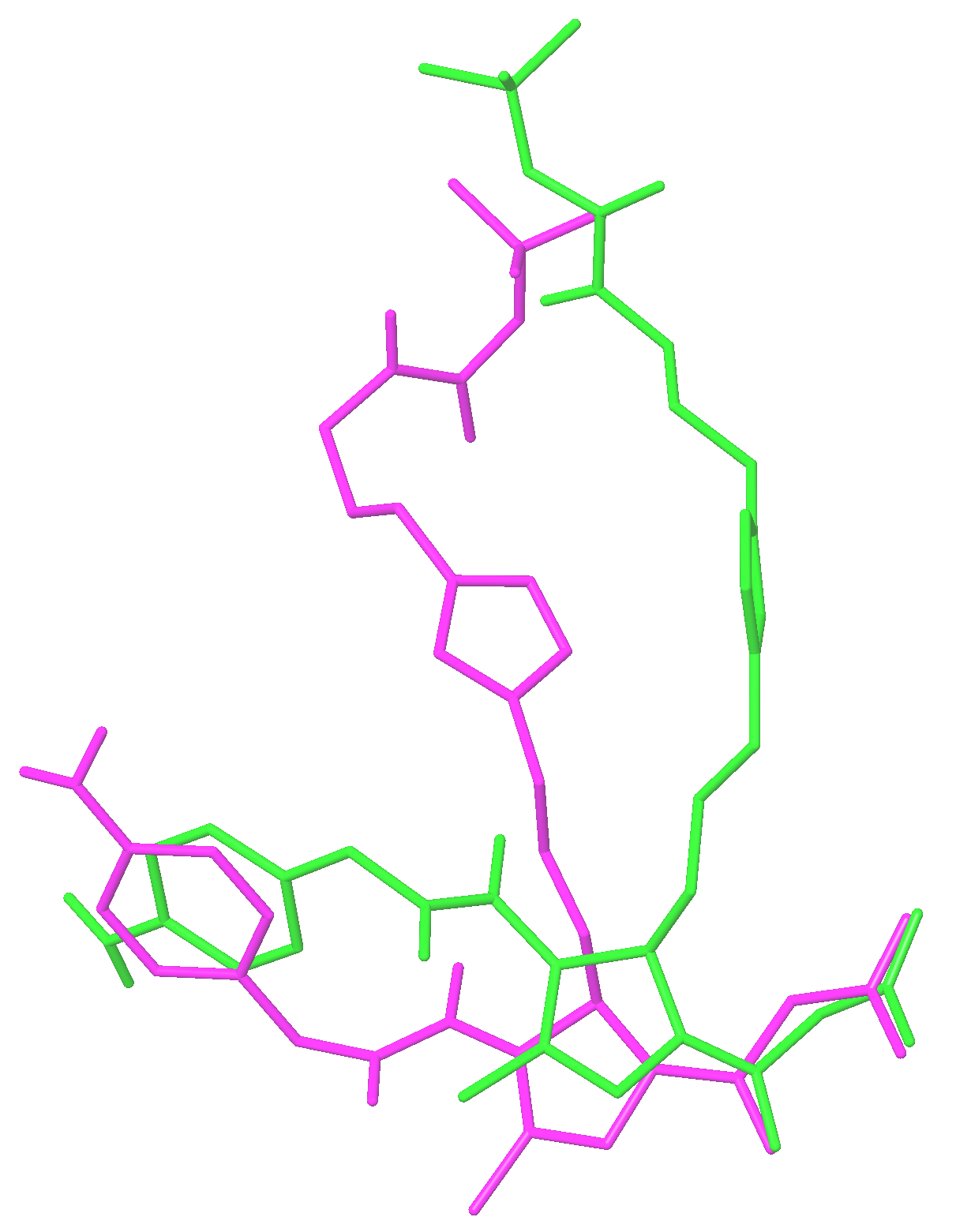


**B**

**A**

**Fig. S41** A) Docking best pose of (R)-17b (green) in α5β1 (α5 orange, β1 blue). Middle) Overlap of the best poses of (R)-17b in α5β1 (green) and αvβ3 (purple) deriving from the alignment of the two binding sites. B) Docking best pose of (R)-17b (purple) in αvβ3 (αv white, β3 red).

In particular, the mutation of α5-Trp157 into αv-Asp148 removes the stabilizing interaction of the triazole ring with the indole moiety of the Trp residue, while the mutations of Ser177, Gly223, Leu225 in β1 into Tyr166, Arg214, Arg216 in β3, respectively, reduce the size of the pocket at the αvβ3 interface, making it unsuitable to properly fit the long-chain substituent of 17b. Moreover, the mutation of β1-Lys182 into β3-Leu173 prevents the formation of polar interactions and decreases the number of favorable contacts. Based on these considerations, the selectivity of 17b towards α5β1 can be rationalized, as well as the decrease in activity displayed by the ligands depending on the features of the isoxazoline substituent. The image has been generated using Maestro graphical interface [Maestro, version 10.5, Schrödinger, LLC, New York, NY, 2016].

**Fig. S42** Calculated physicochemical properties for compound 8.

**Fig. S43** Calculated physicochemical properties for compound 17a.

**Fig. S44** Calculated physicochemical properties for compound 17b.

**Fig. S44** Calculated physicochemical properties for compound 20a.

**Fig. S46** Calculated physicochemical properties for compound 20b.

**Fig. S47** Calculated physicochemical properties for compound 24.

**Fig. S48** Calculated physicochemical properties for compound 26.

**Fig. S49** Calculated physicochemical properties for compound 28.

**Fig. S50** Calculated physicochemical properties for compound 30.

**Fig. S51** Calculated physicochemical properties for compound 31.

**Fig. S52** Calculated physicochemical properties for Cilengitide.
